# Investigation of the Mechanism of Cinnamaldehyde in Irritable Bowel Syndrome Based via Network Pharmacology, Molecular Docking, and Animal Experiments

**DOI:** 10.1002/pdi3.70017

**Published:** 2025-10-05

**Authors:** Qingyang Yu, Boqing Xu, Chunfang Dai, Yayan Pang, Zhifang Dong

**Affiliations:** ^1^ Department of Traditional Chinese Medicine Children's Hospital of Chongqing Medical University Chongqing China; ^2^ Growth, Development, and Mental Health of Children and Adolescence Center Ministry of Education Key Laboratory of Child Development and Disorders Chongqing Key Laboratory of Child Neurodevelopment and Cognitive Disorders Pediatric Research Institute National Clinical Research Center for Child Health and Disorders Children’s Hospital of Chongqing Medical University Chongqing China; ^3^ Guangzhou Women and Children's Medical Center Guangzhou Medical University Guangdong Provincial Clinical Research Center for Child Health Guangzhou China

**Keywords:** cinnamaldehyde, irritable bowel syndrome, molecular docking, monoamine oxidase B, network pharmacology

## Abstract

Irritable bowel syndrome (IBS) is a prevalent functional gastrointestinal disorder characterized by abdominal pain and changes in bowel habits. Cinnamaldehyde (CA) possesses anti‐inflammatory, antibacterial, and digestive‐regulatory properties. However, its therapeutic potential for IBS and mechanisms are not understood. We employed network pharmacology to identify potential targets and pathways of CA against IBS. Core targets were validated through molecular docking, and further verified in an IBS rat model induced by neonatal maternal separation (NMS) and water avoidance stress (WAS). Network pharmacology identified 139 potential targets of CA related to IBS. Gene Ontology (GO) enrichment analysis highlighted key biological processes, cellular components, and molecular functions. Kyoto Encyclopedia of Genes and Genomes (KEGG) analysis suggested involvement of pathways such as nitrogen metabolism, tyrosine metabolism, and cocaine addiction. A protein–protein interaction (PPI) network revealed 11 major targets, and molecular docking demonstrated strong binding affinities between CA and several targets, particularly *MAOB*, *PARP1*, *HDAC1*, *JAK2*, and *MMP2*. Animal experiments confirmed that CA significantly reduces *MAOB*, as well as TNF‐α, IL‐6, and IL‐1β levels, thereby alleviating visceral hypersensitivity and anxiety and depression‐like behaviors in IBS rats. These findings provide a scientific basis for developing CA as a potential natural therapeutic agent for IBS.

## Graphical abstract

Through network pharmacology and molecular docking, it has been discovered that CA can target *MAOB*, among other proteins, to exert a therapeutic effect in IBS. In vivo, CA lowered visceral hypersensitivity, anxiety and depression‐like behaviors, and fecal water content, highlighting its therapeutic potential for IBS via anti‐inflammatory pathways.

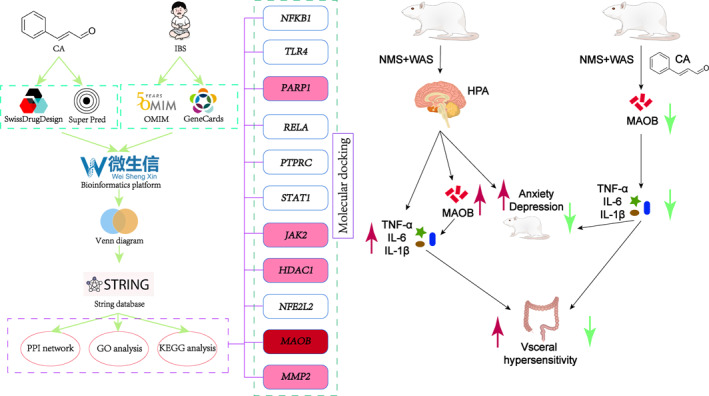

## 1 Introduction

Irritable bowel syndrome (IBS) is a chronic functional gastrointestinal disorder (FGID) characterized by intestinal dysfunction [1]. Clinically, it presents with recurrent abdominal pain and bloating, often accompanied by altered bowel habits, such as diarrhea, constipation, or an alternation between the two, and abnormal stool characteristics [2]. A definitive diagnosis requires the exclusion of organic lesions, and symptoms typically persist for more than 6 months. The global prevalence of IBS is estimated to be approximately 3%–5%, with a significantly higher incidence in women than in men. The female‐to‐male odds ratio is 1.8 (1.7, 2.0) in internet‐based surveys and 1.98 (2.0, 2.5) in household‐based surveys [3]. IBS imposes a considerable burden on individuals, families, and healthcare systems, significantly affecting patients' quality of life, increasing healthcare expenditures, and contributing to broader social and economic challenges [4]. For instance, the direct healthcare costs related to IBS in China were estimated at approximately 2 billion US dollar annually as of 2016, and this figure continues to rise [5]. The pathogenesis of IBS is multifactorial, with the dysfunction of the brain‐gut axis playing a central role [6, 7]. Currently, there is no definitive cure for IBS. Conventional treatments, such as antispasmodics, antibiotics, and probiotics, often suffer from limited efficacy, lack of target specificity, and undesirable side effects [8]. Thus, there is growing interest in natural compounds and precision therapies as promising avenues for IBS treatment.

Cinnamaldehyde (CA), also known as trans‐cinnamaldehyde, is a yellow viscous aromatic aldehyde abundantly found in cinnamon bark. CA is the major active component of cinnamon, which serves not only as a spice but also as a traditional Chinese medicinal herb. Modern pharmacological studies have shown that CA possesses a wide range of biological activities, including anti‐inflammatory [9, 10], antioxidant [11], anti‐bacterial [12], antiviral [13], and hepatoprotective [14] effects. Notably, CA can significantly reduce the secretion of pro‐inflammatory cytokines such as TNF‐α and IL‐6 in the treatment of recurrent aphthous stomatitis [15]. In addition, CA has demonstrated protective effects against gastric mucosal injury by modulating apoptosis, autophagy, and ferroptosis through the PI3K/AKT signaling pathway [16]. However, it remains unclear whether CA exerts therapeutic effects on IBS, particularly through anti‐inflammatory or other mechanisms.

Network pharmacology, first proposed in concept by Chinese scholars in 1999 [17] and formally named by British pharmacologist Andrew L. Hopkins in 2007 [18], provides a systems‐level approach for understanding the pharmacological actions of natural products. It involves constructing and analyzing biomolecular interaction networks to predict drug–target interactions and disease mechanisms [19]. Molecular docking, when integrated with network pharmacology, can further elucidate the binding modes and affinities of drug candidates to their potential targets at the molecular level [20]. Together, these techniques enable the efficient and precise identification of key targets and mechanisms involved in drug action.

In this study, we employed network pharmacology to identify potential targets and signaling pathways through which CA may act against IBS. These core targets were subsequently validated using molecular docking and in‐vivo experiments. This integrative approach provides new insights into the mechanism of CA in IBS intervention and lays the groundwork for the development of novel therapeutic agents and precision treatment strategies.

## 2 Methods and Materials

### 2.1 Network Pharmacology Analysis

#### 2.1.1 Target Identification for CA and IBS

The SMILES notation for CA was obtained from the PubChem database (https://pubchem.ncbi.nlm.nih.gov/) [21]. CA‐related targets were identified using the Swiss Target Prediction (http://www.swisstargetprediction.ch/) [22] and Super Pred (https://prediction.charite.de/) [23] databases. Genes associated with IBS were retrieved from the OMIM (https://omim.org/) [24] and GeneCards (https://www.genecards.org/) [25] databases using the keyword “irritable bowel syndrome”, which is the standard English full name for the disease. The intersecting targets of CA and IBS targets were identified using the bioinformatics platform (http://www.bioinformatics.com.cn/).

#### 2.1.2 Construction of the Protein–Protein Interaction (PPI) Network

The intersecting targets were uploaded to the STRING database (https://string‐db.org/) [26], with a minimum interaction score set at medium confidence (0.400). Duplicates and unrelated targets were removed, and the drug–disease–target PPI network was visualized using Cytoscape version 3.10.3 [27].

#### 2.1.3 Drug–Disease–Target Network Analysis

The identified intersection targets were used to construct a “drug–disease–target” network. The drugs, disease, and targets were imported into Cytoscape 3.10.3 for visualization.

#### 2.1.4 Gene Ontology (GO) and Kyoto Encyclopedia of Genes and Genomes (KEGG) Enrichment Analyses

GO function annotation and KEGG pathway enrichment analyses were performed using the Metascape database (https://metascape.org/) [28].

### 2.2 Molecular Docking Analysis

Compound structures in SDF format were downloaded from the PubChem database and preprocessed by dehydration and hydrogenation using AutoDock Vina. The ligands were then exported in PDBQT format. Core protein structures with lower resolution were obtained from the PDB database (https://www.rcsb.org) [29], followed by dehydration, ligand removal, and hydrogenation using PyMol. AutoDock Vina was used to define and optimize the receptor binding pockets for molecular docking. The docking results were converted into PDB format using Open Babel 3.1.1 and visualized with PyMOL.

### 2.3 Animal Experiment

#### 2.3.1 Chemicals

CA was purchased from Shanghai Aladdin Biochemical Technology Co. LTD. ELISA kits for *MAOB*, TNF‐α, IL‐6, and IL‐1β were obtained from Chongqing Jiaming Biotechnology Co. LTD.

#### 2.3.2 Animals and Feeding Conditions

Ten litters of 1‐ to 2‐day‐old male Sprague–Dawley (SD) rats, with no apparent congenital defects or abnormal body weight, were bred in a specific pathogen‐free (SPF) environment with a controlled room temperature of (25 ± 2) °C and relative humidity of (50 ± 10)% at the Animal Experimental Center of the Children's Hospital of Chongqing Medical University. Each litter, consisting of five pups, was fed by a single dam, totaling 50 pups. All experimental protocols were approved by the Ethics Committee of Children's Hospital of Chongqing Medical University (approval number: CHCMU‐IACUC20240508003), and every effort was made to minimize both the animal suffering and the number of animals used. Animals were excluded from the study if they died before the experiment concluded.

#### 2.3.3 Grouping and Model Establishment

On Day 1, two litters of pups were randomly selected to form the blank control group (BC), whereas the remaining litters underwent NMS. On Day 22, upon the completion of NMS, the remaining 8 litters were randomly assigned to four groups by an independent experimenter using the random number table method. These groups comprised the IBS model control group (MC) and three groups treated with CA at low (CA‐L), medium (CA‐M), and high (CA‐H) doses. A total of 10 animals were allocated to each group to ensure statistical robustness while adhering to ethical guidelines. From postnatal day 1–21, the BC group received normal care, whereas the other groups underwent maternal separation from 9:00 a.m. to 12:00 p.m. daily [30]. On day 22, all pups were weaned. From day 57 to day 66, rats in the MC and CA groups were subjected to 1 h of water avoidance stress (WAS) daily. This involved placing the rats on a 10 × 10 cm plexiglass platform, positioned 1 cm above the water surface. The platform was situated in the center of a tank filled with water, ensuring the rats could not reach the edge [31].

#### 2.3.4 Treatment and Monitoring

**FIGURE 1:**
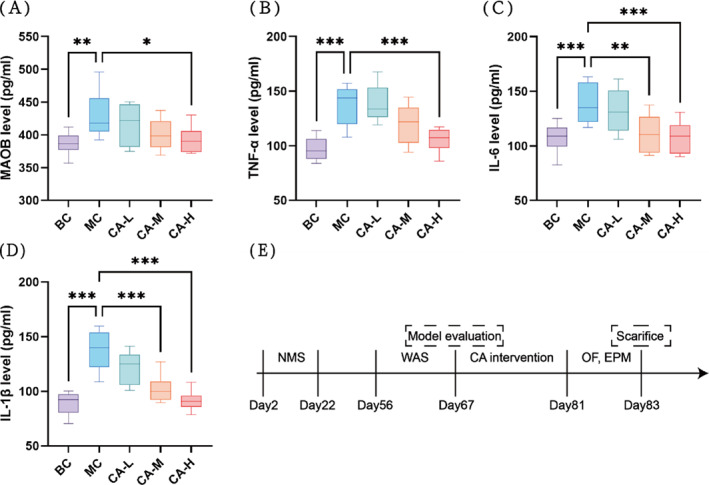
Effects of CA on inflammatory cytokines in IBS model rats. (A) *MAOB* levels. (B) TNF‐α levels. (C) IL‐6 levels. (D) IL‐1β levels. (E) Schematic diagram of the experimental procedure. EPM, elevated plus maze test; OF, Open field test.

Starting from day 67, rats in the BC and MC groups were gavaged with 10 mL/kg of deionized water daily. The CA‐L, CA‐M, and CA‐H groups received 10 mg/kg, 20 mg/kg, and 40 mg/kg of CA, respectively. Body weight was recorded on days 2, 56, 67, 74, and 81. Food intake and fecal water content were measured on days 56, 67, 74, and 81. Feces were collected in metabolic cages, weighed fresh, dried, and reweighed. Fecal water content was calculated as: fecal water content (%) = (wet weight ‐ dry weight)/wet weight × 100%. Abdominal withdrawal reflex (AWR) scores were assessed on days 56, 67, and 81. At the end of the study, rats were fasted for 12 h, anaesthetized, and euthanized. Colon tissues were excised, rinsed with saline, snap‐frozen in liquid nitrogen, and stored at −80°C. No animals died prematurely before the conclusion of the experiment, and data from each animal were included in the final statistical analysis. A schematic overview of the experimental workflow is shown in Figure 1E.

#### 2.3.5 AWR Score

AWR scores were evaluated before (day 56) and after water avoidance stress (day 67), as well as two weeks following the CA intervention (day 81). Rats were fasted for 24 h before the experiment. A 6Fr catheter and 1 mL syringes were used for measurement preparation, where water was injected into the balloon to ensure no gas remained. The syringe position during embolization and water volume were noted. Before measurement, rats' tails were lifted for 30 s to facilitate defecation. They were then placed in an ether‐saturated tank and monitored until anaesthetized. A glycerin‐coated a urinary catheter was inserted 6 cm into the rat's intestines, secured to the tail, and placed in plastic boxes. After a 10–15 min recovery, air was injected into the balloon in increments of 0.2 mL, 0.4 mL, 0.6 mL, and 0.8 mL, with reactions observed. Each water injection was tested three times and averaged, following the AWR scoring criteria. The AWR scoring criteria were shown in Supporting Information S1: Table S1 [32].

#### 2.3.6 Open Field (OF) Test

OF behavior device (100 × 100 × 40 cm) was utilized to assess anxiety and depression‐like behaviors in rats. The experimental rats were introduced to the device one at a time, one day prior to the experiment, to acclimate them to the experimental environment. On the day of the formal experiment, the rats were sequentially placed in the central area of the device, and the ANY‐maze animal behavior software (Wood Dale, Illinois, Stoelting, USA) was employed to observe and record the total distance, center time, center entries, and the number of standing within 5 min. Before each rat was subjected to the experiment, the experimental apparatus was cleaned with 75% alcohol to eliminate the influence of residual odors from the previous rat on the experimental results.

#### 2.3.7 Elevated Plus Maze (EPM) Test

EPM comprises two open arms and two closed arms, with the latter featuring a 40 cm high wall, arranged at right angles. Each arm measures 50 cm in length. During the test period, rats were placed in a central area measuring 10 cm by 10 cm, located 100 cm above the ground, for a 5‐min period of free exploration. The frequency of entries into the open arms and the duration spent in them were recorded using ANY‐maze ethology software (Wood Dale, Illinois, Stoelting, USA). To maintain a clean and consistent experimental environment for each rat, the equipment was sanitized with 75% alcohol before each rat was introduced.

#### 2.3.8 Enzyme‐Linked Immunosorbent Assay (ELISA)

Levels of *MAOB*, TNF‐α, IL‐6, and IL‐1β in colon tissue were determined according to the protocols provided with the ELISA kit.

#### 2.3.9 Statistical Analysis

Data were expressed as mean ± standard deviation (SD) with 95% confidence intervals (95% CIs). One‐way or two‐way ANOVA was used for statistical analysis. Graphs were generated using GraphPad Prism 10.4.0 (GraphPad Software Inc., San Diego, CA, USA). Statistical significance was defined as a *p* value < 0.05. Outcome assessments and data analyses were conducted by independent panelists who were unaware of the group assignments.

## 3 Results

### 3.1 Network Pharmacology Analysis

**FIGURE 2:**
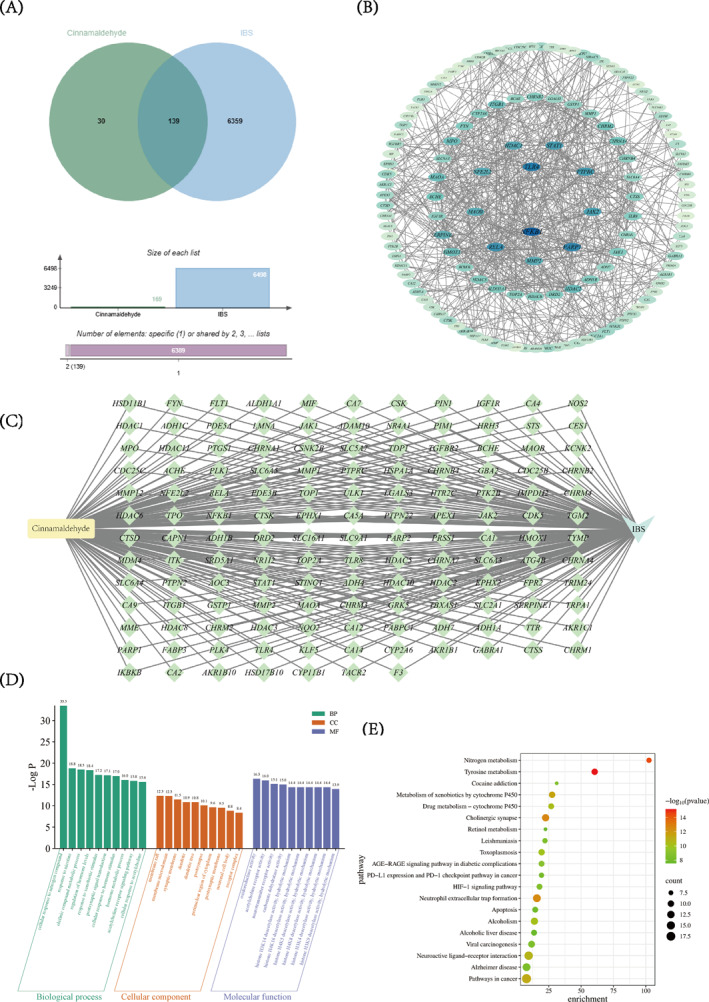
Network pharmacology analysis of CA intervention in IBS. (A) Venn diagram illustrating the intersection of targets related to CA and IBS. A total of 169 CA‐targets and 6498 IBS‐related targets were identified, with 139 overlapping targets. (B) PPI network of the intersecting targets. Nodes represent proteins, and edges represent interactions between them. The number of edges indicates interaction strength, with darker nodes representing higher degrees of connectivity. (C) “Drug–disease–target” interaction network. (D) GO enrichment analysis. The Y‐axis represents fold enrichment, and the X‐axis lists the top 30 terms. Terms are grouped by category: biological process (green), cellular component (orange), and molecular function (purple). (E) KEGG pathway enrichment analysis (performed using Metascape database). Pathways are plotted on the Y‐axis and false discovery rate on the X‐axis. *p* values are indicated by color gradients, and bubble size corresponds to the number of genes enriched in each pathway.

**FIGURE 3:**
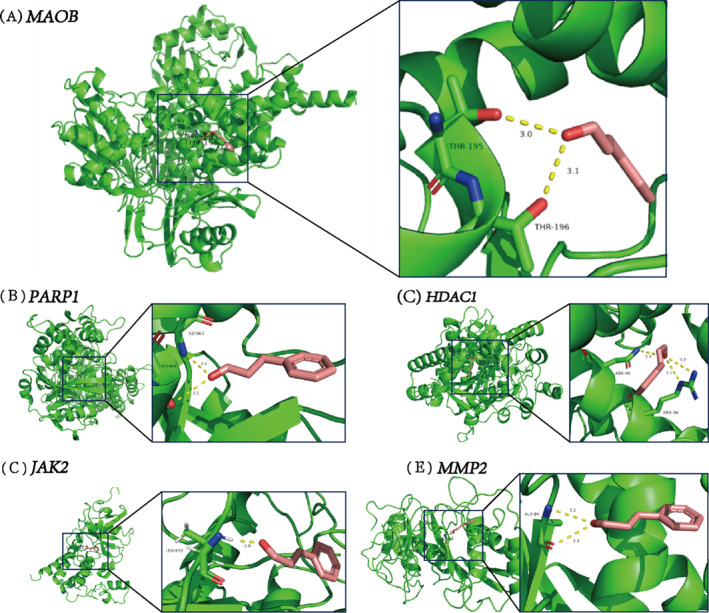
Molecular docking map. (A) *MAOB*. (B) *PARP1*. (C) *HDAC1*. (D) *JAK2*. (E) *MMP2*.

To explore the potential targets of CA in the treatment of IBS, we conducted a network pharmacology analysis (Figures 2 and 3). The molecular structure of CA (C = CC1 = CC = CC = C1C = O) was submitted to Swiss Target Prediction and Super Pred database for target prediction. After standardization using the UniProt database and removal of duplicates, 169 potential targets of CA were idendified. “Irritable bowel syndrome” was used as a keyword to retrieve disease‐related genes from the OMIM and GeneCards databases. After applying the UniProt specifications and removing duplicates, a total of 6498 IBS‐related disease targets were obtained.

The intersection of the predicted drug targets and disease‐related genes was determined using the Microbiology Letter platform, resulting in 139 overlapping targets (Figure 2A). A PPI network was then constructed, comprising 135 nodes and 660 edges, with the 11 nodes ranked by degree value highlighted (Figure 2B). Among these, NFKB1 exhibited the highest number of connections (35), followed by TLR4 (31), *PARP1* (29), RELA (27), PTPRC (25), STAT1 (25), *JAK2* (24), *HDAC1* (23), NFE2L2 (22), *MAOB* (22), and *MMP2* (22) (Table 1).

**TABLE 1:** The core targets of CA against IBS.

Name	Degree
NFKB1	35
TLR4	31
*PARP1*	29
RELA	27
PTPRC	25
STAT1	25
*JAK2*	24
*HDAC1*	23
NFE2L2	22
*MAOB*	22
*MMP2*	22

A “drug–disease–target” interaction network was subsequently constructed using these intersection targets, which consisted of 141 nodes and 278 edges. In this network, yellow nodes represented CA, green nodes represented common targets of the drug and disease, and the wathet node represented IBS. Edges denoted interactions among these entities (Figure 2C). To systematically investigate the mechanisms by which CA may exert therapeutic effects on IBS, GO enrichment and KEGG pathway enrichment analyses were performed using the Metascape platform. GO enrichment yielded a total of 1198 terms, including 936 biological processes, 89 cellular components, and 173 molecular functions. The top 10 enriched GO items were presented in Figure 2D. These results suggest that CA may influence IBS through biological processes such as cellular responses to nitrogen compounds, responses to nicotine and xenobiotic stimuli, olefinic compounds metabolism, regulation of hormone levels, and postsynaptic signal transduction. In terms of cellular components, CA‐targeted proteins were enriched in membrane rafts, membrane microdomains, synaptic membranes, and dendrites. The molecular functions were mainly related to oxidoreductase activity, acetylcholine receptor activity, neurotransmitter receptor activity, and carbonate dehydratase activity (Figure 2D). KEGG pathway analysis revealed 125 enriched signaling pathways. The figure below presents the top 20 pathways. The top 20 pathways are shown in Figure 2E, with nitrogen metabolism, tyrosine metabolism, and cocaine addiction identified as the most significantly enriched. However, these findings require further validation through experimental studies.

### 3.2 Molecular Docking

To further validate the interaction between CA and the core targets identified via network pharmacology, molecular docking simulations were conducted to evaluate the binding modes and affinities. It is generally accepted that a lower binding energy indicates a more stable interaction between the ligand and the target protein, reflecting a stronger likelihood of biological activity. As shown in Table 2, all 11 core targets demonstrated spontaneous binding to CA, with eight of them exhibiting binding affinities less than −5.0 kcal/mol. The docking results showed that CA interacts with key amino acid residues through the formation of hydrogen bonds and hydrophobic interactions. As illustrated in Figure 3, CA forms hydrogen bonds with key residues of *MAOB* (Figure 3A), *PARP1* (Figure 3B), *HDAC1* (Figure 3C), *JAK2* (Figure 3D), and *MMP2* (Figure 3E). In each docking model, the red structures represents CA molecule, the green structure represents the target protein, and yellow dashed lines indicate hydrogen bond interactions. Among all targets, *MAOB* exhibited the strongest binding affinity to CA, with a docking score of −7.0 kcal/mol.

**TABLE 2:** Core target binding abilities (kcal/mol).

Target	Binding affinity
NFKB1	−4.5
TLR4	−5.4
*PARP1*	−6.3
RELA	−5.1
PTPRC	−4.1
STAT1	−5.1
*JAK2*	−6.2
*HDAC1*	−5.8
NFE2L2	−4.4
*MAOB*	−7.0
*MMP2*	−6.4

### 3.3 CA Enhances Food Intake in IBS Rats (*n* = 10)

**FIGURE 4:**
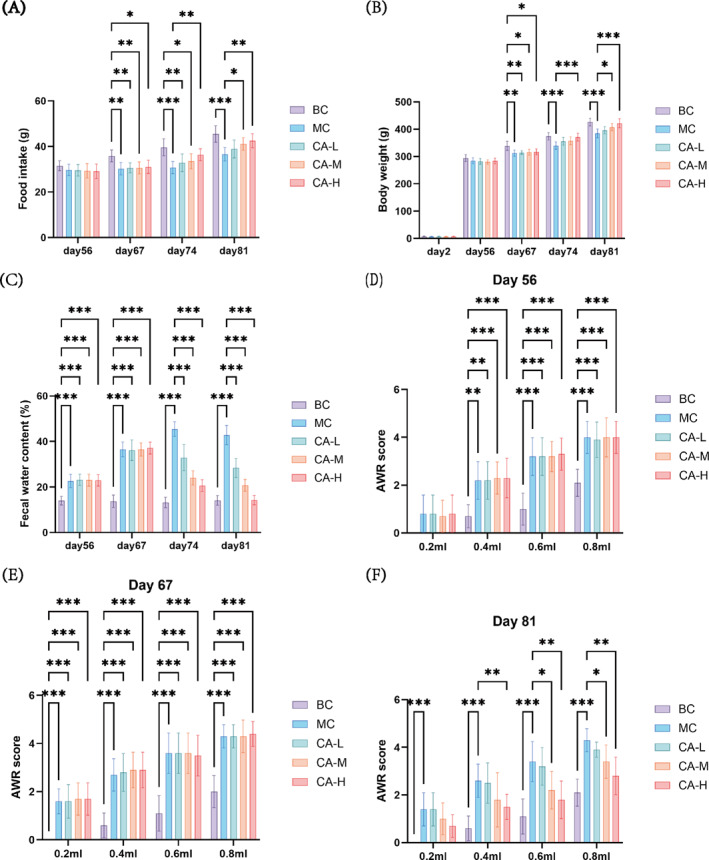
Effects of CA on physiological parameters in IBS model rats. (A) Food intake. (B) Body weight. (C) Fecal water content. (D) AWR scores on day 56. (E) AWR scores on day 67. (F) AWR scores on day 81. (*p < 0.05, **p < 0.01, and ***p < 0.001).

On day 56, prior to the WAS procedure, there were no significant differences in food intake among the BC, MC, CA‐L, CA‐M, and CA‐H, following neonatal maternal separation (NMS) and 5 weeks of cage feeding (Figure 4A). On day 67, after 10 days of WAS, the food intake in the MC, CA‐L, CA‐M, and CA‐H groups was significantly lower than that in the BC group (*p* < 0.01; Figure 4A). No significant differences were observed among the MC and CA‐treated groups at this time point. On day 74, following one week of CA intervention, the food intake in the CA‐H group was significantly higher than that in the MC group (*p* < 0.01; Figure 4A), whereas no significant differences were found between the CA‐L or CA‐M groups and the MC group. After two weeks of CA intervention (day 81), both the CA‐M and CA‐H groups exhibited significantly increased food intake compared to the MC group (CA‐M: *p* < 0.05; CA‐H: *p* < 0.01; Figure 4A), whereas no significant difference was observed between the CA‐L and MC groups. These findings suggest that CA can enhance appetite and increase food intake in rats with IBS.

### 3.4 CA Promotes Body Weight Gain in IBS Rats (*n* = 10)

There was no statistically significant difference in body weight among groups on day 2 and day 56 (Figure 4B). On day 67, following WAS, the body weights of the MC and CA‐treated groups were significantly lower than that of the BC group (MC: *p* < 0.01; CA‐L: *p* < 0.01; CA‐M: *p* < 0.05; CA‐H: *p* < 0.05; Figure 4B). Between the MC and CA‐treated groups (CA‐L/M/H), no significant variations in body weight were observed. On day 74, the CA‐H group experienced a significant increase in body weight compared to the MC group, and the difference was statistically significant (*p* < 0.001; Figure 4B). On day 81, both the CA‐M and CA‐H groups experienced a significant increase in body weight compared to the MC group, with the difference being statistically significant (Figure 4B). These results suggest that CA can improve the low body weight observed in IBS model rats.

### 3.5 CA Reduces Fecal Water Content in IBS Rats (*n* = 10)

On days 56 and 67, the fecal water content in the MC and CA‐treated groups (CA‐L/M/H) was significantly higher than that in the BC group (*p* < 0.001; Figure 4C), with no significant differences among the MC and CA‐L/M/H groups. After 1 and 2 weeks of CA intervention, fecal water content in all CA‐treated groups was significantly lower than that in the MC group (*p* < 0.001; Figure 4C). These results suggest that CA improves stool consistency and reduces the incidence of loose stools in IBS rats.

### 3.6 CA Reduces AWR Scores in IBS Rats (*n* = 10)

On day 56, there were no significant differences in AWR scores among the groups when 0.2 mL of water was injected into the balloon. However, when 0.4 mL, 0.6 mL, and 0.8 mL of water were injected, the AWR scores of the MC, CA‐L/M/H groups were significantly higher than those of the BC group (*p* < 0.01; Figure 4D). No significant differences were observed among the MC and CA‐treated groups. On day 67, regardless of the water volume injected (0.2 mL, 0.4 mL, 0.6 mL, or 0.8 mL), the AWR scores of the MC and CA‐L/M/H groups were all significantly higher than those of the BC group (*p* < 0.001; Figure 4E), with no statistical differences among the MC and CA‐treated groups. After two weeks of CA intervention, no significant differences in AWR scores were observed among the CA‐L/M/H and MC groups when 0.2 mL of water was used. However, with 0.4 mL of water, the AWR score of the CA‐H group was significantly lower than that of the MC group (*p* < 0.01). When 0.6 and 0.8 mL of water were administrated, both the CA‐M and CA‐H groups showed significantly reduced AWR scores compared to the MC group (CA‐M: *p* < 0.05; CA‐H: *p* < 0.01; Figure 4F). These results suggest that CA can reduce AWR score and alleviate visceral hypersensitivity in IBS model rats.

### 3.7 CA Alleviates Anxiety and Depression‐Like Behaviors in IBS Rats (*n* = 10)

**FIGURE 5:**
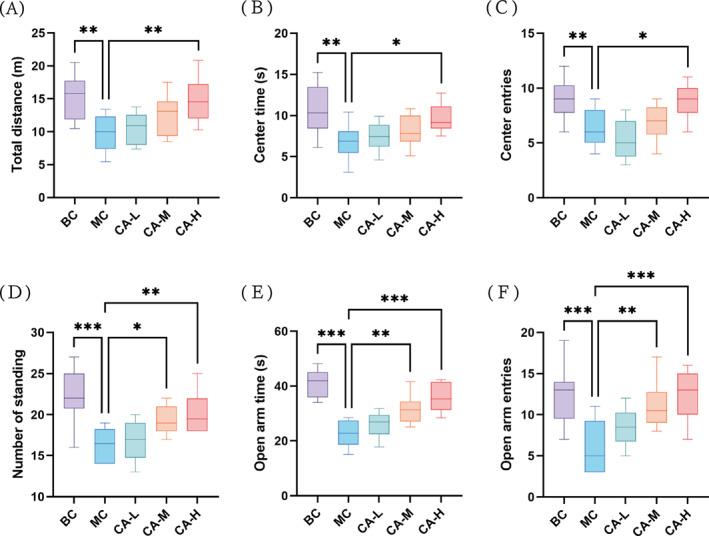
Effects of CA on anxiety and depression‐like behaviors in IBS rats. (A) Total distance in OF. (B) Center time in OF. (C) Center entries in OF. (D) Number of standing in OF. (E) Open arm time in EPM. (F) Open arm entries in EPM.

In the OF test, the total distance, the center time and the number of center entries were significantly lower in the MC group compared to the BC group, with the differences being statistically significant (*p* < 0.01). The CA‐H group exhibited significantly lower total distance, center time, and center entries than the MC group, and these differences were statistically significant (Total distance: *p* < 0.01, Center time: *p* < 0.05, Center entries: *p* < 0.05) (Figure 5A,B,C). However, the CA‐L and CA‐M groups were not statistically different from the MC group. The number of standing in the open field was significantly lower in the MC group compared to the BC group, and this difference was statistically significant (*p* < 0.001). The CA‐M and CA‐H groups exhibited a significant increase in the number of standing compared to the MC group, and the difference was statistically significant (CA‐M: *p* < 0.05, CA‐H: *p* < 0.01) (Figure 5D).

In the EPM test, the open arm time and open arm entries were both significantly lower in the MC group compared to the BC group, with a statistically significant difference (*p* < 0.001). These two indices were significantly increased in both the CA‐M and CA‐H groups compared with the MC group, and the differences were statistically significant (CA‐M: *p* < 0.01, CA‐H: *p* < 0.001) (Figure 5E,F). There was no statistical difference between the CA‐L group and the MC group.

These results suggest that CA can improve anxiety and depression‐like behaviors in IBS rats within the open field and EPM tests.

### 3.8 CA Reduces *MAOB* Levels in IBS Rats (*n* = 10)

The level of *MAOB* in the MC group was significantly higher than that in the BC group (*p* < 0.01). After two weeks of CA intervention, there were no significant differences in *MAOB* levels between the CA‐L or the CA‐M groups and the MC group. However, *MAOB* levels in the CA‐H group were significantly lower than those in the MC group (*p* < 0.05, Figure 1A). The results indicated that the level of *MAOB* was significantly increased in IBS model rats, which was significantly reduced by CA intervention, confirming the findings of network pharmacology and molecular docking.

### 3.9 CA Reduces TNF‐α, IL‐6 and IL‐1β Levels in IBS Rats (*n* = 10)

The level of TNF‐α was significantly elevated in the MC group compared to the BC group (*p* < 0.001). No significant differences were observed between the CA‐L or CA‐M groups and the MC group, whereas the CA‐H group showed a significant reduction in TNF‐α levels compared to the MC group (*p* < 0.001, Figure 1B).

IL‐6 levels were also significantly higher in the MC group than in the BC group (*p* < 0.001). No significant difference was found between the CA‐L and MC groups, while IL‐6 levels were significantly decreased in both the CA‐M and CA‐H groups compared with MC group (CA‐M: *p* < 0.01; CA‐H: *p* < 0.001, Figure 1C).

Similarly, IL‐1β levels were significantly increased in the MC group compared to the BC group (*p* < 0.001). Following CA intervention, IL‐1β levels were significantly reduced in the CA‐M and CA‐H groups compared to the MC group (*p* < 0.001), while no significant difference was observed between the CA‐L and MC groups (Figure 1D). Collectively, these results suggest that TNF‐α, IL‐6, and IL‐1β levels are elevated in IBS model rats and that CA, particularly at medium and high doses, effectively reduces these pro‐inflammatory cytokines.

## 4 Discussion

IBS is widely recognized as a functional gastrointestinal disorder that is closely linked to intestinal inflammation and disruptions in the microbiota–gut–brain axis, among other factors [33]. As the main active ingredient from the traditional Chinese herb cinnamon, CA has been shown to have a wide range of biological activities through modern pharmacological studies, especially in anti‐inflammatory and intestinal microbial regulation. For instance, CA can inhibit the cytokine storm induced by the SARS‐CoV‐2 ORF3 protein by scavenging reactive oxygen species in activated T cells, making it a potential candidate for COVID‐19 treatment [34]. Additionally, CA has been reported to reduce inflammatory cytokines, blood lipids, and atherosclerotic plaque area, as well as down‐regulate matrix metalloproteinase‐2 (MMP‐2) expression and NF‐κB phosphorylation in ApoE−/− mice, thereby exerting anti‐atherosclerotic effects [35]. In a mouse model of ulcerative colitis, CA significantly reduces intestinal inflammatory damage by lowering IL‐6 levels and inhibiting the expression of receptors for advanced glycation end products (RAGE), NF‐κB, and TNF‐α [36]. Moreover, compounds such as carvacrol, CA, and eugenol may offer novel approaches to circumvent the side effects associated with high antibiotic use, potentially contributing to the fight against antibiotic resistance [12]. These compounds have also been shown to reverse polymyxin resistance in *Enterobacteriaceae* and *A. baumannii* [37].

Given CA's anti‐inflammatory properties, we hypothesized that it might be effective in treating IBS through modulation of inflammatory pathways and related mechanisms. However, because of limited existing research, we employed network pharmacology and animal models to explore and validate CA's therapeutic potential in IBS.

Using a network pharmacology approach, we identified 139 potential targets of CA that may influence IBS. From the constructed PPI network, we identified 11 key targets most strongly associated with both CA and IBS, the majority of which are involved in inflammatory processes. Notably, NF‐κB subunit 1 (NFKB1) exhibited the highest degree value in the PPI network. NFKB1, a core member of the NF‐κB family, plays a pivotal role in regulating inflammation. Variants in NFKB1 are associated with hyperinflammatory states; truncations of NFKB1 have been shown to enhance inflammasome activation and type I interferon responses while inhibiting autophagy, leading to the accumulation of nucleotide‐binding oligomerization domain, leucine‐rich repeat‐containing protein 3 (NLRP3), and Toll/IL‐1 receptor domain‐containing adapter (TRIF) [38]. Other critical targets include janus kinase 2 (*JAK2*), a key non‐receptor tyrosine kinase that the JAK/STAT pathway linked to inflammation in various diseases [39, 40], and histone deacetylase 1 (*HDAC1*), which modulates gene transcription by tightening chromatin structure and plays roles in both pro‐ and anti‐inflammatory pathways [41, 42].

Among these targets, *MAOB* exhibited the strongest binding energy with CA in our molecular docking analysis, suggesting that CA may exert therapeutic effects on IBS by modulating *MAOB*‐related pathways. *MAOB*, one of two isoforms of the monoamine oxidase enzyme family located in mitochondria, degrades both biogenic and dietary monoamines [43]. Recent studies suggest that *MAOB* is a crucial enzyme in gamma‐aminobutyric acid synthesis [44]. It also possesses proinflammatory properties due to the generation of ROS during monoamine metabolism, which is implicated in tumor progression and metastasis [45, 46]. To date, there has been little research on the role of *MAOB* in IBS. Based on our network pharmacology and molecular docking analyses, we performed preliminary validation of CA’s effect on IBS via *MAOB*‐related inflammatory pathways using an animal model. We successfully established an IBS visceral hypersensitivity model in rats through NMS combined with WAS, which mimicked the typical features of IBS, including slow weight gain and reduced food intake. IBS rats also exhibited a decrease in total distance, center time, center entries, and the number of standing in OF, as well as a reduction in time spent on and entries into the open arms of EPM, thus indicating anxiety and depression‐like behaviors. We observed significantly elevated levels of *MAOB* in the colonic tissues of IBS model rats compared to controls, along with increased levels of TNF‐α, IL‐6, and IL‐1β.

Previous studies suggest that NMS and WAS may activate the hypothalamic–pituitary–adrenal (HPA) axis, leading to increased astrocyte reactivity in the central nervous system [47]. Chronic stress activates the HPA axis, resulting in the secretion of corticotropin‐releasing hormone (CRH) from the hypothalamus, which stimulates the release of cortisol via adrenocorticotropic hormone (ACTH) from the pituitary. Elevated cortisol can activate the amygdala and other emotion‐related brain regions, inducing anxiety and depression‐like behaviors. Dysregulation of glucocorticoid receptors leads to prolonged cortisol exposure, which induces the release of inflammatory cytokines, such as IL‐6 and TNF‐α and increases ROS production. This cascade activates NF‐κB and JNK signaling, thereby upregulating *MAOB* expression and activity. The increased oxidative deamination by *MAOB* generates additional ROS, such as H_2_O_2_, which further promotes proinflammatory signaling and cytokine release [48, 49, 50, 51]. These inflammatory cytokines contribute to visceral hypersensitivity by activating peripheral nerve receptors and modulating ion channels and membrane potentials [52]. Inflammation also disrupts homeostatic and allostatic systems of appetite regulation governed by dopaminergic signaling in the hypothalamus and mesolimbic pathways [53], thereby affecting food intake and potentially leading to further weight gain. After two weeks of CA administration, *MAOB* levels in the colonic tissues of IBS rats were significantly reduced. Furthermore, levels of TNF‐α, IL‐6, and IL‐1β decreased markedly. Concurrently, food intake and weight gain improved, and the frequency of loose stools was notably reduced. The anxiety and depression‐like behaviors of rats in OF and EPM were also improved.

Our study confirmed the mechanism through which CA alleviates visceral hypersensitivity and ameliorates the associated anxiety–depression‐like behaviors in IBS by regulating *MAOB*‐related signaling pathways. To our knowledge, this is the first study to investigate the therapeutic potential of CA in the treatment of IBS. However, several limitations should be acknowledged. First, although we identified the involvement of *MAOB*, we did not explore or validate upstream regulatory pathways, such as those related to the HPA axis. Second, *MAOB* may contribute to the pathophysiology of IBS not only through inflammatory signaling but also via neurotransmitter pathways, including the regulation of serotonin (5‐HT) and others [54]. Moreover, medium and low doses of CA exerted no significant influence on *MAOB* and other indicators. Given that the impact of CA on the aforementioned indicators is highly likely to be dose‐dependent, further pharmacodynamic investigations are required for verification to determine the optimal dose of CA. Furthermore, network pharmacology analysis identified additional potential targets of CA that warrant experimental validation. It is also likely that the therapeutic effects of CA on IBS result from the coordinated regulation of multiple molecular targets. Future studies may benefit from applying multi‐omics approaches to comprehensively assess the mechanisms by which CA intervenes in IBS, encompassing anti‐inflammatory effects, neurotransmitter modulation, and gut–brain axis regulation. Based on our findings and the traditional use of cinnamon—the herbal source of CA—in Chinese medicine [55], further clinical studies are warranted to evaluate the efficacy of CA in IBS patients. Such investigations could facilitate the development and transformation of CA into a novel therapeutic agent for IBS.

## 5 Conclusion

This study demonstrates that CA alleviates visceral hypersensitivity in IBS by targeting *MAOB* and pro‐inflammatory cytokines including TNF‐α, IL‐6, and IL‐1β, offering a new paradigm for mechanistic research on natural products. Moving forward, integrating multi‐omics technologies with clinical translational research will be essential for advancing CA as a candidate drug for personalized treatment strategies in IBS.

## Author Contributions

**Qingyang Yu:** conceptualization (lead), investigation (equal), and writing – original draft (lead). **Boqing Xu:** investigation (equal), data curation (lead), and formal analysis (lead). **Chunfang Dai:** methodology (lead), resources (equal), and validation (lead). **Yayan Pang:** supervision (equal), and project administration (lead). **Zhifang Dong:** writing – review and editing (lead), supervision (equal), funding acquisition (lead), and resources (equal).

## Ethics Statement

All experimental protocols were approved by the Ethics Committee of Children's Hospital of Chongqing Medical University (Approval Number: CHCMU‐IACUC20240508003), and every effort was made to minimize both the animal suffering and the number of animals used.

## Conflicts of Interest

Zhifang Dong is a member of editorial board of *Pediatric Discovery*, who was excluded from all dicision‐making related to the acceptance for publishing. Apart from this, the authors declare no conflicts of interest.

## Supporting information


Supporting Information S1

## Data Availability

The datasets utilized and/or scrutinized throughout the present study are accessible from the corresponding author upon a justified request.
