# Supplementary material for: Investigation of the Mechanism of Cinnamaldehyde in Irritable Bowel Syndrome Based via Network Pharmacology, Molecular Docking, and Animal Experiments
Source: Pediatr Discov. 2025 Oct 5;4(3):e70017. doi: 10.1002/pdi3.70017 (PMC13398650; doi:10.1002/pdi3.70017)
Supplement: Supplementary file 1 [file PDI3-4-e70017-s001.zip › Supplementary Materials/go kegg/mf/AnalysisReport.html]

## Metascape Gene List Analysis Report

metascape.org^1^

### Bar Graph Summary

Figure 1. Bar graph of enriched terms across input gene lists, colored by p-values.

Metascape only visualizes the top 20 clusters. Up to 100 enriched clusters can be viewed here.

### Gene Lists

User-provided gene identifiers are first converted into their corresponding H. sapiens Entrez gene IDs using the latest version of the database (last updated on 2025-01-01). If multiple identifiers correspond to the same Entrez gene ID, they will be considered as a single Entrez gene ID in downstream analyses. The gene lists are summarized in Table 1.

Table 1. Statistics of input gene lists.

| Name | Total | Unique |
| --- | --- | --- |
| MyList | 139 | 139 |

### Pathway and Process Enrichment Analysis

For each given gene list, pathway and process enrichment analysis have been carried out with the following ontology sources: GO Molecular Functions. All genes in the genome have been used as the enrichment background. Terms with a p-value < 0.01, a minimum count of 3, and an enrichment factor > 1.5 (the enrichment factor is the ratio between the observed counts and the counts expected by chance) are collected and grouped into clusters based on their membership similarities. More specifically, p-values are calculated based on the cumulative hypergeometric distribution^2^, and q-values are calculated using the Benjamini-Hochberg procedure to account for multiple testings^3^. Kappa scores^4^ are used as the similarity metric when performing hierarchical clustering on the enriched terms, and sub-trees with a similarity of > 0.3 are considered a cluster. The most statistically significant term within a cluster is chosen to represent the cluster.

Table 2. Top 20 clusters with their representative enriched terms (one per cluster). "Count" is the number of genes in the user-provided lists with membership in the given ontology term. "%" is the percentage of all of the user-provided genes that are found in the given ontology term (only input genes with at least one ontology term annotation are included in the calculation). "Log10(P)" is the p-value in log base 10. "Log10(q)" is the multi-test adjusted p-value in log base 10.

| GO | Category | Description | Count | % | Log10(P) | Log10(q) |
| --- | --- | --- | --- | --- | --- | --- |
| GO:0016491 | GO Molecular Functions | oxidoreductase activity | 27 | 19.42 | -16.34 | -12.64 |
| GO:0015464 | GO Molecular Functions | acetylcholine receptor activity | 9 | 6.47 | -15.95 | -12.55 |
| GO:0004089 | GO Molecular Functions | carbonate dehydratase activity | 8 | 5.76 | -15.00 | -11.90 |
| GO:0031078 | GO Molecular Functions | histone H3K14 deacetylase activity, hydrolytic mechanism | 7 | 5.04 | -14.36 | -11.61 |
| GO:0140993 | GO Molecular Functions | histone modifying activity | 22 | 15.83 | -12.36 | -9.93 |
| GO:0019900 | GO Molecular Functions | kinase binding | 22 | 15.83 | -11.01 | -8.64 |
| GO:0016907 | GO Molecular Functions | G protein-coupled acetylcholine receptor activity | 4 | 2.88 | -8.67 | -6.55 |
| GO:0016788 | GO Molecular Functions | hydrolase activity, acting on ester bonds | 18 | 12.95 | -7.85 | -5.78 |
| GO:0020037 | GO Molecular Functions | heme binding | 9 | 6.47 | -7.68 | -5.62 |
| GO:0004032 | GO Molecular Functions | aldose reductase (NADPH) activity | 4 | 2.88 | -6.69 | -4.76 |
| GO:0042803 | GO Molecular Functions | protein homodimerization activity | 16 | 11.51 | -6.58 | -4.67 |
| GO:0004175 | GO Molecular Functions | endopeptidase activity | 12 | 8.63 | -6.39 | -4.50 |
| GO:0030058 | GO Molecular Functions | aliphatic amine dehydrogenase activity | 3 | 2.16 | -6.03 | -4.17 |
| GO:0008324 | GO Molecular Functions | monoatomic cation transmembrane transporter activity | 14 | 10.07 | -5.71 | -3.87 |
| GO:0044389 | GO Molecular Functions | ubiquitin-like protein ligase binding | 10 | 7.19 | -5.59 | -3.77 |
| GO:0043236 | GO Molecular Functions | laminin binding | 4 | 2.88 | -5.03 | -3.26 |
| GO:0016229 | GO Molecular Functions | steroid dehydrogenase activity | 4 | 2.88 | -4.80 | -3.06 |
| GO:0033218 | GO Molecular Functions | amide binding | 8 | 5.76 | -4.71 | -2.98 |
| GO:0048156 | GO Molecular Functions | tau protein binding | 4 | 2.88 | -4.34 | -2.66 |
| GO:0001046 | GO Molecular Functions | core promoter sequence-specific DNA binding | 4 | 2.88 | -4.30 | -2.63 |

To further capture the relationships between the terms, a subset of enriched terms has been selected and rendered as a network plot, where terms with a similarity > 0.3 are connected by edges. We select the terms with the best p-values from each of the 20 clusters, with the constraint that there are no more than 15 terms per cluster and no more than 250 terms in total. The network is visualized using Cytoscape^5^, where each node represents an enriched term and is colored first by its cluster ID (Figure 2.a) and then by its p-value (Figure 2.b). These networks can be interactively viewed in Cytoscape through the .cys files (contained in the Zip package, which also contains a publication-quality version as a PDF) or within a browser by clicking on the web icon. For clarity, term labels are only shown for one term per cluster, so it is recommended to use Cytoscape or a browser to visualize the network in order to inspect all node labels. We can also export the network into a PDF file within Cytoscape, and then edit the labels using Adobe Illustrator for publication purposes. To switch off all labels, delete the "Label" mapping under the "Style" tab within Cytoscape, and then export the network view.

Figure 2. Network of enriched terms: (a) colored by cluster ID, where nodes that share the same cluster ID are typically close to each other; (b) colored by p-value, where terms containing more genes tend to have a more significant p-value.

|  |  |
| --- | --- |
|  |  |
|  |  |

### Protein-protein Interaction Enrichment Analysis

For each given gene list, protein-protein interaction enrichment analysis has been carried out with the following databases: STRING^6^, BioGrid^7^, OmniPath^8^, InWeb\_IM^9^.Only physical interactions in STRING (physical score > 0.132) and BioGrid are used (details). The resultant network contains the subset of proteins that form physical interactions with at least one other member in the list. If the network contains between 3 and 500 proteins, the Molecular Complex Detection (MCODE) algorithm^10^ has been applied to identify densely connected network components. The MCODE networks identified for individual gene lists have been gathered and are shown in Figure 3.

Pathway and process enrichment analysis has been applied to each MCODE component independently, and the three best-scoring terms by p-value have been retained as the functional description of the corresponding components, shown in the tables underneath corresponding network plots within Figure 3.

Figure 3. Protein-protein interaction network and MCODE components identified in the gene lists.

| GO | Description | Log10(P) |
| --- | --- | --- |
| GO:0015464 | acetylcholine receptor activity | -16.4 |
| GO:0016491 | oxidoreductase activity | -15.5 |
| GO:0004089 | carbonate dehydratase activity | -15.4 |

| Color | MCODE | GO | Description | Log10(P) |
| --- | --- | --- | --- | --- |
|  | MCODE\_1 | GO:0016491 | oxidoreductase activity | -14.6 |
|  | MCODE\_1 | GO:0004745 | all-trans-retinol dehydrogenase (NAD+) activity | -14.0 |
|  | MCODE\_1 | GO:0004022 | alcohol dehydrogenase (NAD+) activity | -13.7 |
|  | MCODE\_2 | GO:0022848 | acetylcholine-gated monoatomic cation-selective channel activity | -14.6 |
|  | MCODE\_2 | GO:0015464 | acetylcholine receptor activity | -14.0 |
|  | MCODE\_2 | GO:0005231 | excitatory extracellular ligand-gated monoatomic ion channel activity | -12.7 |
|  | MCODE\_3 | GO:0008227 | G protein-coupled amine receptor activity | -7.3 |
|  | MCODE\_3 | GO:0030594 | neurotransmitter receptor activity | -6.2 |
|  | MCODE\_4 | GO:0042826 | histone deacetylase binding | -11.1 |
|  | MCODE\_4 | GO:0003682 | chromatin binding | -10.2 |
|  | MCODE\_4 | GO:0034739 | histone H4K16 deacetylase activity, hydrolytic mechanism | -9.3 |
|  | MCODE\_5 | GO:0030594 | neurotransmitter receptor activity | -10.0 |
|  | MCODE\_5 | GO:0008227 | G protein-coupled amine receptor activity | -8.0 |
|  | MCODE\_5 | GO:0098960 | postsynaptic neurotransmitter receptor activity | -7.3 |
|  | MCODE\_6 | GO:0004089 | carbonate dehydratase activity | -13.4 |
|  | MCODE\_6 | GO:0016836 | hydro-lyase activity | -10.8 |
|  | MCODE\_6 | GO:0016835 | carbon-oxygen lyase activity | -10.4 |
|  | MCODE\_8 | GO:0004089 | carbonate dehydratase activity | -10.0 |
|  | MCODE\_8 | GO:0016836 | hydro-lyase activity | -8.1 |
|  | MCODE\_8 | GO:0016835 | carbon-oxygen lyase activity | -7.8 |

### Reference

1. Zhou et al., Metascape provides a biologist-oriented resource for the analysis of systems-level datasets. Nature Communications (2019) 10(1):1523.
2. Zar, J.H. Biostatistical Analysis 1999 4th edn., NJ Prentice Hall, pp. 523
3. Hochberg Y., Benjamini Y. More powerful procedures for multiple significance testing. Statistics in Medicine (1990) 9:811-818.
4. Cohen, J. A coefficient of agreement for nominal scales. Educ. Psychol. Meas. (1960) 20:27-46.
5. Shannon P. et al., Cytoscape: a software environment for integrated models of biomolecular interaction networks. Genome Res (2003) 11:2498-2504.
6. Szklarczyk D. et al. STRING v11: protein-protein association networks with increased coverage, supporting functional discovery in genome-wide experimental datasets. Nucleic Acids Res. (2019) 47:D607-613.
7. Stark C. et al. BioGRID: a general repository for interaction datasets. Nucleic Acids Res. (2006) 34:D535-539.
8. Turei D. et al. A scored human protein-protein interaction network to catalyze genomic interpretation. Nat. Methods. (2016) 13:966-967.
9. Li T. et al. A scored human protein-protein interaction network to catalyze genomic interpretation. Nat. Methods. (2017) 14:61-64.
10. Bader, G.D. et al. An automated method for finding molecular complexes in large protein interaction networks. BMC bioinformatics (2003) 4:2.
