# Supplementary material for: Investigation of the Mechanism of Cinnamaldehyde in Irritable Bowel Syndrome Based via Network Pharmacology, Molecular Docking, and Animal Experiments
Source: Pediatr Discov. 2025 Oct 5:e70017. Online ahead of print. doi: 10.1002/pdi3.70017 (PMC13398650; doi:10.1002/pdi3.70017)
Supplement: Supplementary file 1 — Supporting Information S1 [file PDI3-9999-0-s001.zip › Supplementary Materials/go kegg/bp/AnalysisReport.pptx]

## Slide 1
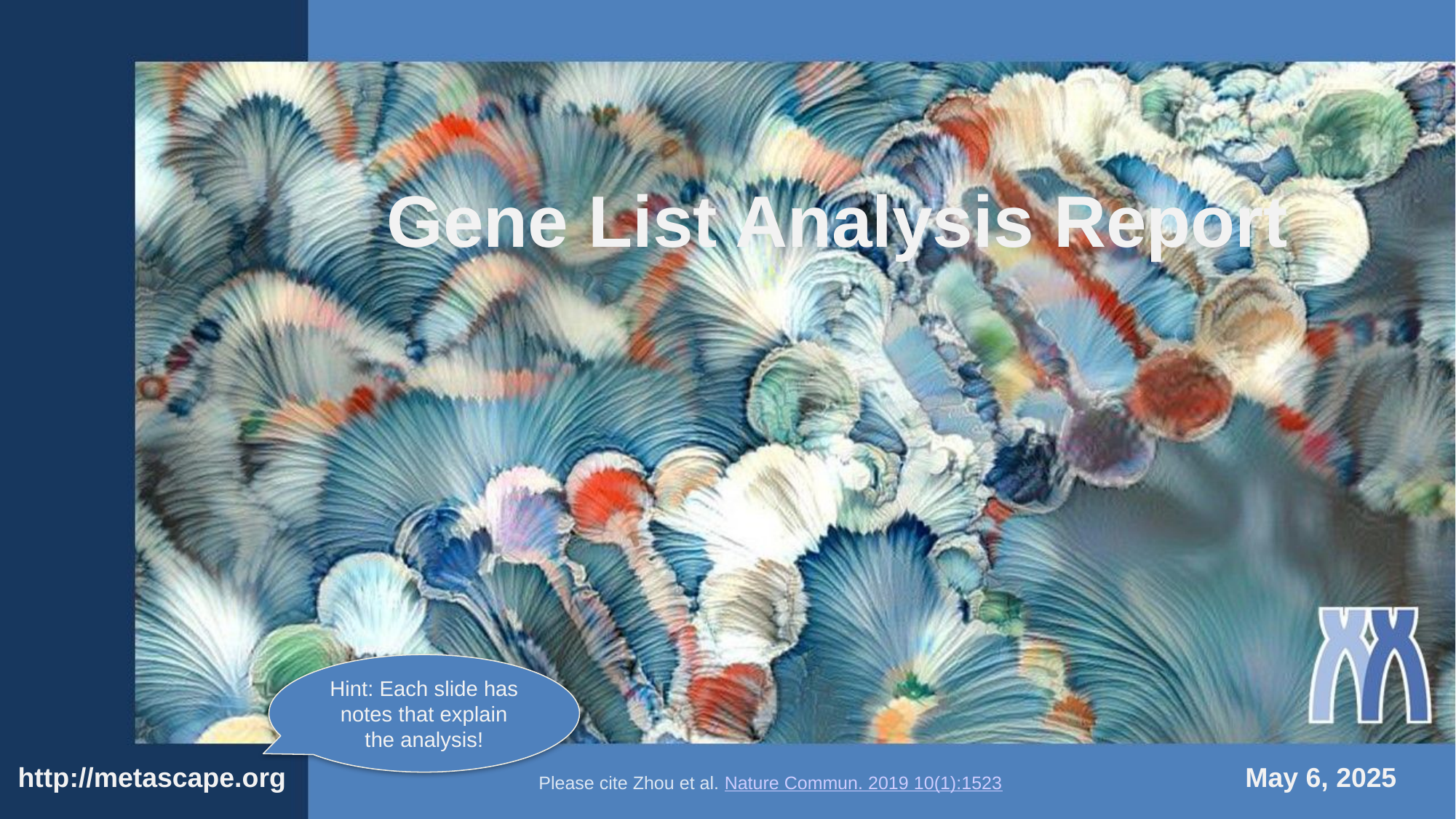

Gene List Analysis Report
Hint: Each slide has notes that explain the analysis!
http://metascape.org
May 6, 2025
Please cite Zhou et al. Nature Commun. 2019 10(1):1523

## Slide 2
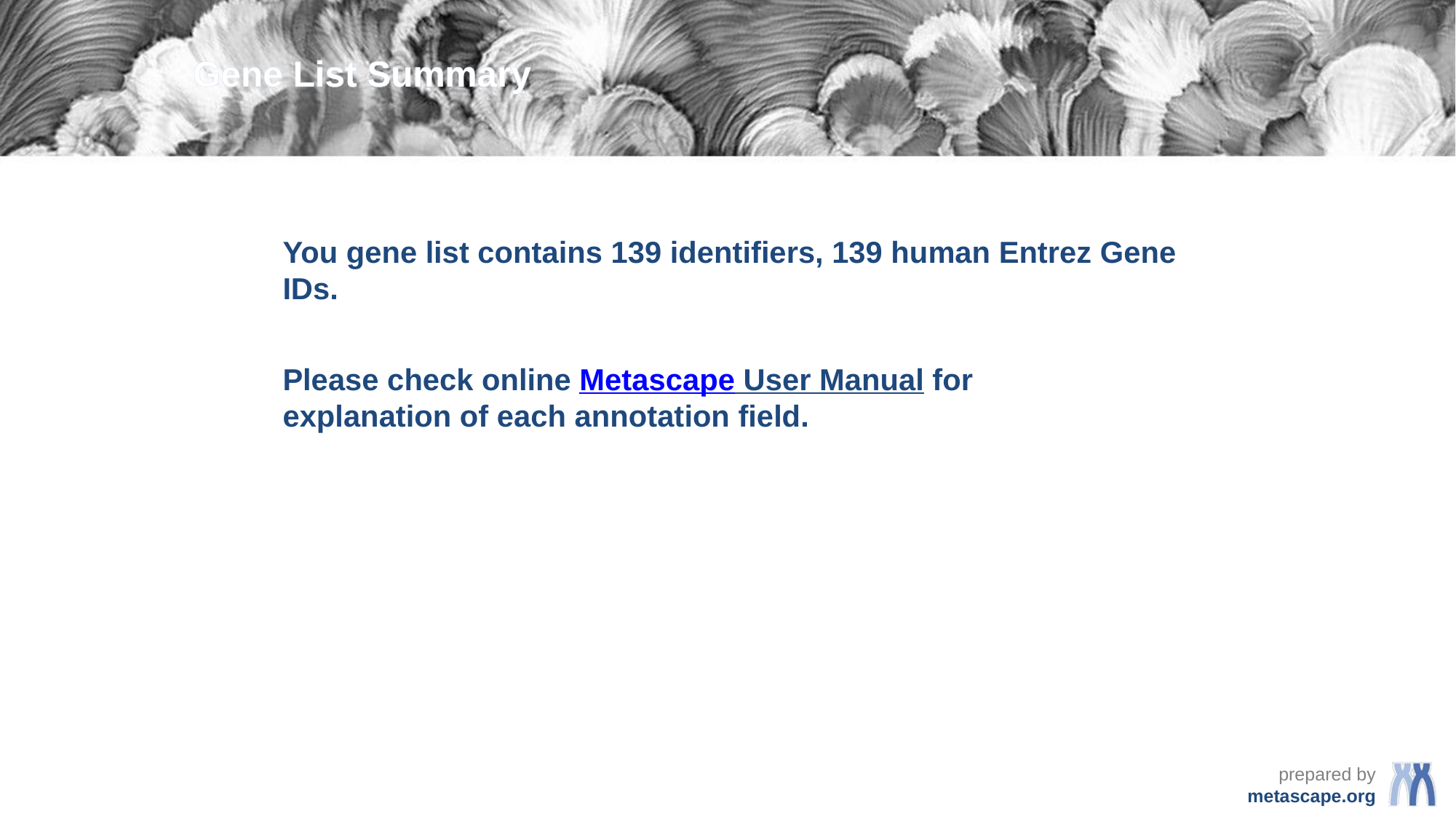

Gene List Summary
You gene list contains 139 identifiers, 139 human Entrez Gene IDs.
Please check online Metascape User Manual for explanation of each annotation field.

## Slide 3
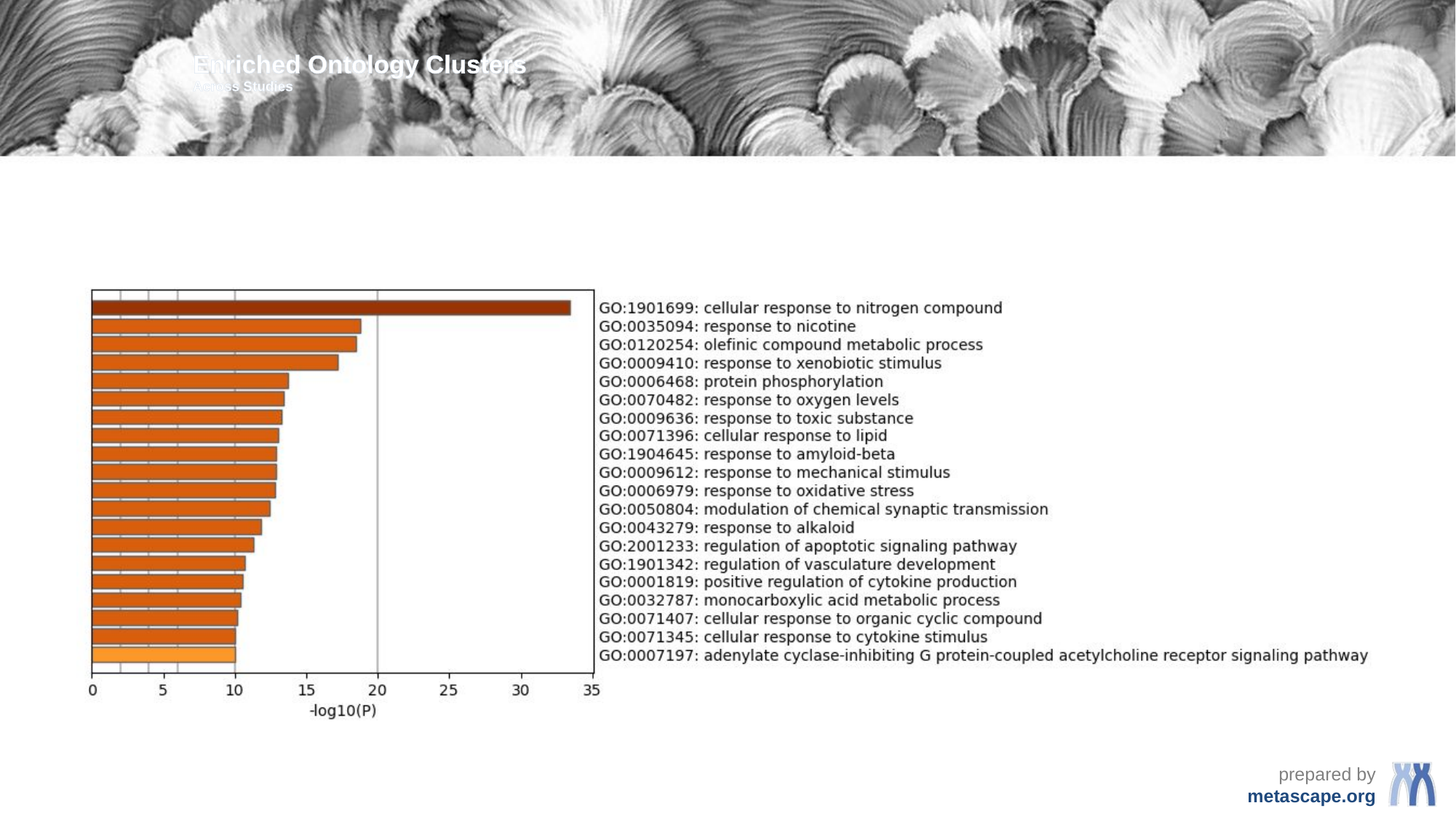

Enriched Ontology Clusters
Across Studies

## Slide 4
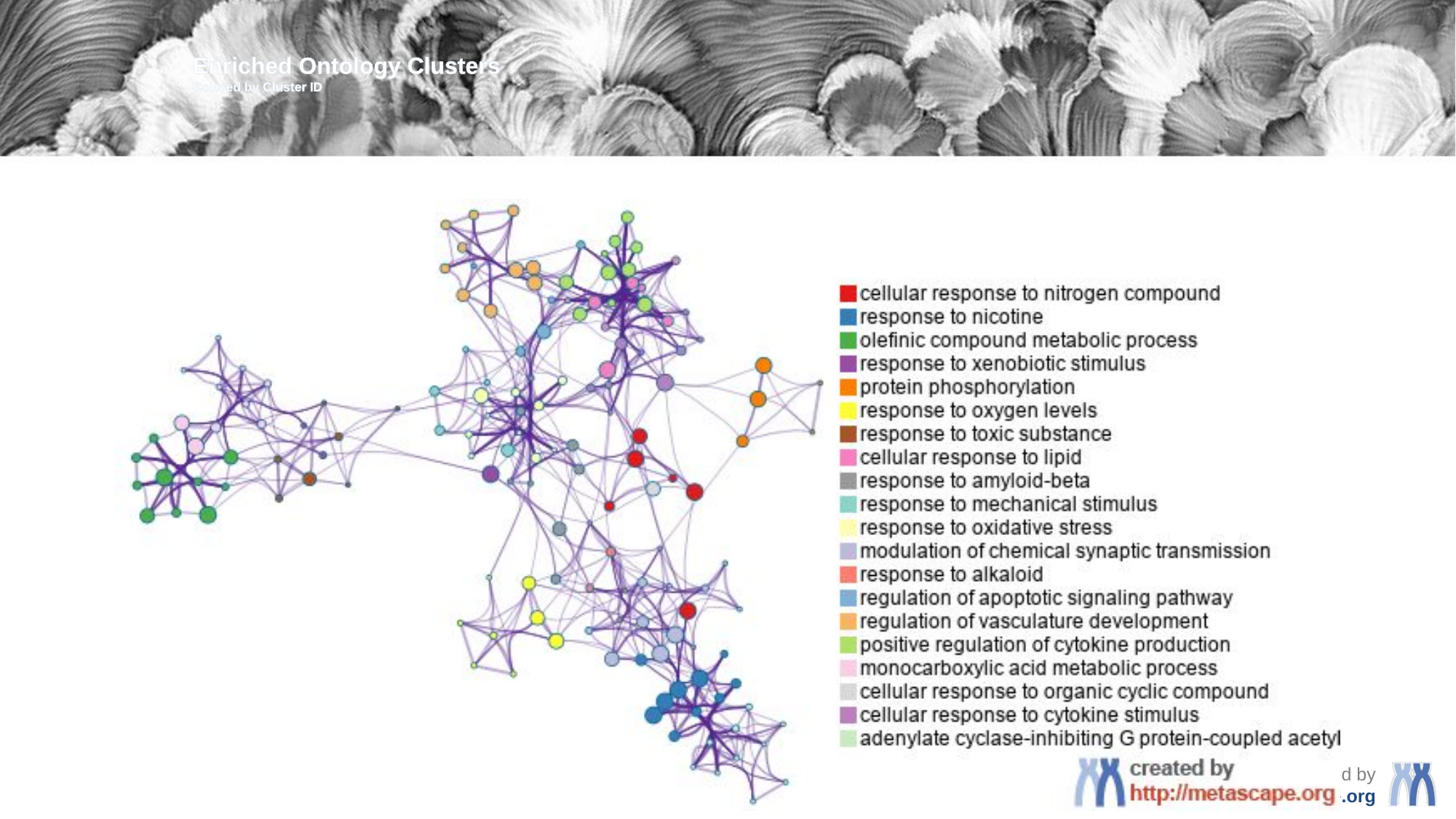

Enriched Ontology Clusters
Colored by Cluster ID

## Slide 5
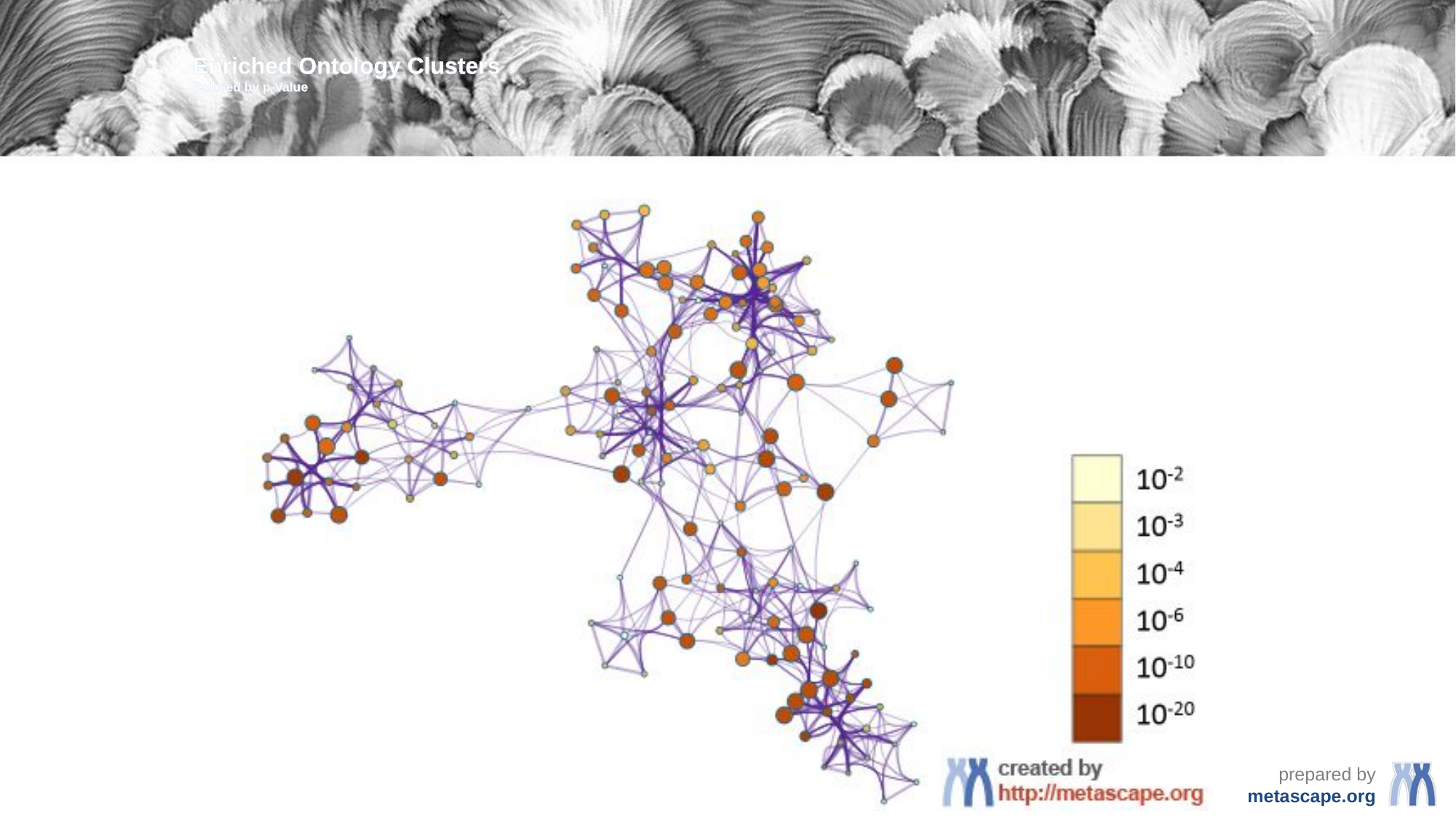

Enriched Ontology Clusters
Colored by p-Value

## Slide 6
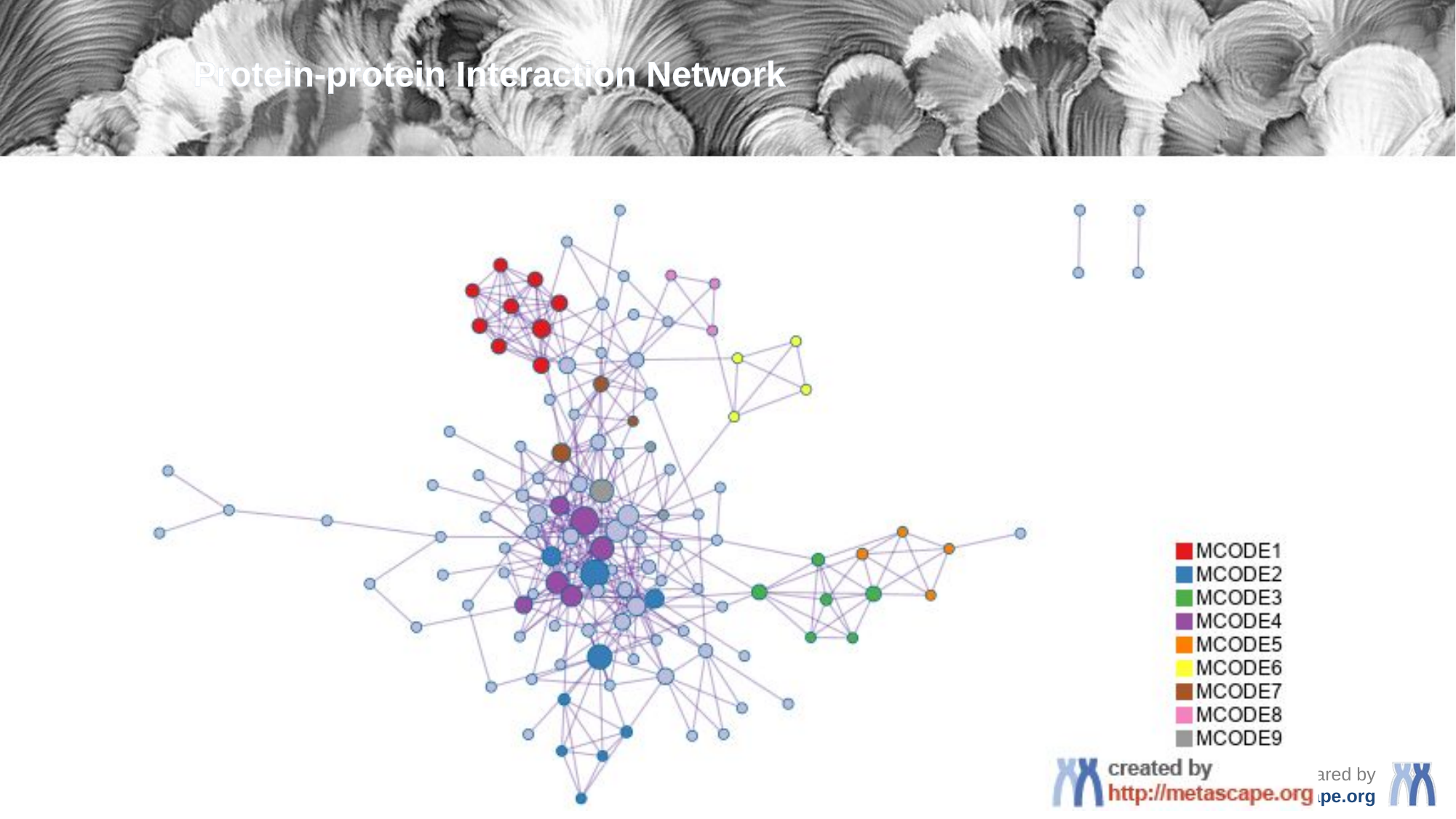

Protein-protein Interaction Network

## Slide 7
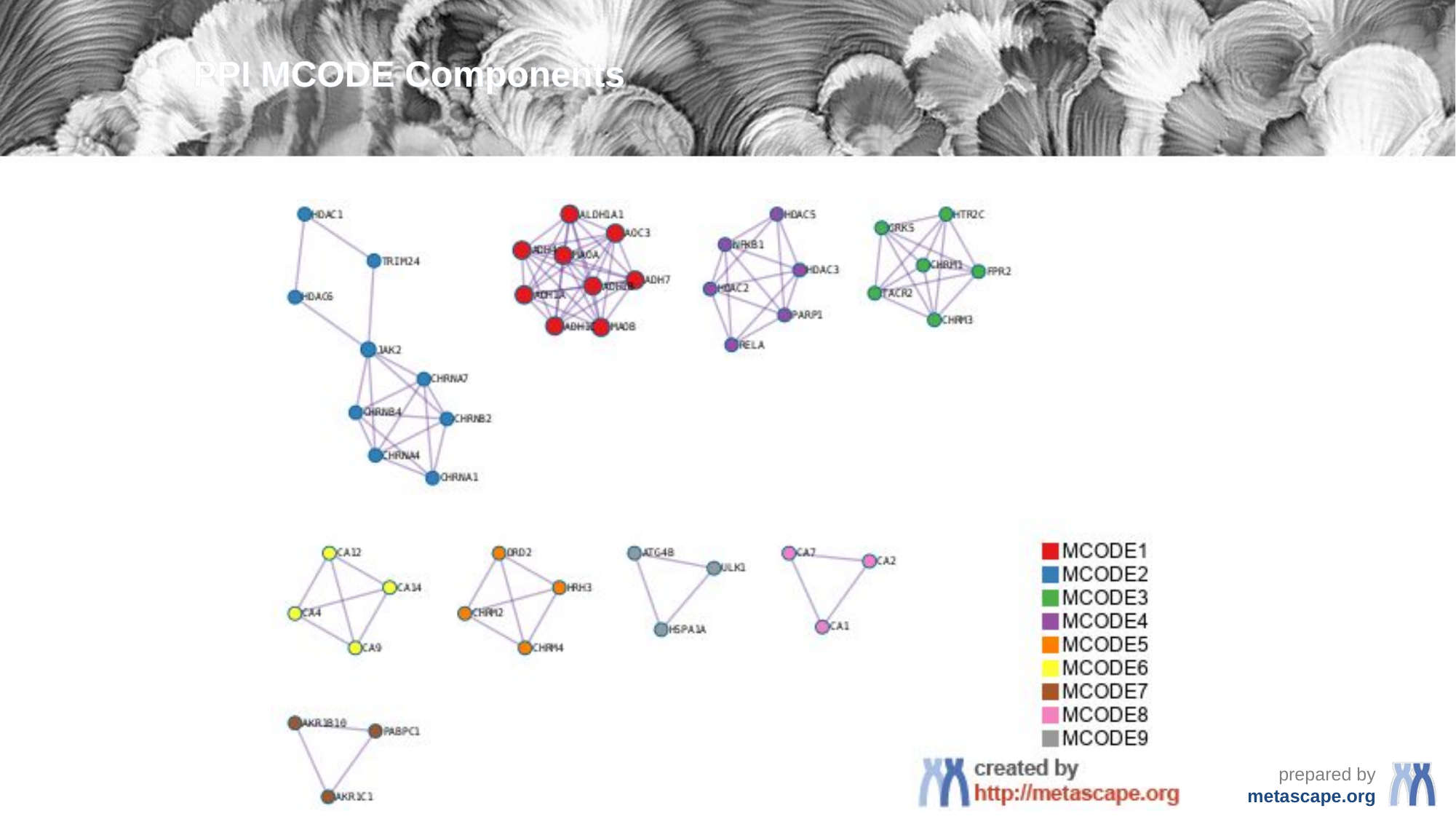

PPI MCODE Components

## Slide 8
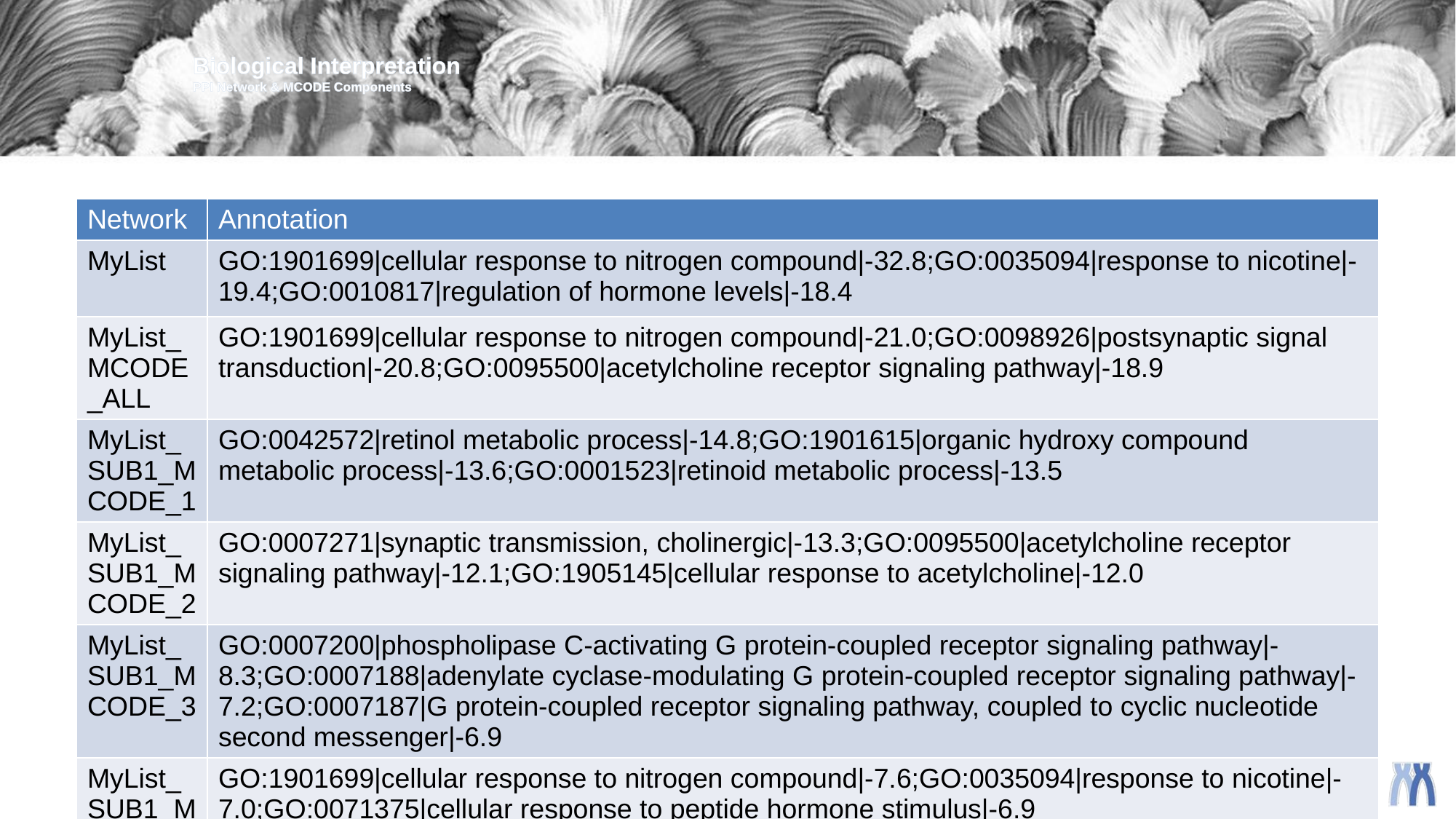

Biological Interpretation
PPI Network & MCODE Components
| Network | Annotation |
| --- | --- |
| MyList | GO:1901699|cellular response to nitrogen compound|-32.8;GO:0035094|response to nicotine|-19.4;GO:0010817|regulation of hormone levels|-18.4 |
| MyList\_MCODE\_ALL | GO:1901699|cellular response to nitrogen compound|-21.0;GO:0098926|postsynaptic signal transduction|-20.8;GO:0095500|acetylcholine receptor signaling pathway|-18.9 |
| MyList\_SUB1\_MCODE\_1 | GO:0042572|retinol metabolic process|-14.8;GO:1901615|organic hydroxy compound metabolic process|-13.6;GO:0001523|retinoid metabolic process|-13.5 |
| MyList\_SUB1\_MCODE\_2 | GO:0007271|synaptic transmission, cholinergic|-13.3;GO:0095500|acetylcholine receptor signaling pathway|-12.1;GO:1905145|cellular response to acetylcholine|-12.0 |
| MyList\_SUB1\_MCODE\_3 | GO:0007200|phospholipase C-activating G protein-coupled receptor signaling pathway|-8.3;GO:0007188|adenylate cyclase-modulating G protein-coupled receptor signaling pathway|-7.2;GO:0007187|G protein-coupled receptor signaling pathway, coupled to cyclic nucleotide second messenger|-6.9 |
| MyList\_SUB1\_MCODE\_4 | GO:1901699|cellular response to nitrogen compound|-7.6;GO:0035094|response to nicotine|-7.0;GO:0071375|cellular response to peptide hormone stimulus|-6.9 |
| MyList\_SUB1\_MCODE\_5 | GO:0007193|adenylate cyclase-inhibiting G protein-coupled receptor signaling pathway|-10.3;GO:0007197|adenylate cyclase-inhibiting G protein-coupled acetylcholine receptor signaling pathway|-10.3;GO:0007213|G protein-coupled acetylcholine receptor signaling pathway|-8.9 |

## Slide 9
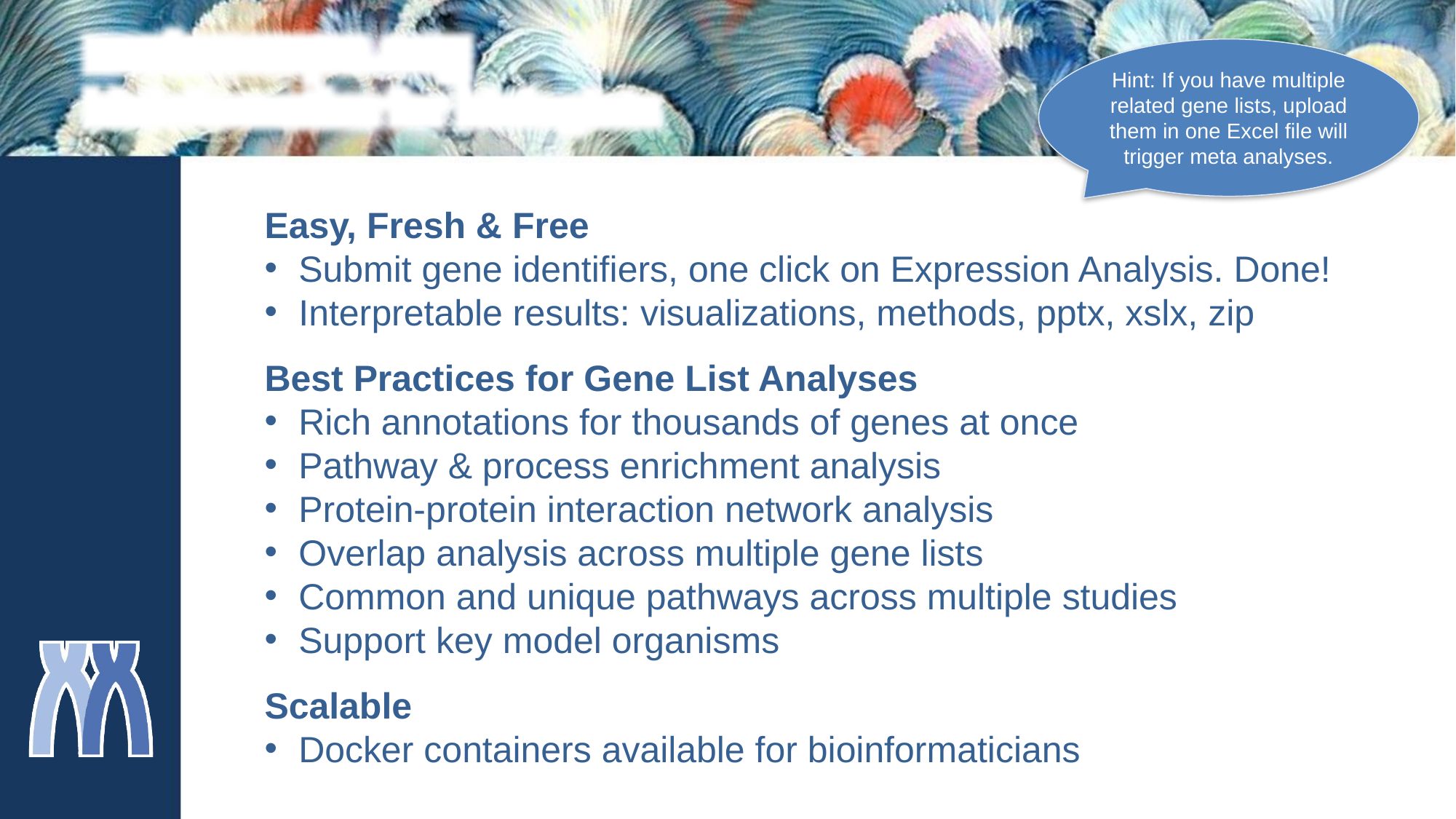

metascape.org
bioinformatics for biologists
Hint: If you have multiple related gene lists, upload them in one Excel file will trigger meta analyses.
Easy, Fresh & Free
Submit gene identifiers, one click on Expression Analysis. Done!
Interpretable results: visualizations, methods, pptx, xslx, zip
Best Practices for Gene List Analyses
Rich annotations for thousands of genes at once
Pathway & process enrichment analysis
Protein-protein interaction network analysis
Overlap analysis across multiple gene lists
Common and unique pathways across multiple studies
Support key model organisms
Scalable
Docker containers available for bioinformaticians
