# Supplementary material for: Investigation of the Mechanism of Cinnamaldehyde in Irritable Bowel Syndrome Based via Network Pharmacology, Molecular Docking, and Animal Experiments
Source: Pediatr Discov. 2025 Oct 5:e70017. Online ahead of print. doi: 10.1002/pdi3.70017 (PMC13398650; doi:10.1002/pdi3.70017)
Supplement: Supplementary file 1 — Supporting Information S1 [file PDI3-9999-0-s001.zip › Supplementary Materials/go kegg/bp/Enrichment_GO/ColorByCluster.pdf]

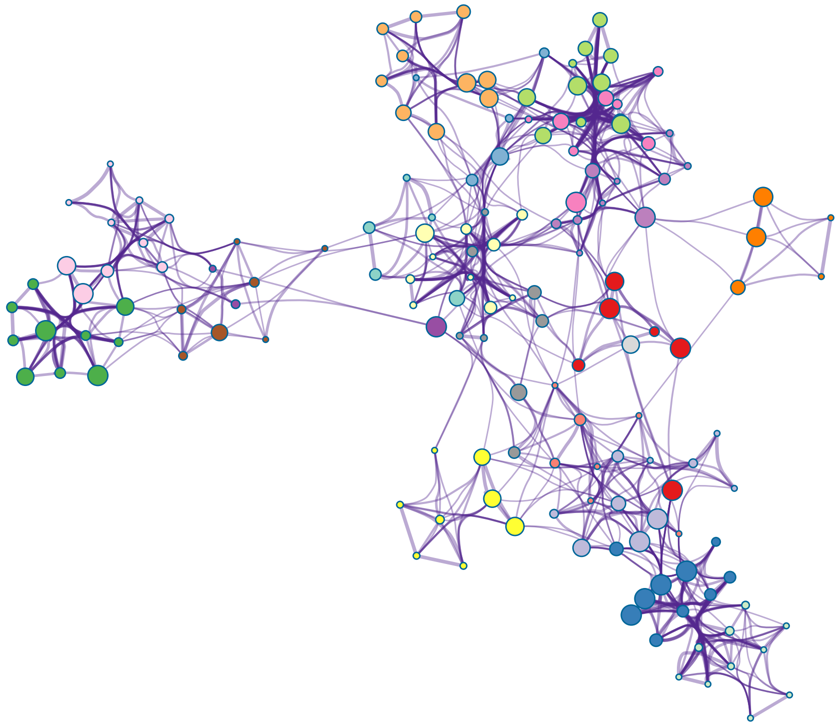

- cellular response to nitrogen compound
- response to nicotine
- olefinic compound metabolic process
- response to xenobiotic stimulus
- protein phosphorylation
- response to oxygen levels
- response to toxic substance
- cellular response to lipid
- response to amyloid-beta
- response to mechanical stimulus
- response to oxidative stress
- modulation of chemical synaptic transmission
- response to alkaloid
- regulation of apoptotic signaling pathway
- regulation of vasculature development
- positive regulation of cytokine production
- monocarboxylic acid metabolic process
- cellular response to organic cyclic compound
- cellular response to cytokine stimulus
- adenylate cyclase-inhibiting G protein-coupled acetyl

created by
http://metascape.org
