# Supplementary material for: Investigation of the Mechanism of Cinnamaldehyde in Irritable Bowel Syndrome Based via Network Pharmacology, Molecular Docking, and Animal Experiments
Source: Pediatr Discov. 2025 Oct 5:e70017. Online ahead of print. doi: 10.1002/pdi3.70017 (PMC13398650; doi:10.1002/pdi3.70017)
Supplement: Supplementary file 1 — Supporting Information S1 [file PDI3-9999-0-s001.zip › Supplementary Materials/go kegg/cc/AnalysisReport.pptx]

## Slide 1
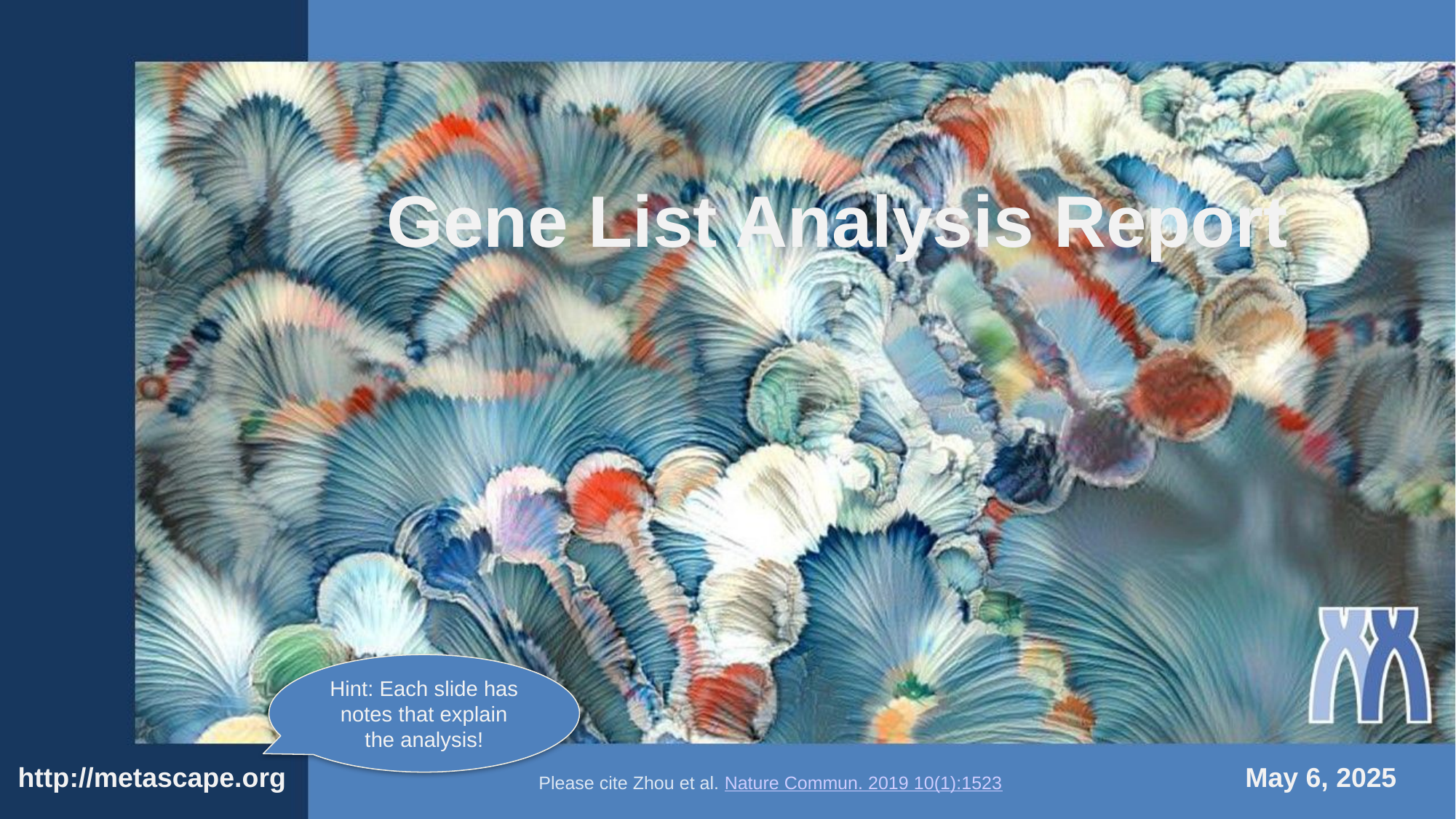

Gene List Analysis Report
Hint: Each slide has notes that explain the analysis!
http://metascape.org
May 6, 2025
Please cite Zhou et al. Nature Commun. 2019 10(1):1523

## Slide 2
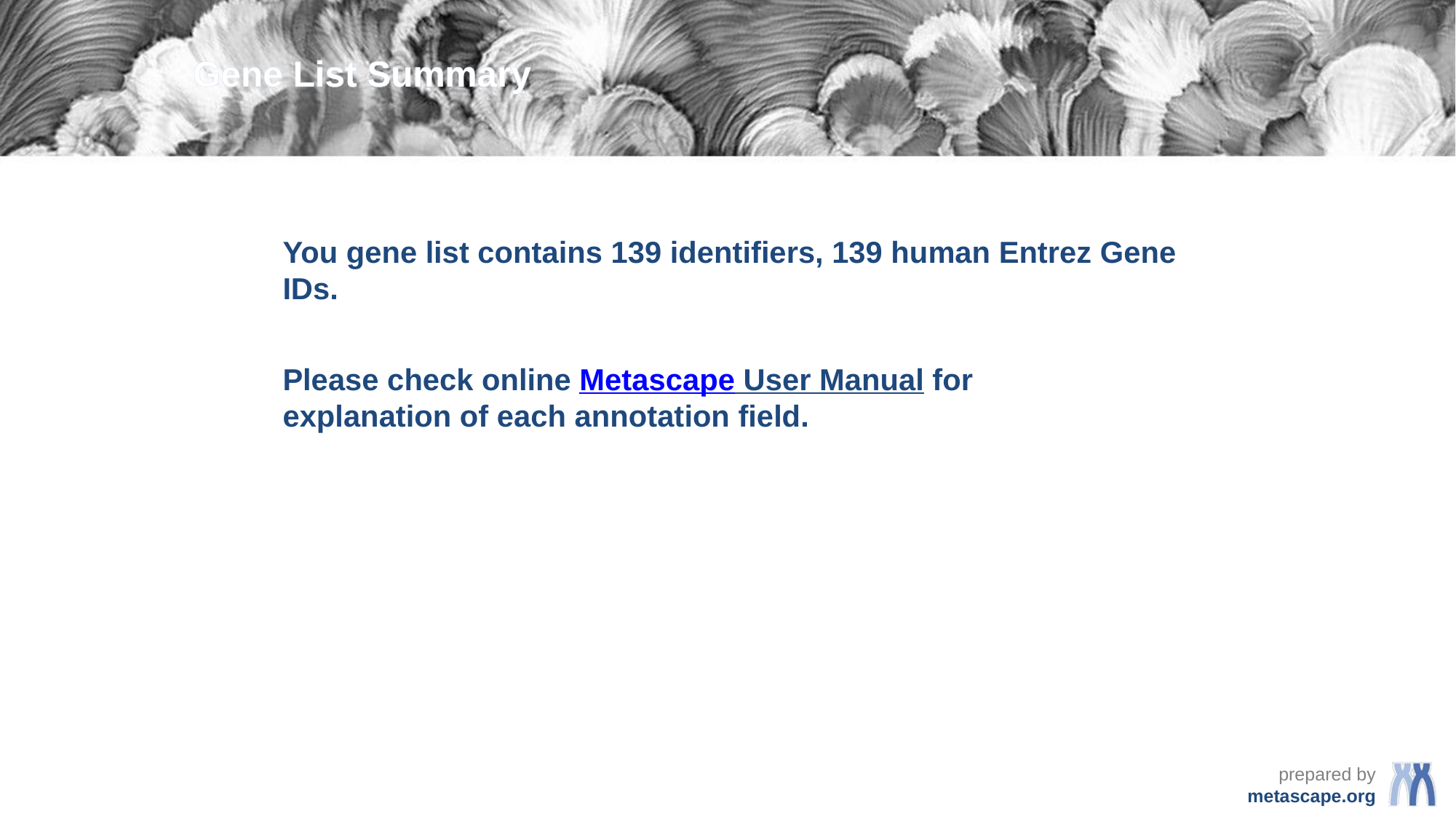

Gene List Summary
You gene list contains 139 identifiers, 139 human Entrez Gene IDs.
Please check online Metascape User Manual for explanation of each annotation field.

## Slide 3
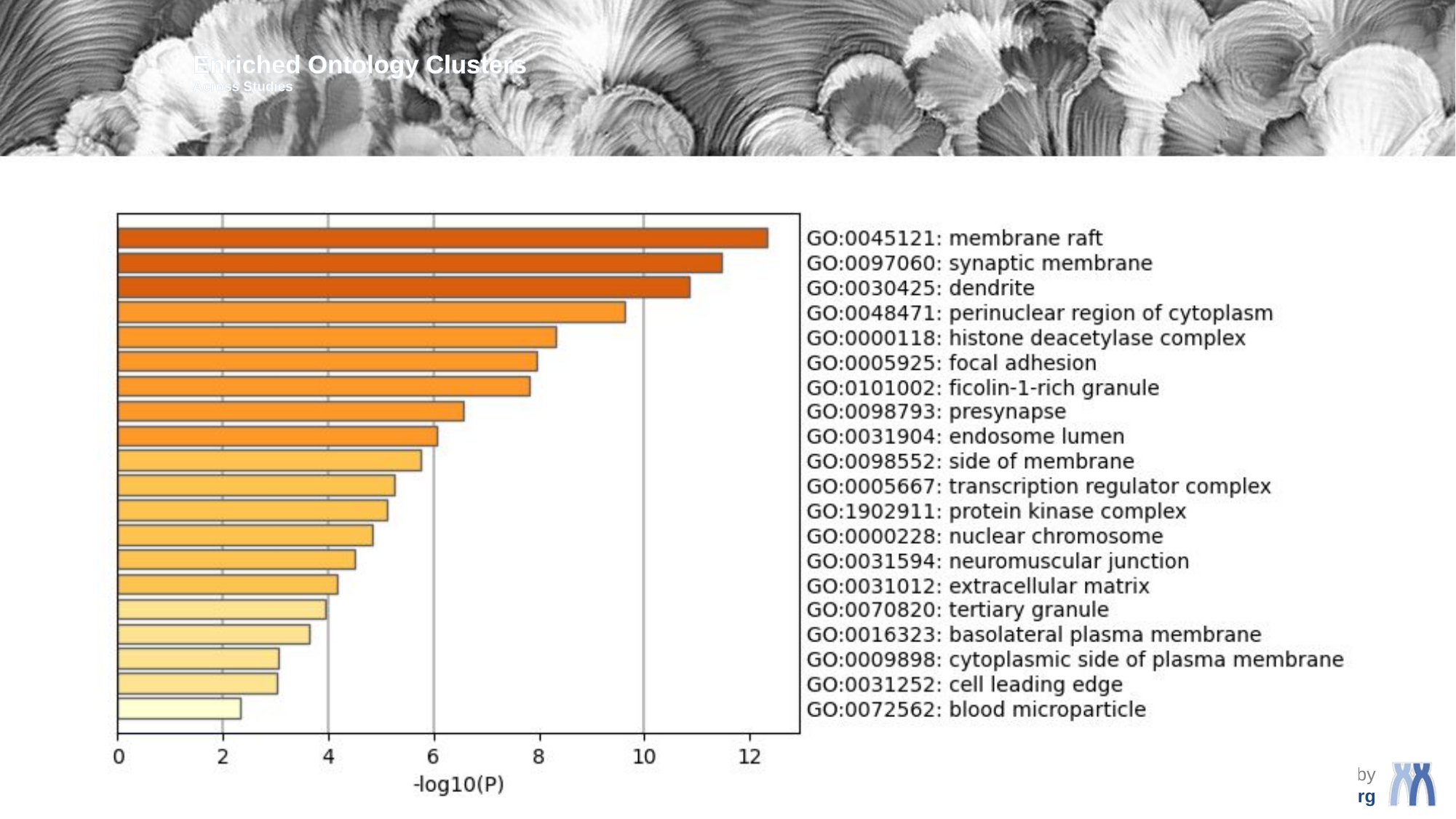

Enriched Ontology Clusters
Across Studies

## Slide 4
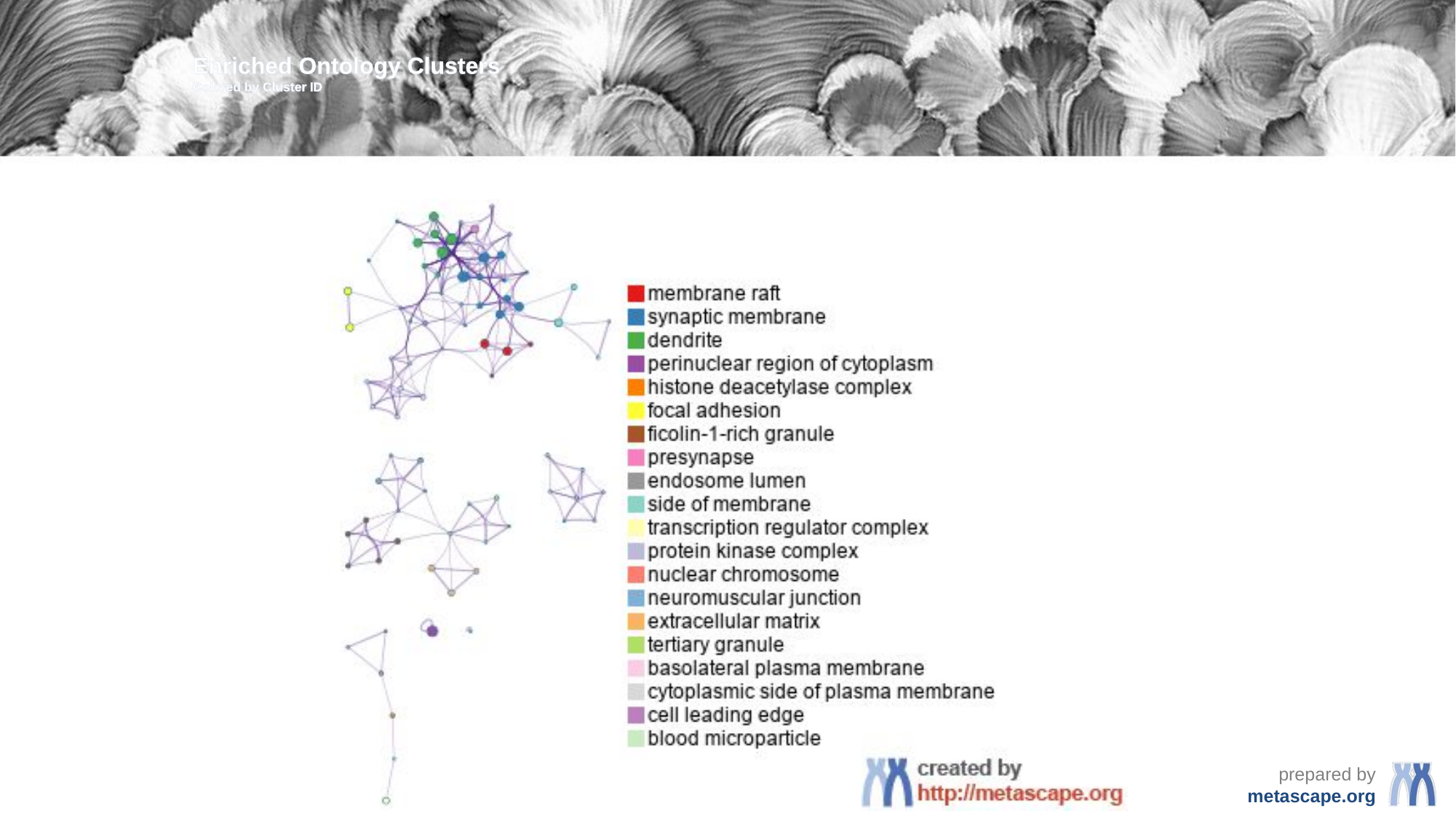

Enriched Ontology Clusters
Colored by Cluster ID

## Slide 5
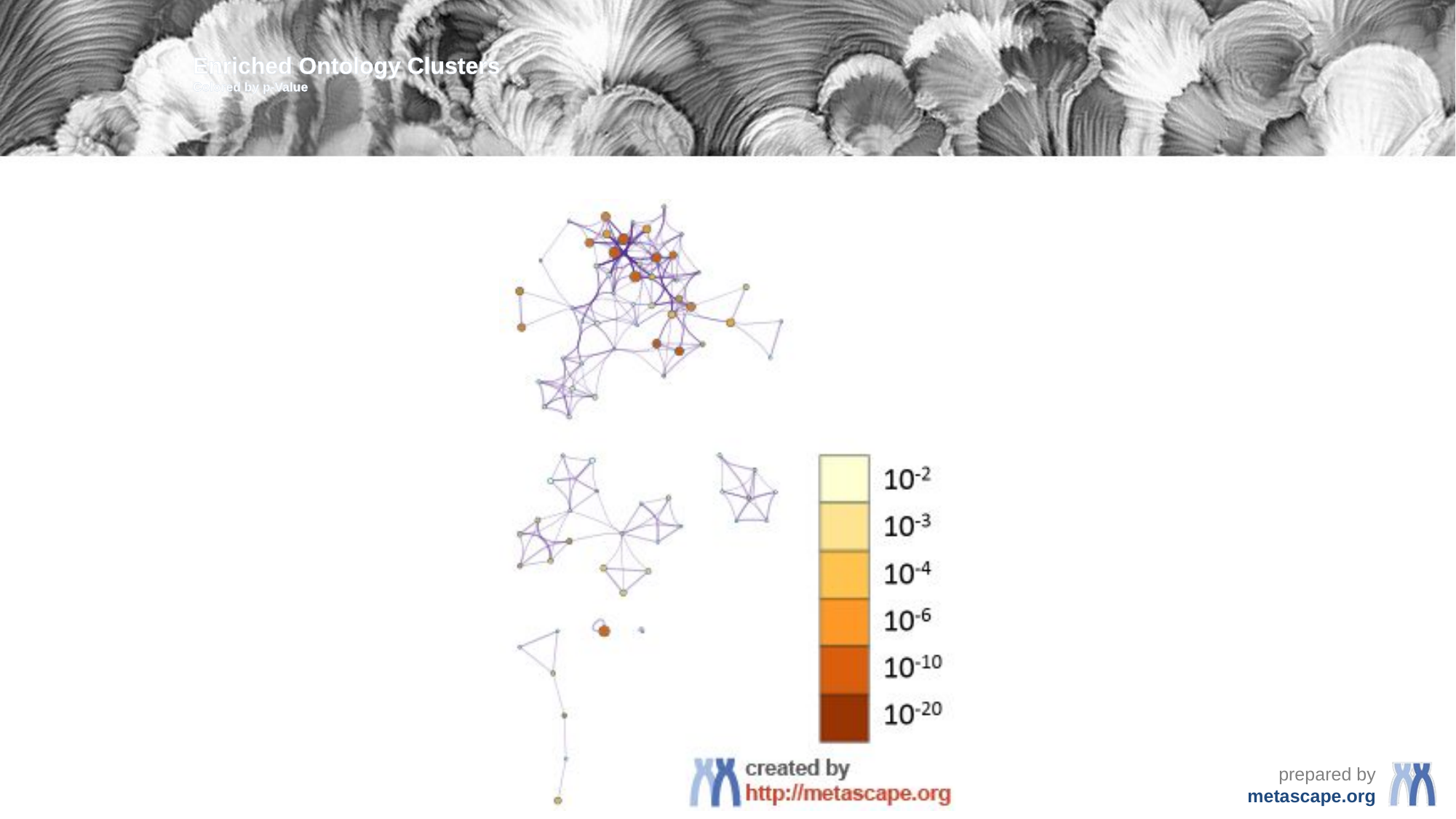

Enriched Ontology Clusters
Colored by p-Value

## Slide 6
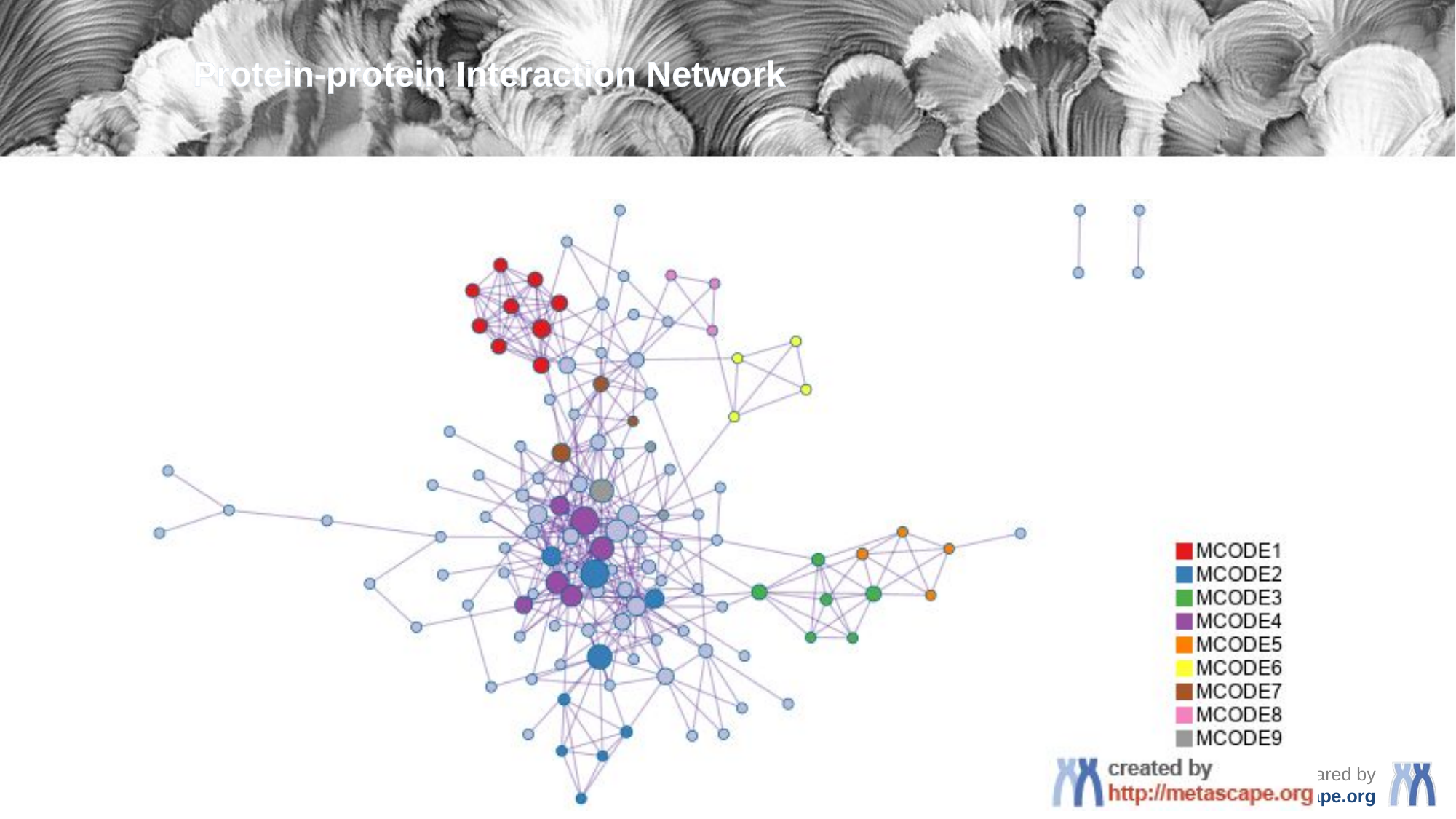

Protein-protein Interaction Network

## Slide 7
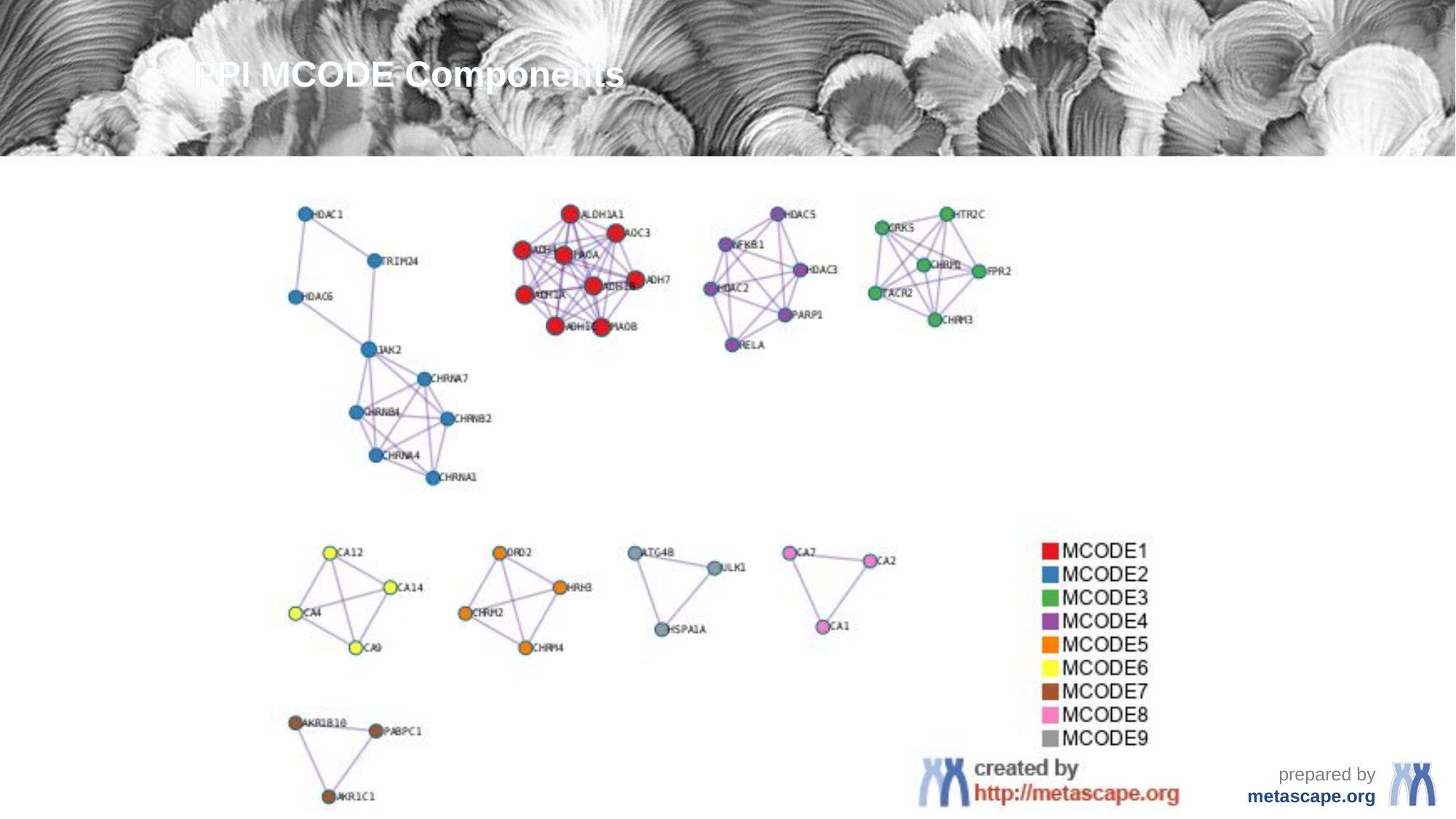

PPI MCODE Components

## Slide 8
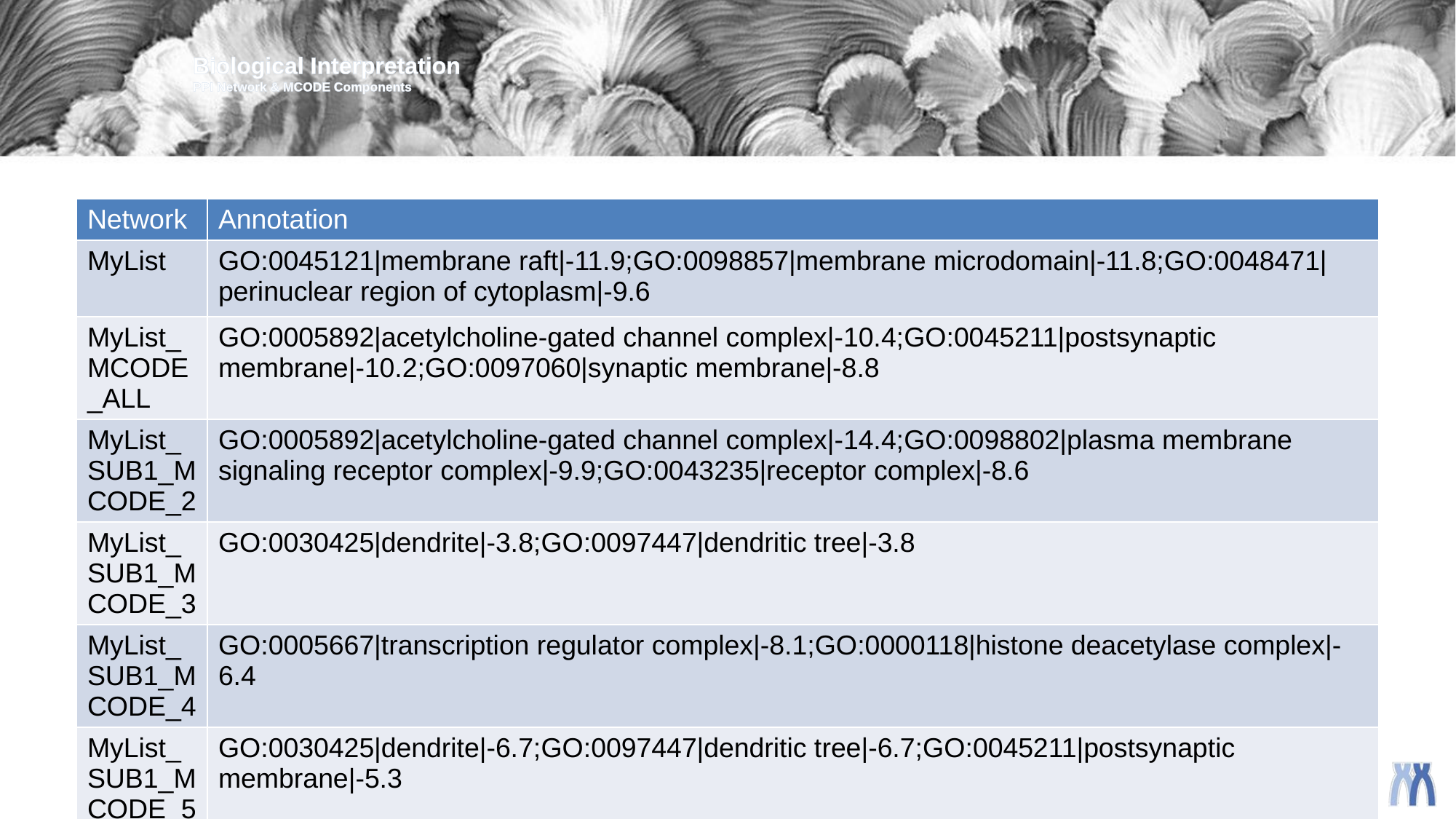

Biological Interpretation
PPI Network & MCODE Components
| Network | Annotation |
| --- | --- |
| MyList | GO:0045121|membrane raft|-11.9;GO:0098857|membrane microdomain|-11.8;GO:0048471|perinuclear region of cytoplasm|-9.6 |
| MyList\_MCODE\_ALL | GO:0005892|acetylcholine-gated channel complex|-10.4;GO:0045211|postsynaptic membrane|-10.2;GO:0097060|synaptic membrane|-8.8 |
| MyList\_SUB1\_MCODE\_2 | GO:0005892|acetylcholine-gated channel complex|-14.4;GO:0098802|plasma membrane signaling receptor complex|-9.9;GO:0043235|receptor complex|-8.6 |
| MyList\_SUB1\_MCODE\_3 | GO:0030425|dendrite|-3.8;GO:0097447|dendritic tree|-3.8 |
| MyList\_SUB1\_MCODE\_4 | GO:0005667|transcription regulator complex|-8.1;GO:0000118|histone deacetylase complex|-6.4 |
| MyList\_SUB1\_MCODE\_5 | GO:0030425|dendrite|-6.7;GO:0097447|dendritic tree|-6.7;GO:0045211|postsynaptic membrane|-5.3 |
| MyList\_SUB1\_MCODE\_6 | GO:0016323|basolateral plasma membrane|-5.6;GO:0009925|basal plasma membrane|-5.4;GO:0045178|basal part of cell|-5.3 |

## Slide 9
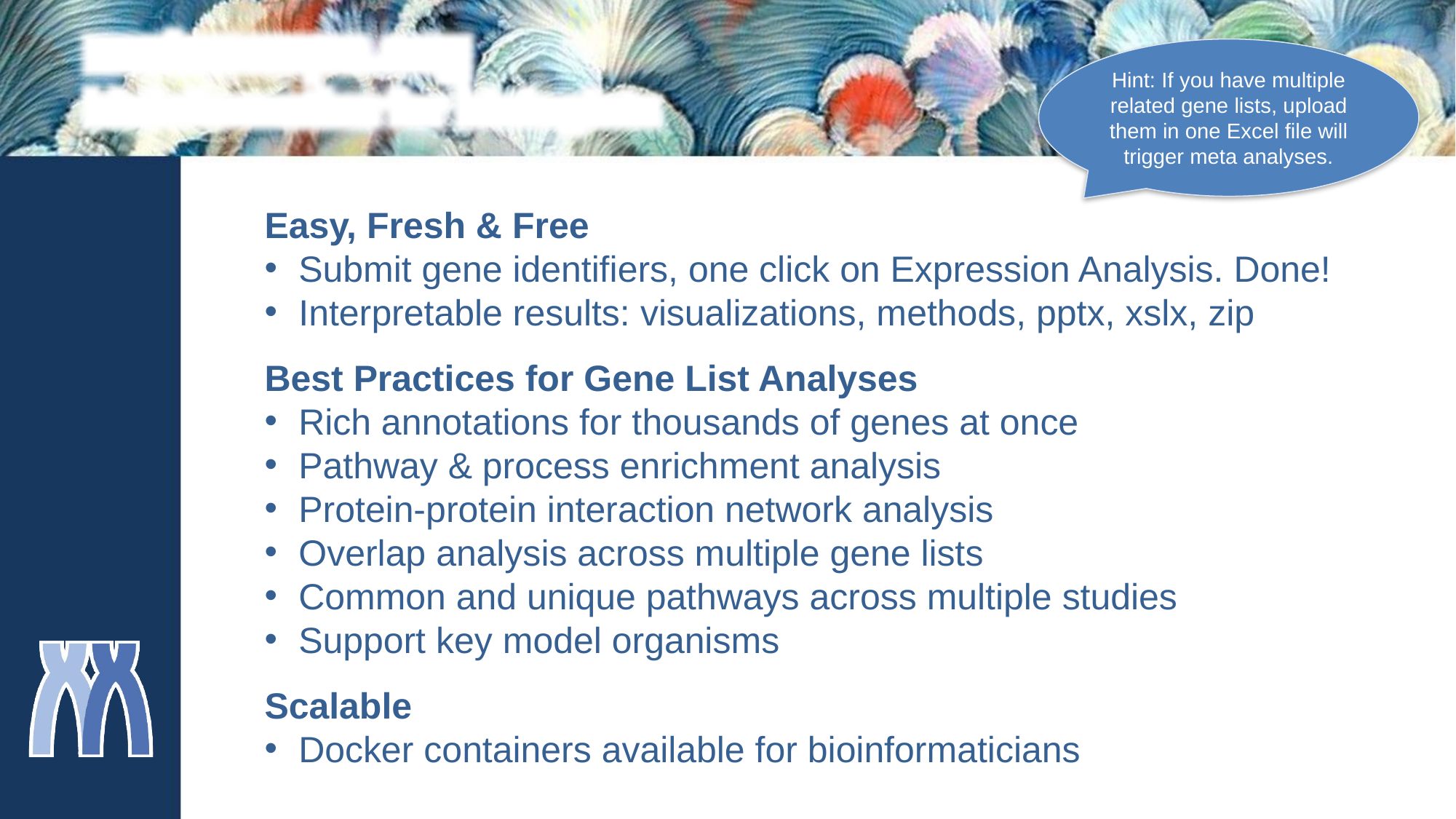

metascape.org
bioinformatics for biologists
Hint: If you have multiple related gene lists, upload them in one Excel file will trigger meta analyses.
Easy, Fresh & Free
Submit gene identifiers, one click on Expression Analysis. Done!
Interpretable results: visualizations, methods, pptx, xslx, zip
Best Practices for Gene List Analyses
Rich annotations for thousands of genes at once
Pathway & process enrichment analysis
Protein-protein interaction network analysis
Overlap analysis across multiple gene lists
Common and unique pathways across multiple studies
Support key model organisms
Scalable
Docker containers available for bioinformaticians
