# Supplementary material for: Investigation of the Mechanism of Cinnamaldehyde in Irritable Bowel Syndrome Based via Network Pharmacology, Molecular Docking, and Animal Experiments
Source: Pediatr Discov. 2025 Oct 5:e70017. Online ahead of print. doi: 10.1002/pdi3.70017 (PMC13398650; doi:10.1002/pdi3.70017)
Supplement: Supplementary file 1 — Supporting Information S1 [file PDI3-9999-0-s001.zip › Supplementary Materials/go kegg/cc/Enrichment_GO/ColorByCluster.pdf]

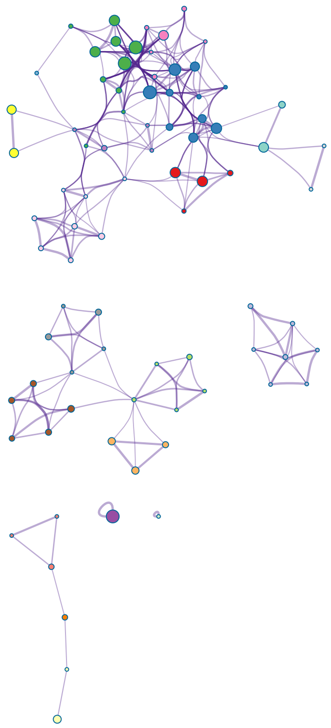

- membrane raft
- synaptic membrane
- dendrite
- perinuclear region of cytoplasm
- histone deacetylase complex
- focal adhesion
- ficolin-1-rich granule
- presynapse
- endosome lumen
- side of membrane
- transcription regulator complex
- protein kinase complex
- nuclear chromosome
- neuromuscular junction
- extracellular matrix
- tertiary granule
- basolateral plasma membrane
- cytoplasmic side of plasma membrane
- cell leading edge
- blood microparticle

created by
http://metascape.org
