# Supplementary figures and images for: Investigation of the Mechanism of Cinnamaldehyde in Irritable Bowel Syndrome Based via Network Pharmacology, Molecular Docking, and Animal Experiments
Source: Pediatr Discov. 2025 Oct 5:e70017. Online ahead of print. doi: 10.1002/pdi3.70017 (PMC13398650; doi:10.1002/pdi3.70017)

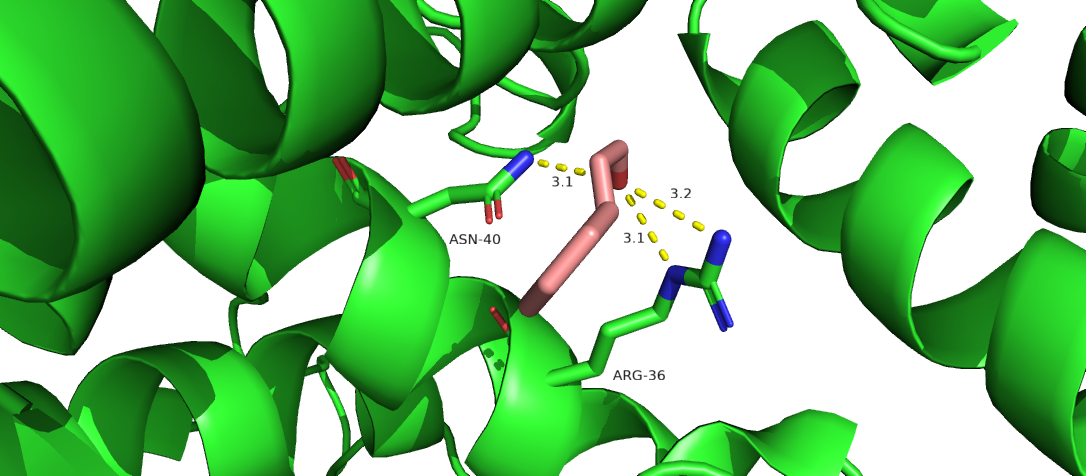

Supplement: Supplementary file 1 — Supporting Information S1 [file PDI3-9999-0-s001.zip › Supplementary Materials/Molecular Docking/HDAC1/4bkxQUGPQ.png]

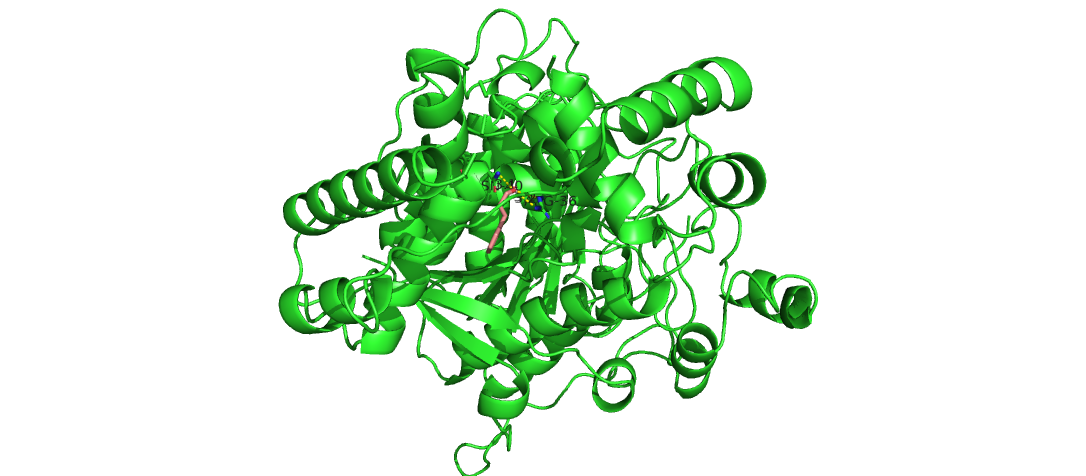

Supplement: Supplementary file 1 — Supporting Information S1 [file PDI3-9999-0-s001.zip › Supplementary Materials/Molecular Docking/HDAC1/4bkxQUGPQDA.png]

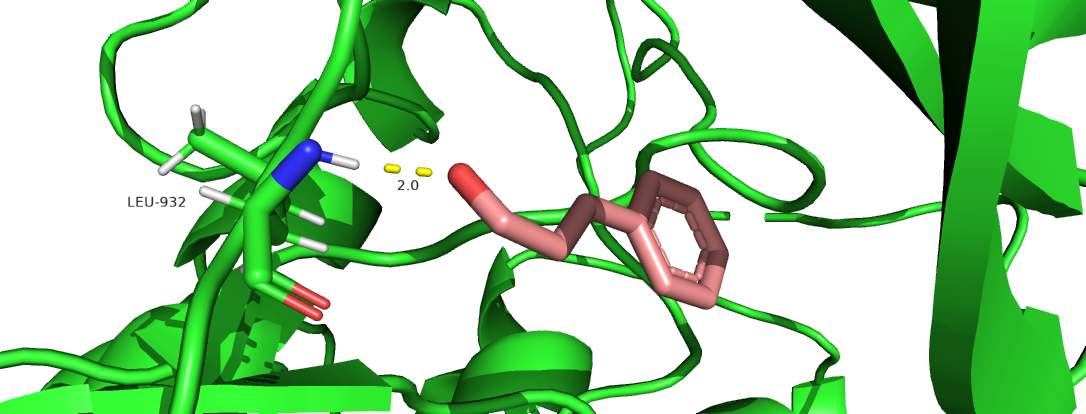

Supplement: Supplementary file 1 — Supporting Information S1 [file PDI3-9999-0-s001.zip › Supplementary Materials/Molecular Docking/JAK2/8bxhQUGPQ.png]

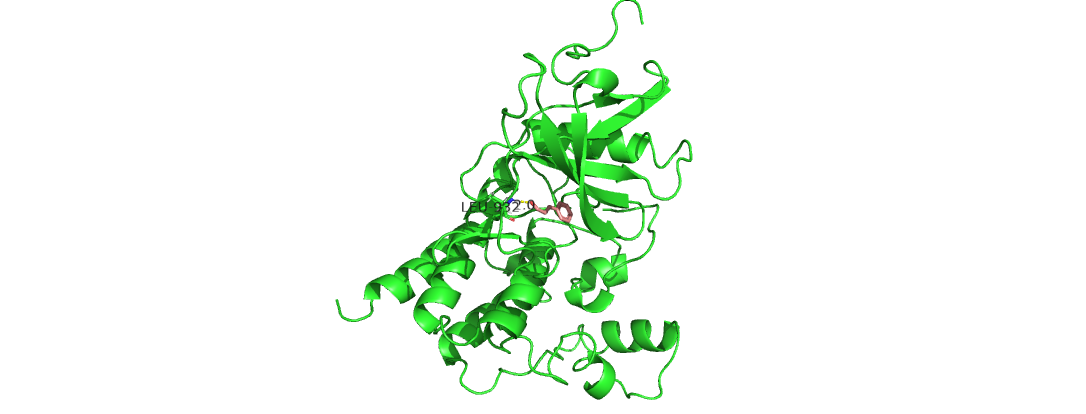

Supplement: Supplementary file 1 — Supporting Information S1 [file PDI3-9999-0-s001.zip › Supplementary Materials/Molecular Docking/JAK2/8bxhQUGPQDA.png]

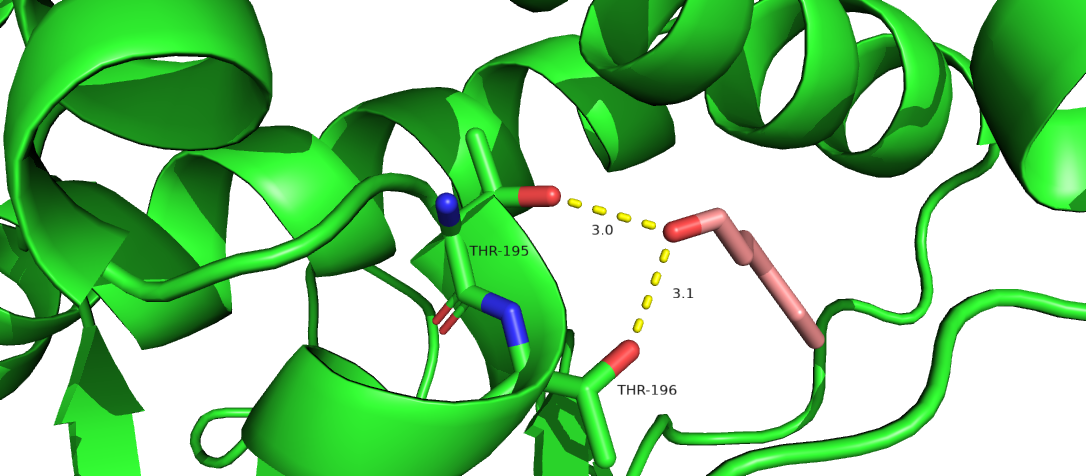

Supplement: Supplementary file 1 — Supporting Information S1 [file PDI3-9999-0-s001.zip › Supplementary Materials/Molecular Docking/MAOB/2xfnQUGPQ.png]

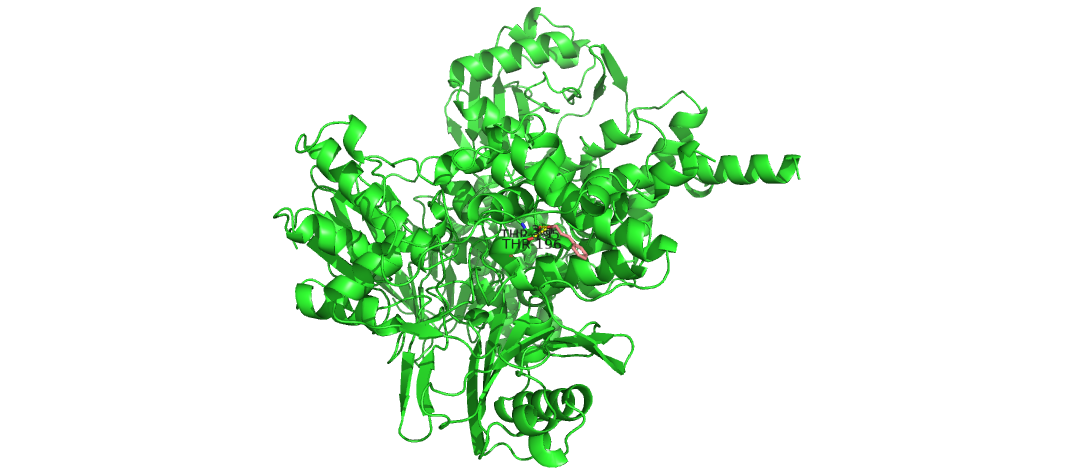

Supplement: Supplementary file 1 — Supporting Information S1 [file PDI3-9999-0-s001.zip › Supplementary Materials/Molecular Docking/MAOB/2xfnQUGPQDA.png]

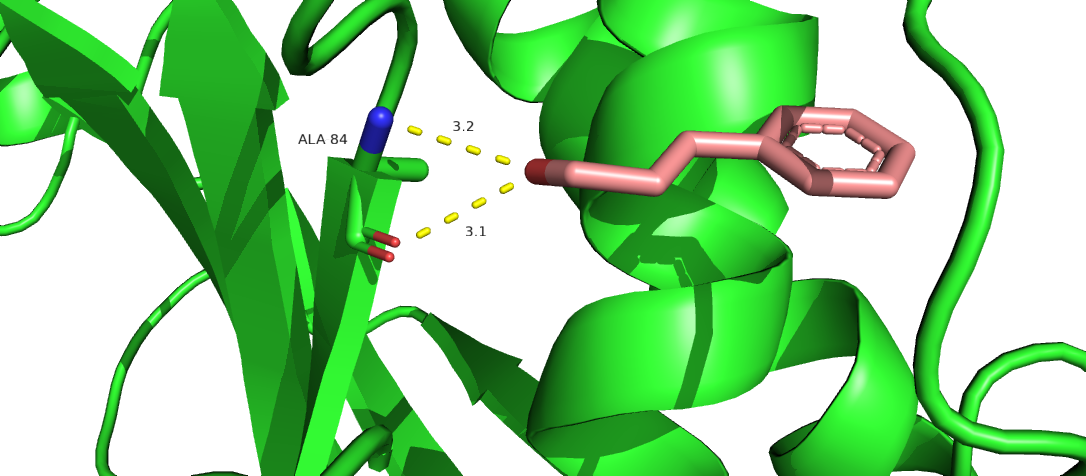

Supplement: Supplementary file 1 — Supporting Information S1 [file PDI3-9999-0-s001.zip › Supplementary Materials/Molecular Docking/MMP2/7xjoQUGPQ.png]

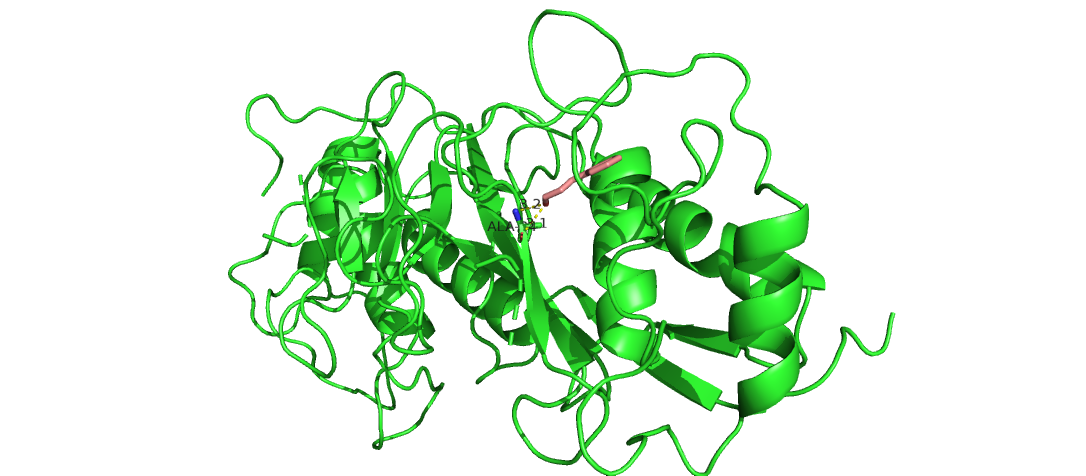

Supplement: Supplementary file 1 — Supporting Information S1 [file PDI3-9999-0-s001.zip › Supplementary Materials/Molecular Docking/MMP2/7xjoQUGPQDA.png]

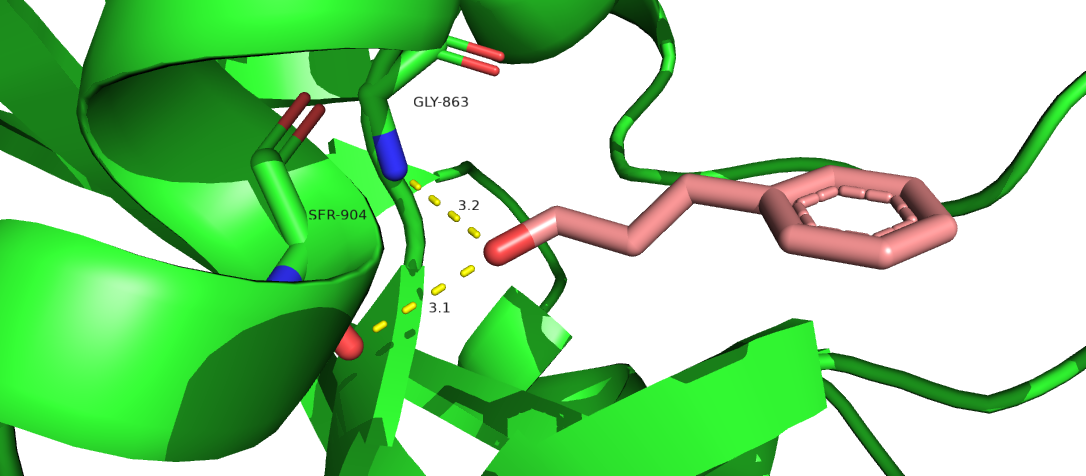

Supplement: Supplementary file 1 — Supporting Information S1 [file PDI3-9999-0-s001.zip › Supplementary Materials/Molecular Docking/PARP1/7aacQUGPQ.png]

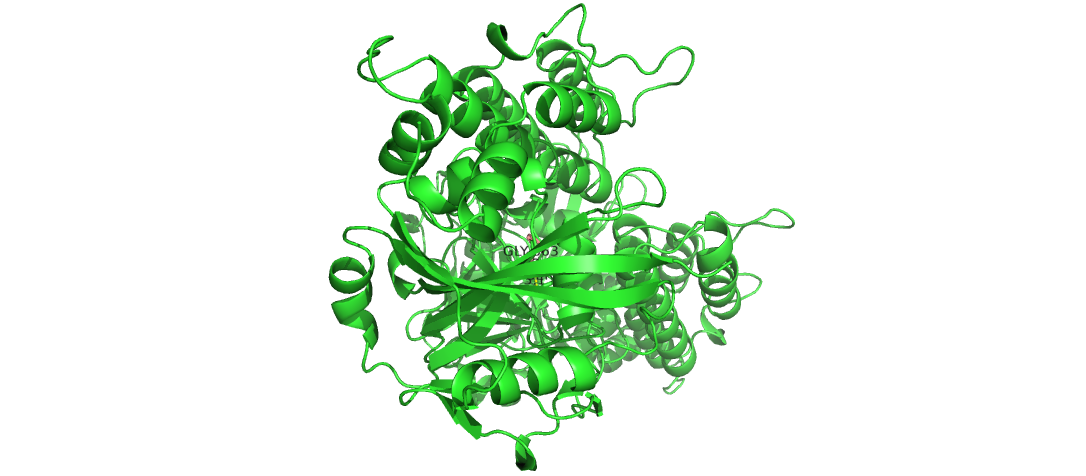

Supplement: Supplementary file 1 — Supporting Information S1 [file PDI3-9999-0-s001.zip › Supplementary Materials/Molecular Docking/PARP1/7aacQUGPQDA.png]

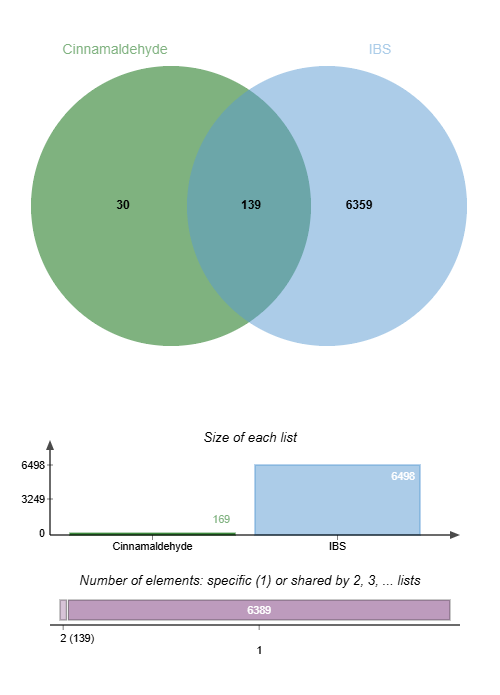

Supplement: Supplementary file 1 — Supporting Information S1 [file PDI3-9999-0-s001.zip › Supplementary Materials/Network/jVenn_chart.png]

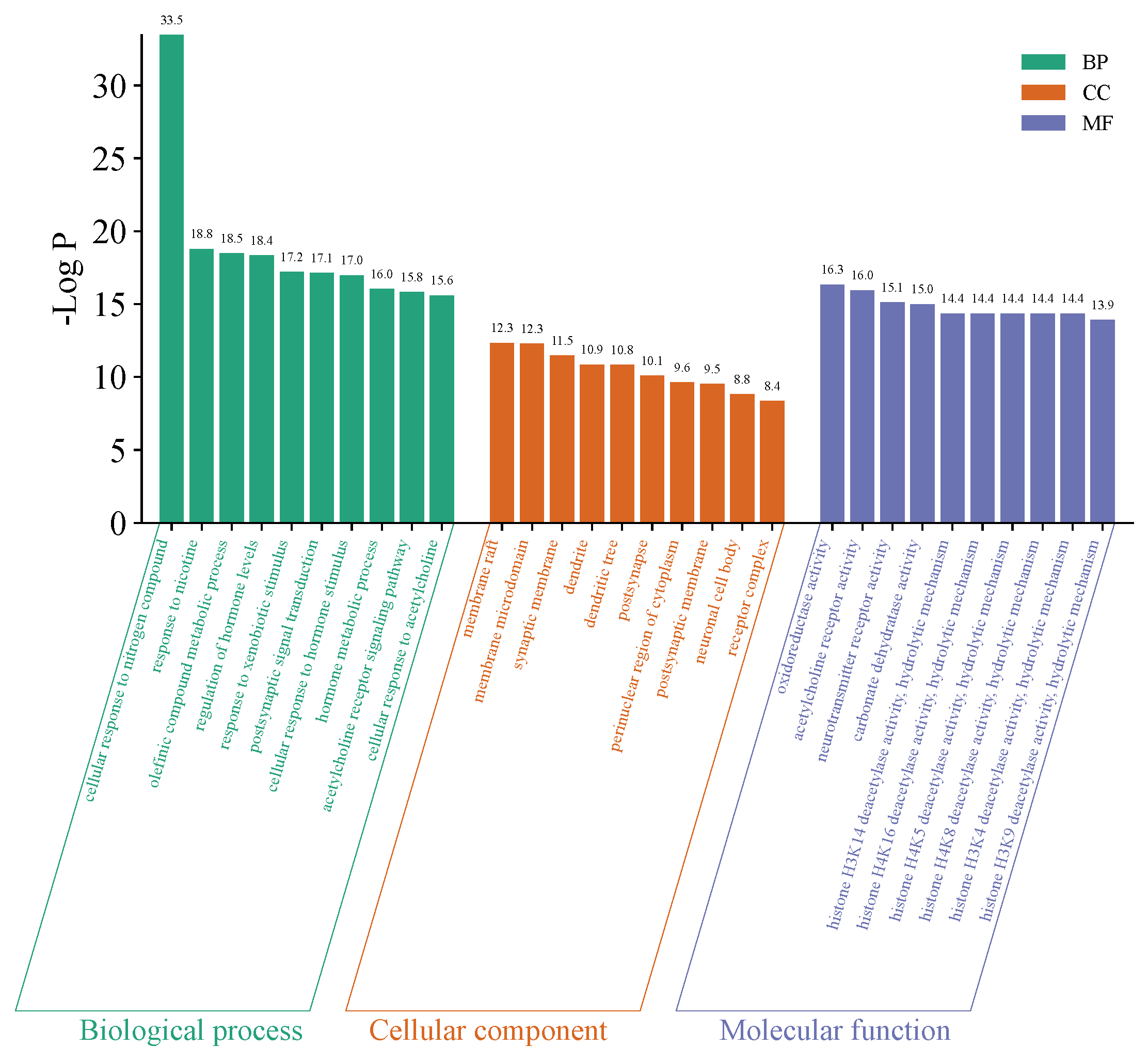

Supplement: Supplementary file 1 — Supporting Information S1 [file PDI3-9999-0-s001.zip › Supplementary Materials/go kegg/GO.png]

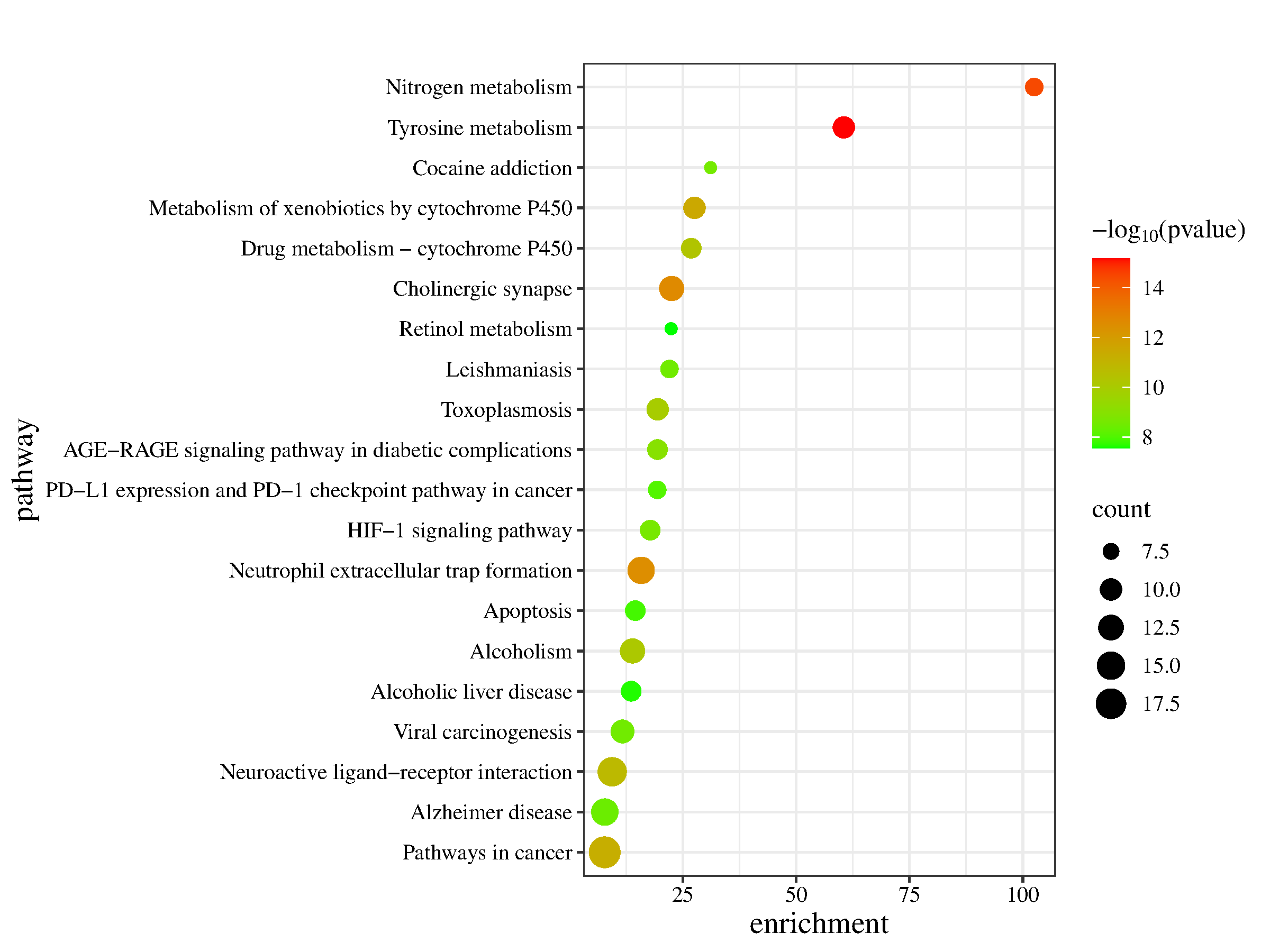

Supplement: Supplementary file 1 — Supporting Information S1 [file PDI3-9999-0-s001.zip › Supplementary Materials/go kegg/KEGG.png]

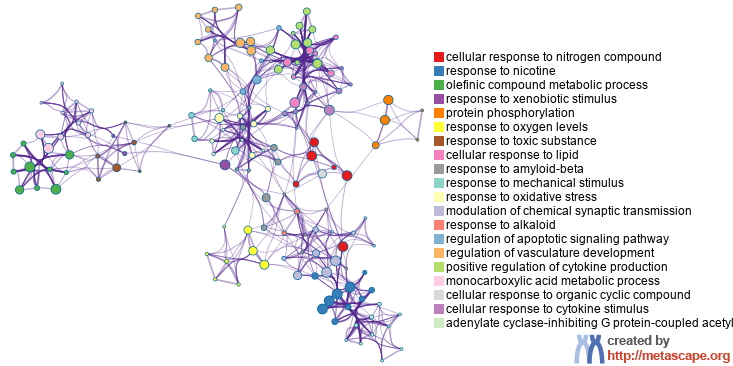

Supplement: Supplementary file 1 — Supporting Information S1 [file PDI3-9999-0-s001.zip › Supplementary Materials/go kegg/bp/Enrichment_GO/ColorByCluster.png]

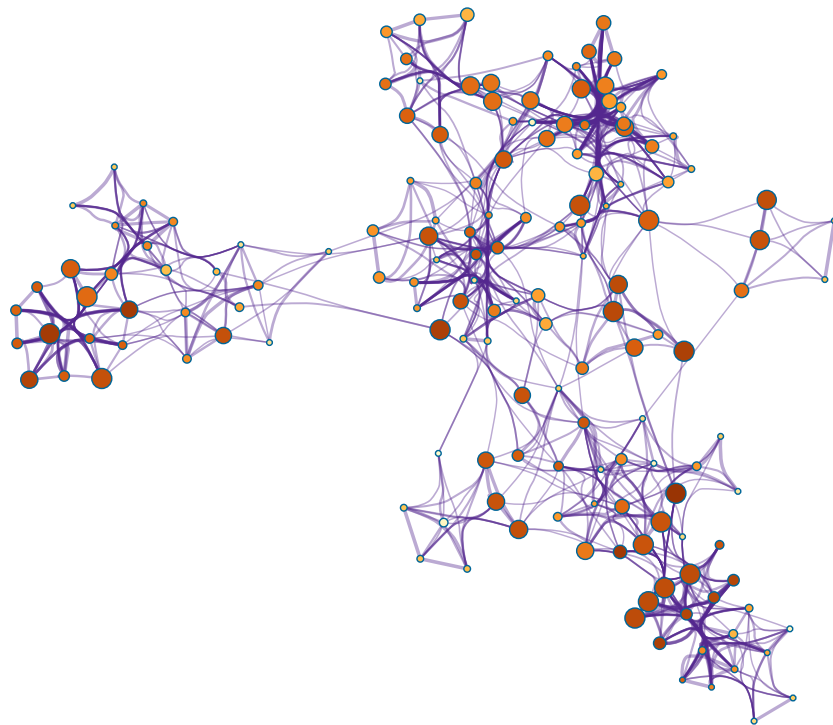

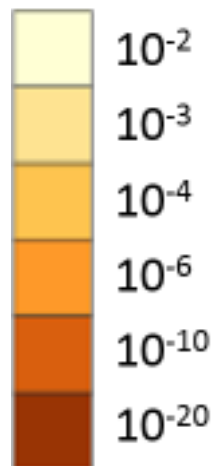

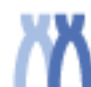 created by  
<http://metascape.org>

Supplement: Supplementary file 1 — Supporting Information S1 [file PDI3-9999-0-s001.zip › Supplementary Materials/go kegg/bp/Enrichment_GO/ColorByPValue.pdf]

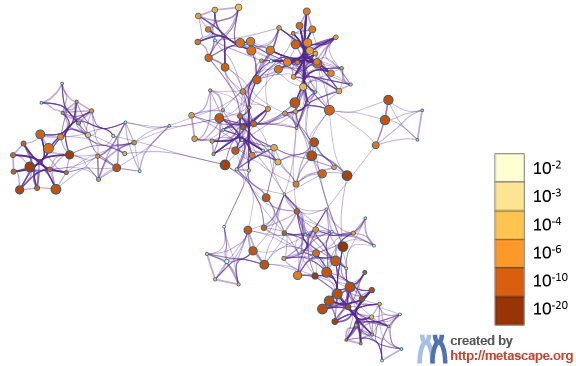

Supplement: Supplementary file 1 — Supporting Information S1 [file PDI3-9999-0-s001.zip › Supplementary Materials/go kegg/bp/Enrichment_GO/ColorByPValue.png]

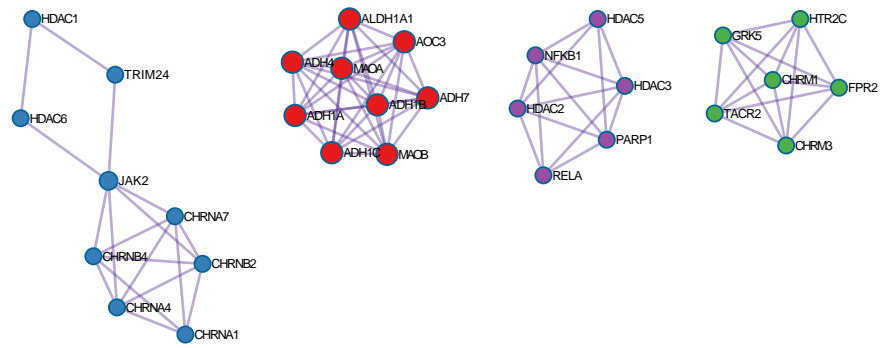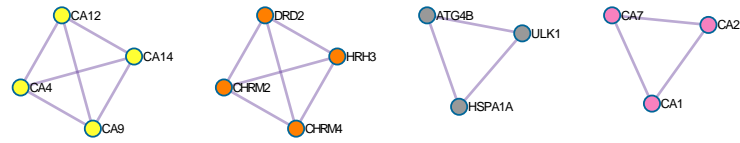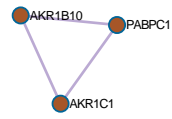

- MCODE1
- MCODE2
- MCODE3
- MCODE4
- MCODE5
- MCODE6
- MCODE7
- MCODE8
- MCODE9

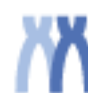 created by  
<http://metascape.org>

Supplement: Supplementary file 1 — Supporting Information S1 [file PDI3-9999-0-s001.zip › Supplementary Materials/go kegg/bp/Enrichment_PPI/MyList_MCODE_ALL_PPIColorByCluster.pdf]

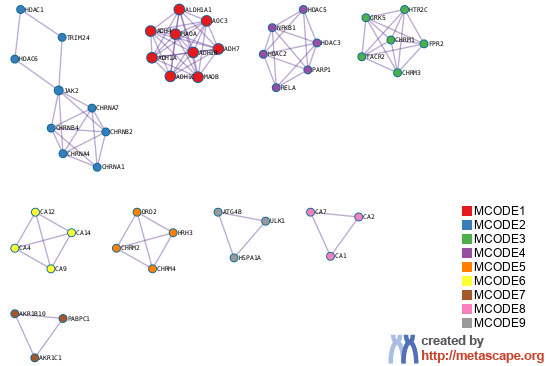

Supplement: Supplementary file 1 — Supporting Information S1 [file PDI3-9999-0-s001.zip › Supplementary Materials/go kegg/bp/Enrichment_PPI/MyList_MCODE_ALL_PPIColorByCluster.png]

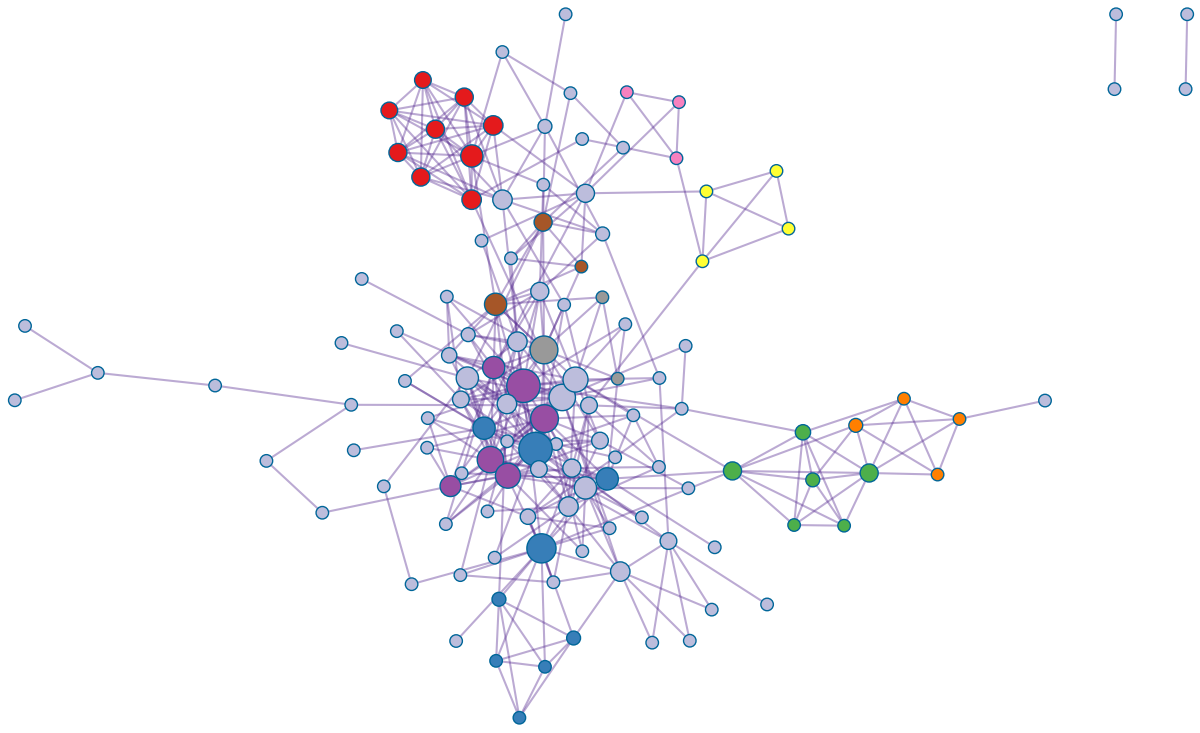

- MCODE1
- MCODE2
- MCODE3
- MCODE4
- MCODE5
- MCODE6
- MCODE7
- MCODE8
- MCODE9

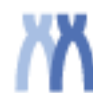 created by  
<http://metascape.org>

Supplement: Supplementary file 1 — Supporting Information S1 [file PDI3-9999-0-s001.zip › Supplementary Materials/go kegg/bp/Enrichment_PPI/MyList_PPIColorByCluster.pdf]

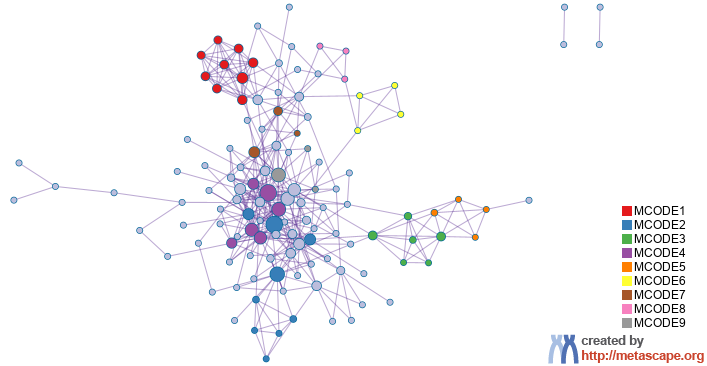

Supplement: Supplementary file 1 — Supporting Information S1 [file PDI3-9999-0-s001.zip › Supplementary Materials/go kegg/bp/Enrichment_PPI/MyList_PPIColorByCluster.png]

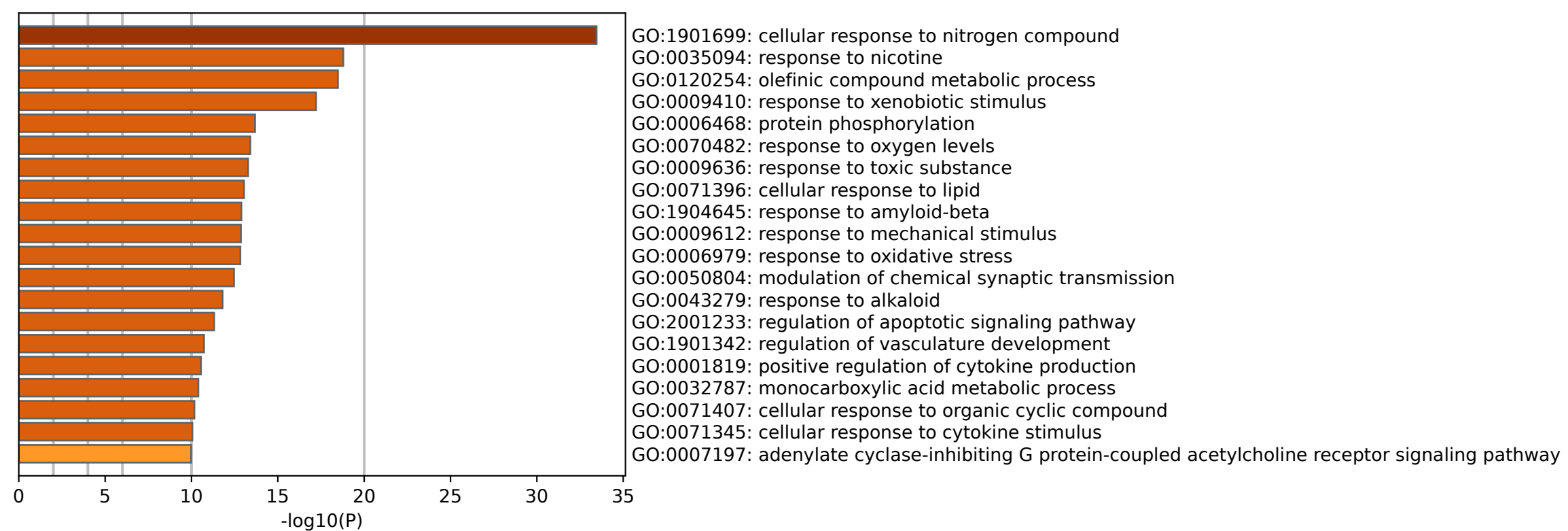

Supplement: Supplementary file 1 — Supporting Information S1 [file PDI3-9999-0-s001.zip › Supplementary Materials/go kegg/bp/Enrichment_heatmap/HeatmapSelectedGO.pdf]

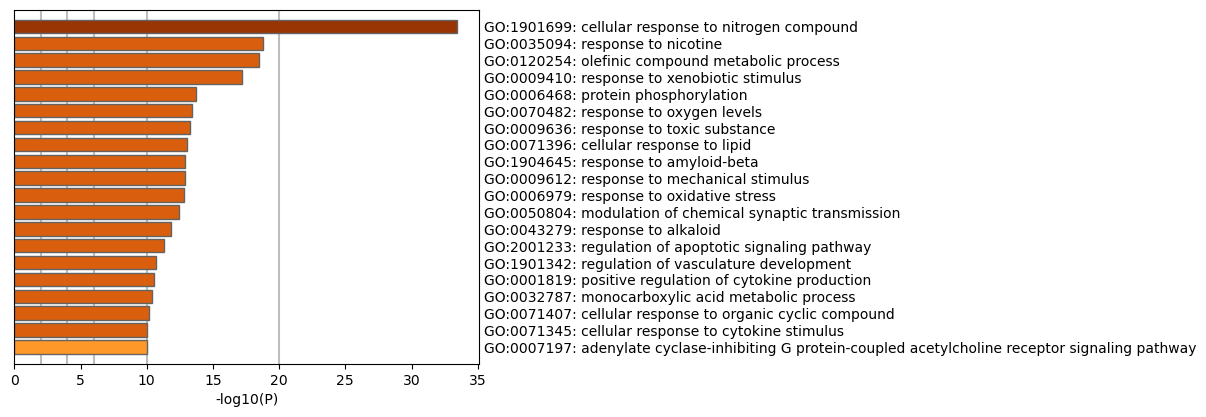

Supplement: Supplementary file 1 — Supporting Information S1 [file PDI3-9999-0-s001.zip › Supplementary Materials/go kegg/bp/Enrichment_heatmap/HeatmapSelectedGO.png]

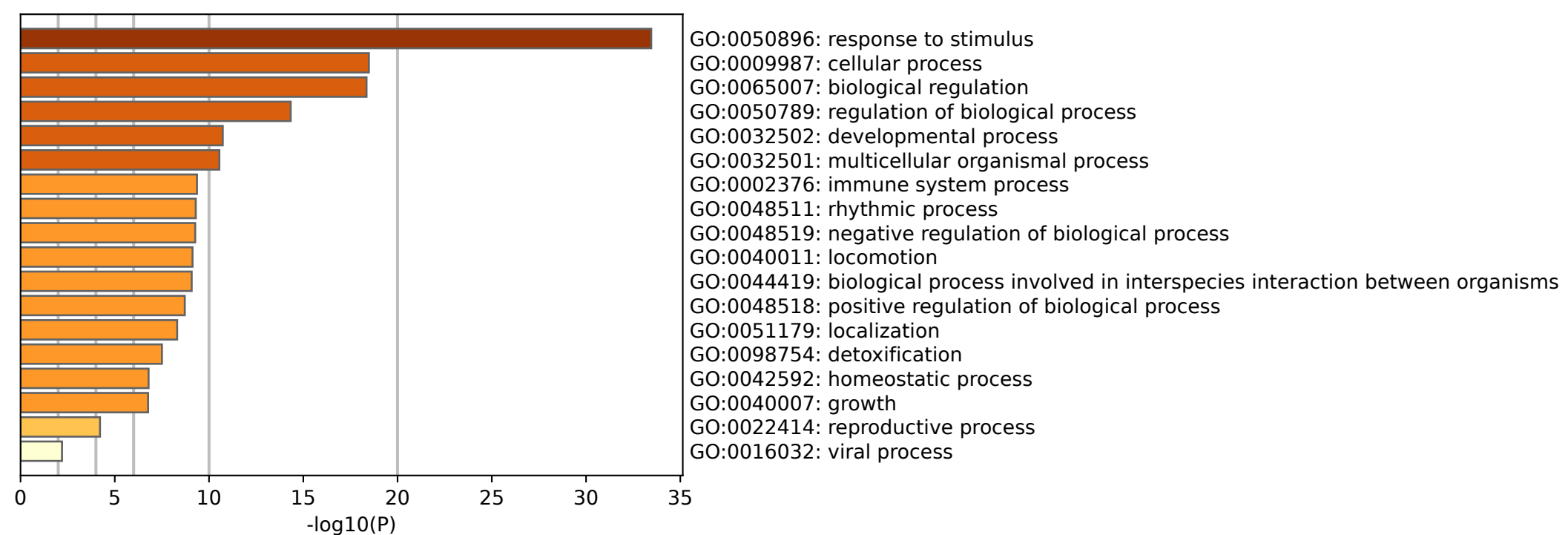

Supplement: Supplementary file 1 — Supporting Information S1 [file PDI3-9999-0-s001.zip › Supplementary Materials/go kegg/bp/Enrichment_heatmap/HeatmapSelectedGOParent.pdf]

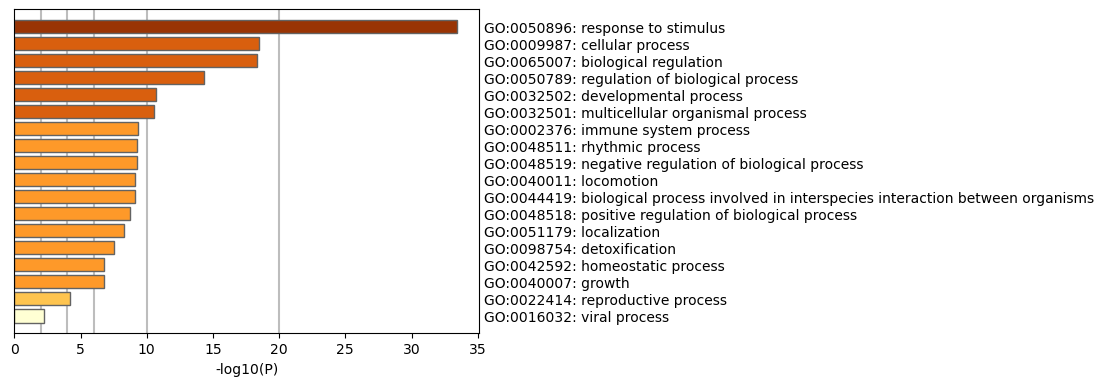

Supplement: Supplementary file 1 — Supporting Information S1 [file PDI3-9999-0-s001.zip › Supplementary Materials/go kegg/bp/Enrichment_heatmap/HeatmapSelectedGOParent.png]

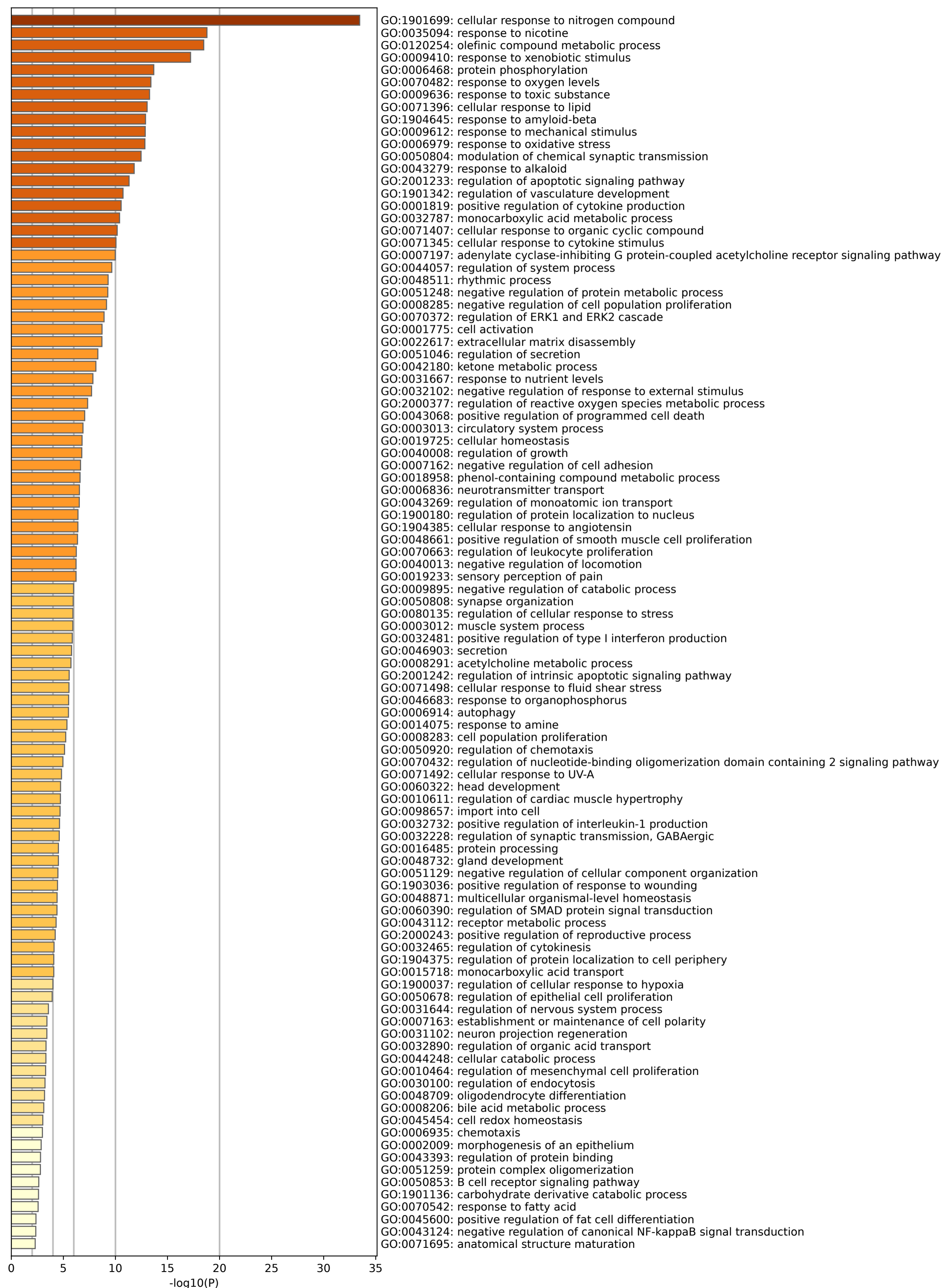

Supplement: Supplementary file 1 — Supporting Information S1 [file PDI3-9999-0-s001.zip › Supplementary Materials/go kegg/bp/Enrichment_heatmap/HeatmapSelectedGOTop100.pdf]

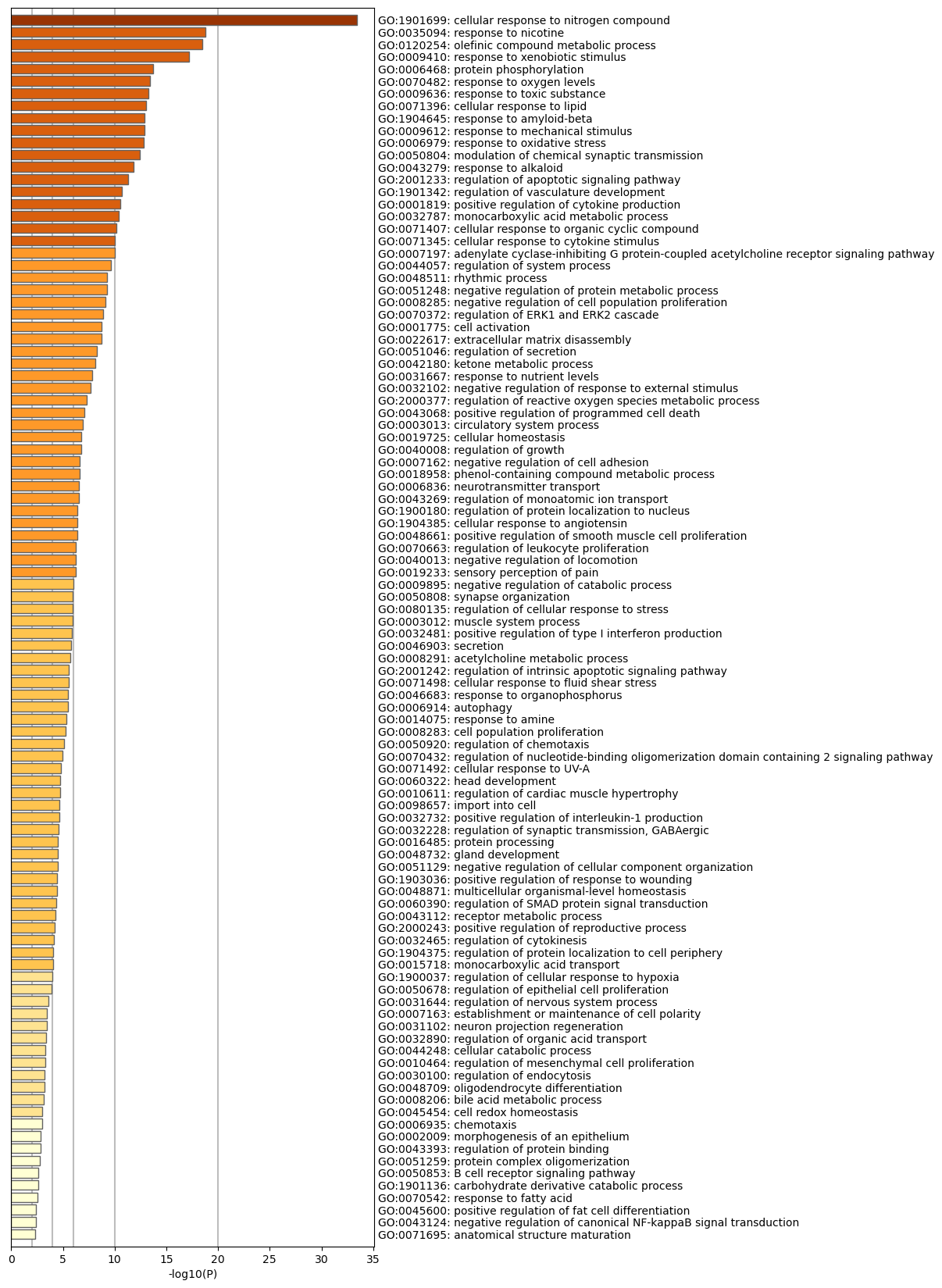

Supplement: Supplementary file 1 — Supporting Information S1 [file PDI3-9999-0-s001.zip › Supplementary Materials/go kegg/bp/Enrichment_heatmap/HeatmapSelectedGOTop100.png]

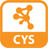

Supplement: Supplementary file 1 — Supporting Information S1 [file PDI3-9999-0-s001.zip › Supplementary Materials/go kegg/bp/icon/CYS48.png]

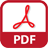

Supplement: Supplementary file 1 — Supporting Information S1 [file PDI3-9999-0-s001.zip › Supplementary Materials/go kegg/bp/icon/PDF48.png]

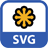

Supplement: Supplementary file 1 — Supporting Information S1 [file PDI3-9999-0-s001.zip › Supplementary Materials/go kegg/bp/icon/SVG48.png]

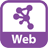

Supplement: Supplementary file 1 — Supporting Information S1 [file PDI3-9999-0-s001.zip › Supplementary Materials/go kegg/bp/icon/WEB_CYS48.png]

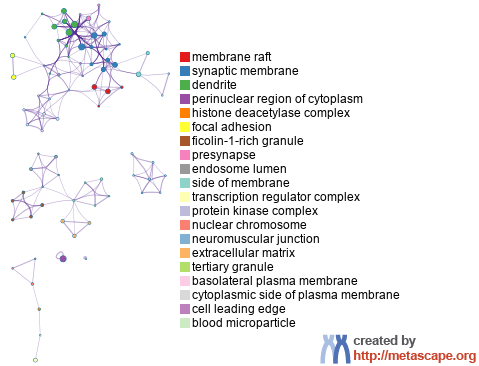

Supplement: Supplementary file 1 — Supporting Information S1 [file PDI3-9999-0-s001.zip › Supplementary Materials/go kegg/cc/Enrichment_GO/ColorByCluster.png]

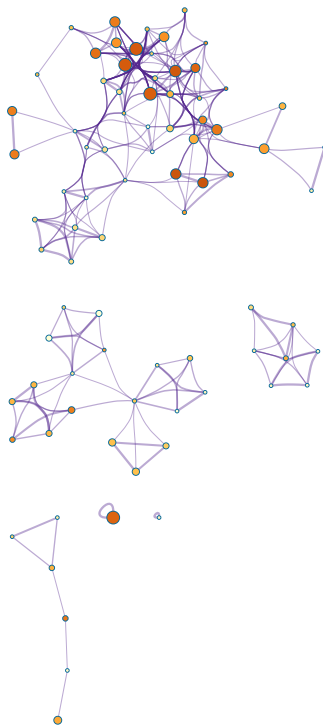

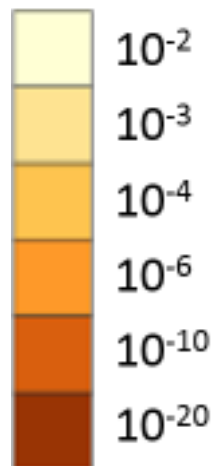

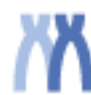 created by  
<http://metascape.org>

Supplement: Supplementary file 1 — Supporting Information S1 [file PDI3-9999-0-s001.zip › Supplementary Materials/go kegg/cc/Enrichment_GO/ColorByPValue.pdf]

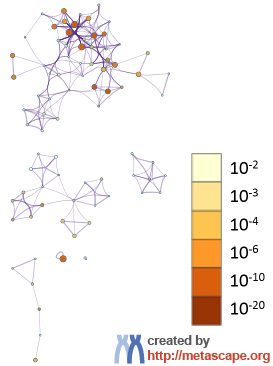

Supplement: Supplementary file 1 — Supporting Information S1 [file PDI3-9999-0-s001.zip › Supplementary Materials/go kegg/cc/Enrichment_GO/ColorByPValue.png]
