# Supplementary material for: Investigation of the Mechanism of Cinnamaldehyde in Irritable Bowel Syndrome Based via Network Pharmacology, Molecular Docking, and Animal Experiments
Source: Pediatr Discov. 2025 Oct 5:e70017. Online ahead of print. doi: 10.1002/pdi3.70017 (PMC13398650; doi:10.1002/pdi3.70017)
Supplement: Supplementary file 1 — Supporting Information S1 [file PDI3-9999-0-s001.zip › Supplementary Materials/Table S1.doc]

**Table S1.** The AWR scoring criteria

| Score | Criteria |
| --- | --- |
| 0 | No behavioral response to CRD. |
| 1 | Brief head movement followed by immobility. |
| 2 | Contraction of abdominal muscles. |
| 3 | Lifting of abdomen. |
| 4 | Body arching and lifting of pelvic structures. |
