# Supplementary material for: Investigation of the Mechanism of Cinnamaldehyde in Irritable Bowel Syndrome Based via Network Pharmacology, Molecular Docking, and Animal Experiments
Source: Pediatr Discov. 2025 Oct 5:e70017. Online ahead of print. doi: 10.1002/pdi3.70017 (PMC13398650; doi:10.1002/pdi3.70017)
Supplement: Supplementary file 1 — Supporting Information S1 [file PDI3-9999-0-s001.zip › Supplementary Materials/go kegg/cc/Enrichment_PPI/MyList_MCODE_ALL_PPIColorByCluster.pdf]

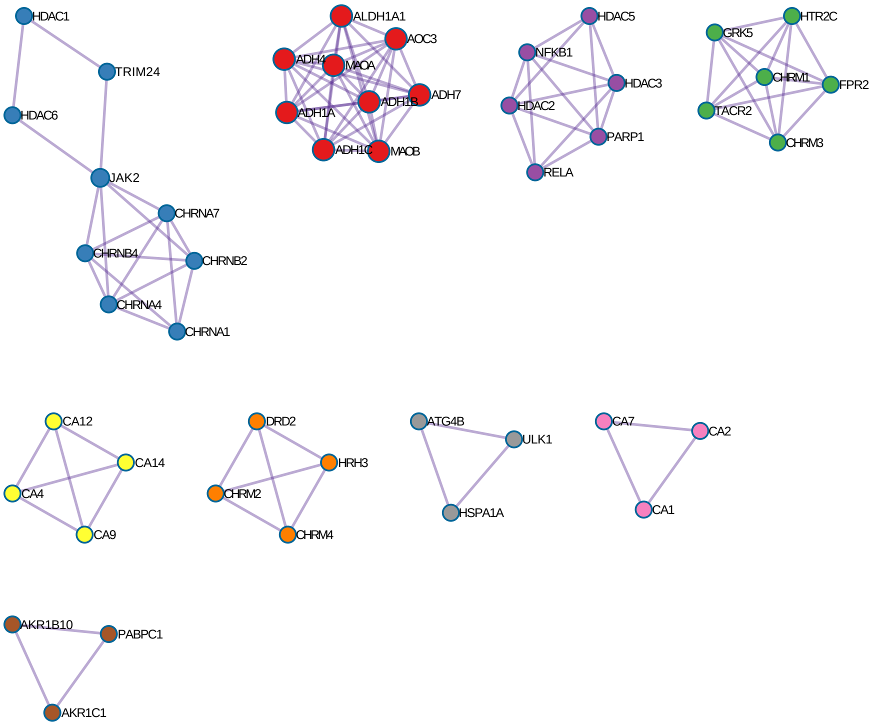

HDAC1
TRIM24
HDAC6
JAK2
CHRNA7
CHRNB4
CHRNB2
CHRNA4
CHRNA1
ALDH1A1
AOC3
ADH4
MAOA
ADH7
ADH1B
ADH1A
ADH1C
MAOB
HDAC5
NFKB1
HDAC3
HDAC2
PARP1
RELA
GRK5
HTR2C
CHRM1
FPR2
TACR2
CHRM3
CA12
CA14
CA4
CA9
DRD2
HRH3
CHRM2
CHRM4
ATG4B
ULK1
HSPA1A
CA7
CA2
CA1
AKR1B10
PABPC1
AKR1C1

MCODE1
MCODE2
MCODE3
MCODE4
MCODE5
MCODE6
MCODE7
MCODE8
MCODE9

created by
http://metascape.org
