# Supplementary material for: Investigation of the Mechanism of Cinnamaldehyde in Irritable Bowel Syndrome Based via Network Pharmacology, Molecular Docking, and Animal Experiments
Source: Pediatr Discov. 2025 Oct 5:e70017. Online ahead of print. doi: 10.1002/pdi3.70017 (PMC13398650; doi:10.1002/pdi3.70017)
Supplement: Supplementary file 1 — Supporting Information S1 [file PDI3-9999-0-s001.zip › Supplementary Materials/go kegg/cc/Enrichment_heatmap/HeatmapSelectedGO.pdf]

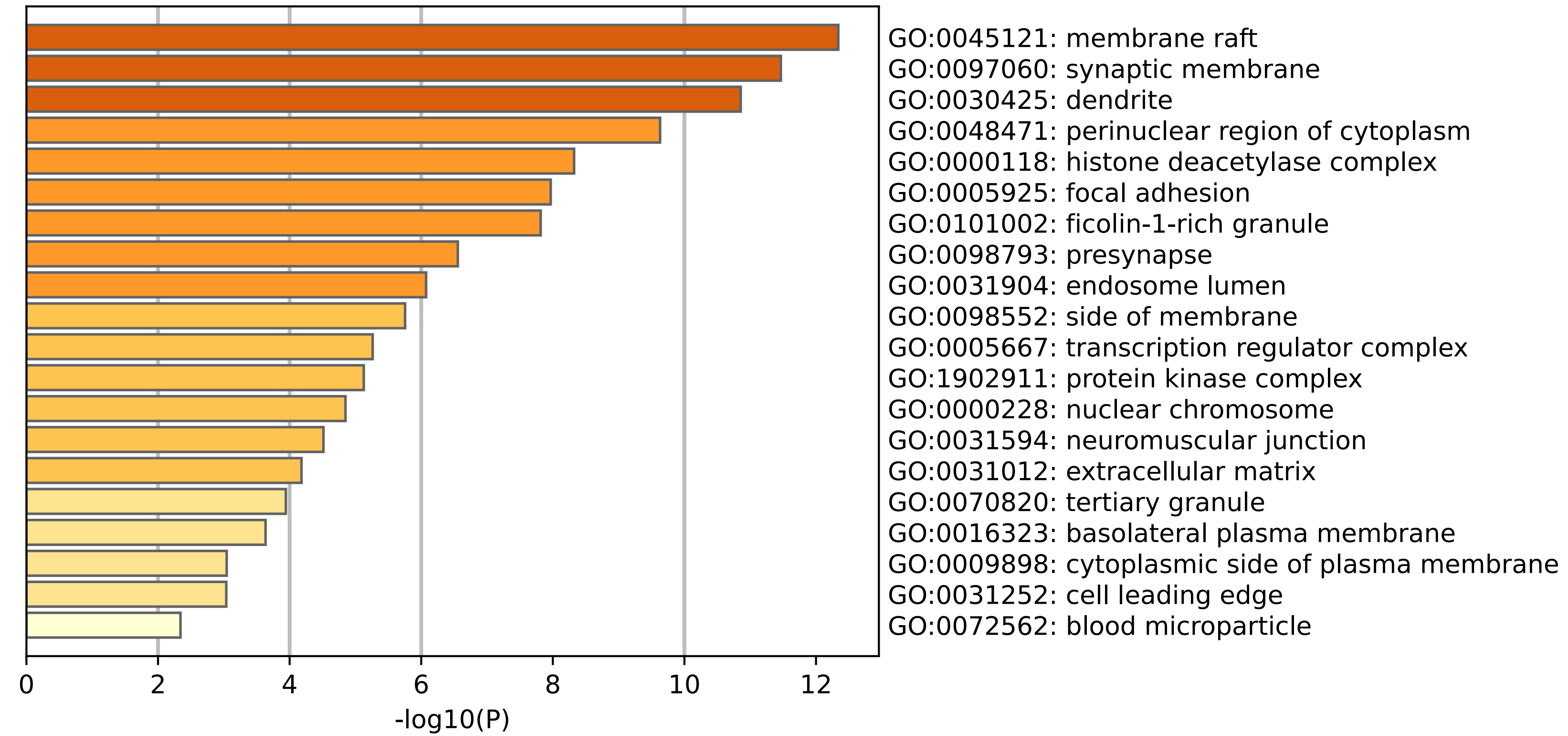
GO:0045121: membrane raft
GO:0097060: synaptic membrane
GO:0030425: dendrite
GO:0048471: perinuclear region of cytoplasm
GO:0000118: histone deacetylase complex
GO:0005925: focal adhesion
GO:0101002: ficolin-1-rich granule
GO:0098793: presynapse
GO:0031904: endosome lumen
GO:0098552: side of membrane
GO:0005667: transcription regulator complex
GO:1902911: protein kinase complex
GO:0000228: nuclear chromosome
GO:0031594: neuromuscular junction
GO:0031012: extracellular matrix
GO:0070820: tertiary granule
GO:0016323: basolateral plasma membrane
GO:0009898: cytoplasmic side of plasma membrane
GO:0031252: cell leading edge
GO:0072562: blood microparticle
0
2
4
6
8
10
12
-log10(P)
