# Supplementary material for: Investigation of the Mechanism of Cinnamaldehyde in Irritable Bowel Syndrome Based via Network Pharmacology, Molecular Docking, and Animal Experiments
Source: Pediatr Discov. 2025 Oct 5:e70017. Online ahead of print. doi: 10.1002/pdi3.70017 (PMC13398650; doi:10.1002/pdi3.70017)
Supplement: Supplementary file 1 — Supporting Information S1 [file PDI3-9999-0-s001.zip › Supplementary Materials/go kegg/kegg/Enrichment_GO/ColorByCluster.pdf]

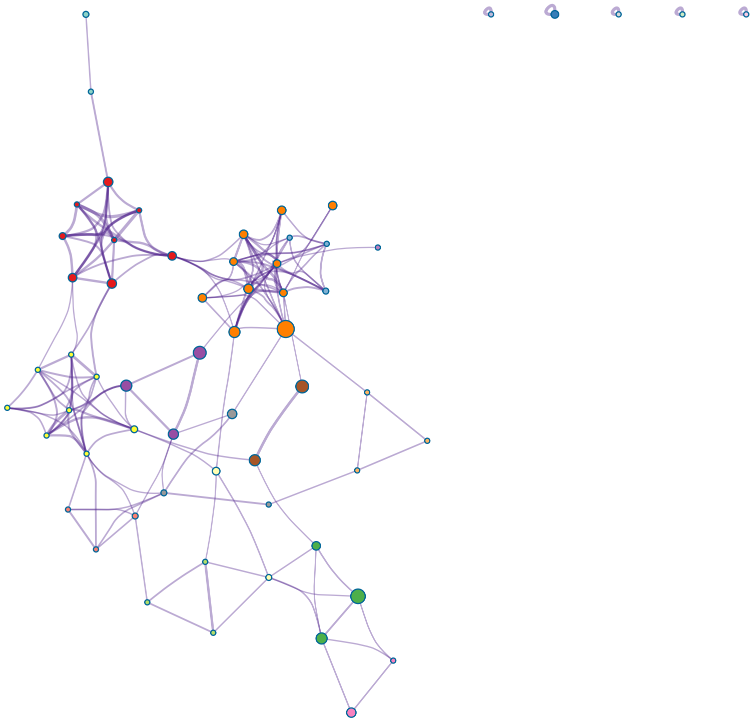

- Tyrosine metabolism
- Nitrogen metabolism
- Cholinergic synapse
- Neutrophil extracellular trap formation
- Pathways in cancer
- Cocaine addiction
- Alzheimer disease
- Chemical carcinogenesis - receptor activation
- MicroRNAs in cancer
- Drug metabolism - other enzymes
- cAMP signaling pathway
- Steroid hormone biosynthesis
- Thyroid hormone signaling pathway
- Necroptosis
- FoxO signaling pathway
- Gastric acid secretion
- Apelin signaling pathway
- Bladder cancer
- Rheumatoid arthritis
- Base excision repair

created by
http://metascape.org
