# Supplementary material for: Investigation of the Mechanism of Cinnamaldehyde in Irritable Bowel Syndrome Based via Network Pharmacology, Molecular Docking, and Animal Experiments
Source: Pediatr Discov. 2025 Oct 5:e70017. Online ahead of print. doi: 10.1002/pdi3.70017 (PMC13398650; doi:10.1002/pdi3.70017)
Supplement: Supplementary file 1 — Supporting Information S1 [file PDI3-9999-0-s001.zip › Supplementary Materials/go kegg/kegg/Enrichment_PPI/MyList_PPIColorByCluster.pdf]

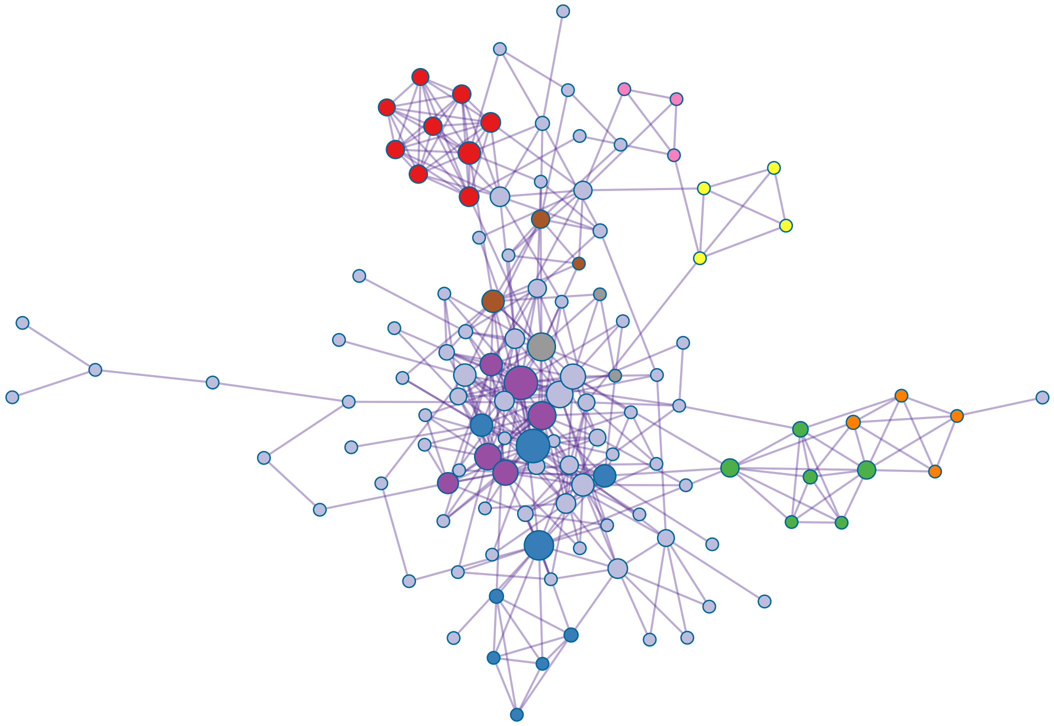

MCODE1
MCODE2
MCODE3
MCODE4
MCODE5
MCODE6
MCODE7
MCODE8
MCODE9

created by
http://metascape.org
