# Supplementary material for: Investigation of the Mechanism of Cinnamaldehyde in Irritable Bowel Syndrome Based via Network Pharmacology, Molecular Docking, and Animal Experiments
Source: Pediatr Discov. 2025 Oct 5:e70017. Online ahead of print. doi: 10.1002/pdi3.70017 (PMC13398650; doi:10.1002/pdi3.70017)
Supplement: Supplementary file 1 — Supporting Information S1 [file PDI3-9999-0-s001.zip › Supplementary Materials/go kegg/kegg/Enrichment_heatmap/HeatmapSelectedGOTop100.pdf]

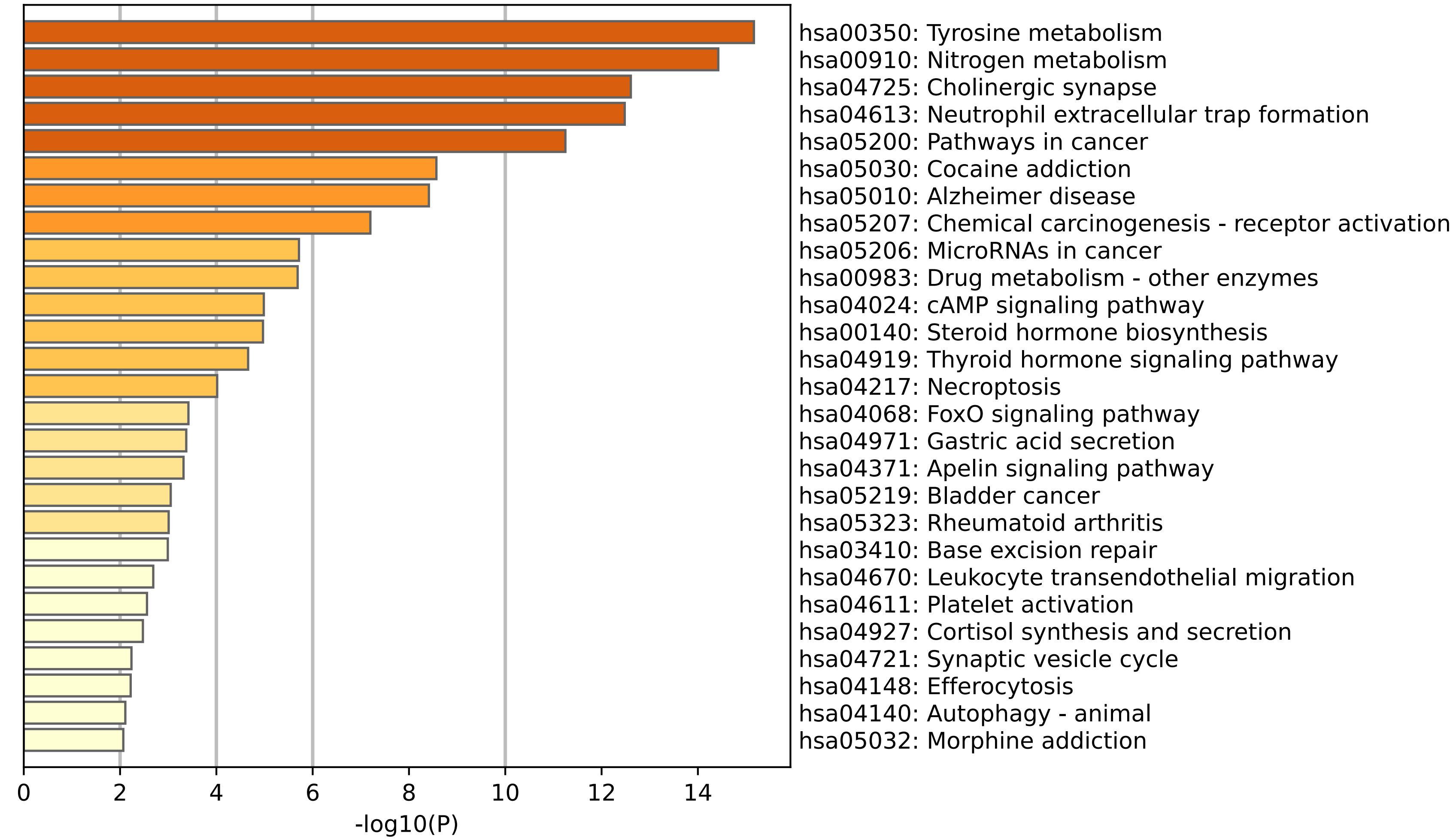
hsa00350: Tyrosine metabolism
hsa00910: Nitrogen metabolism
hsa04725: Cholinergic synapse
hsa04613: Neutrophil extracellular trap formation
hsa05200: Pathways in cancer
hsa05030: Cocaine addiction
hsa05010: Alzheimer disease
hsa05207: Chemical carcinogenesis - receptor activation
hsa05206: MicroRNAs in cancer
hsa00983: Drug metabolism - other enzymes
hsa04024: cAMP signaling pathway
hsa00140: Steroid hormone biosynthesis
hsa04919: Thyroid hormone signaling pathway
hsa04217: Necroptosis
hsa04068: FoxO signaling pathway
hsa04971: Gastric acid secretion
hsa04371: Apelin signaling pathway
hsa05219: Bladder cancer
hsa05323: Rheumatoid arthritis
hsa03410: Base excision repair
hsa04670: Leukocyte transendothelial migration
hsa04611: Platelet activation
hsa04927: Cortisol synthesis and secretion
hsa04721: Synaptic vesicle cycle
hsa04148: Efferocytosis
hsa04140: Autophagy - animal
hsa05032: Morphine addiction
0
2
4
6
8
10
12
14
-log10(P)
