# Supplementary material for: Investigation of the Mechanism of Cinnamaldehyde in Irritable Bowel Syndrome Based via Network Pharmacology, Molecular Docking, and Animal Experiments
Source: Pediatr Discov. 2025 Oct 5:e70017. Online ahead of print. doi: 10.1002/pdi3.70017 (PMC13398650; doi:10.1002/pdi3.70017)
Supplement: Supplementary file 1 — Supporting Information S1 [file PDI3-9999-0-s001.zip › Supplementary Materials/go kegg/mf/Enrichment_GO/ColorByCluster.pdf]

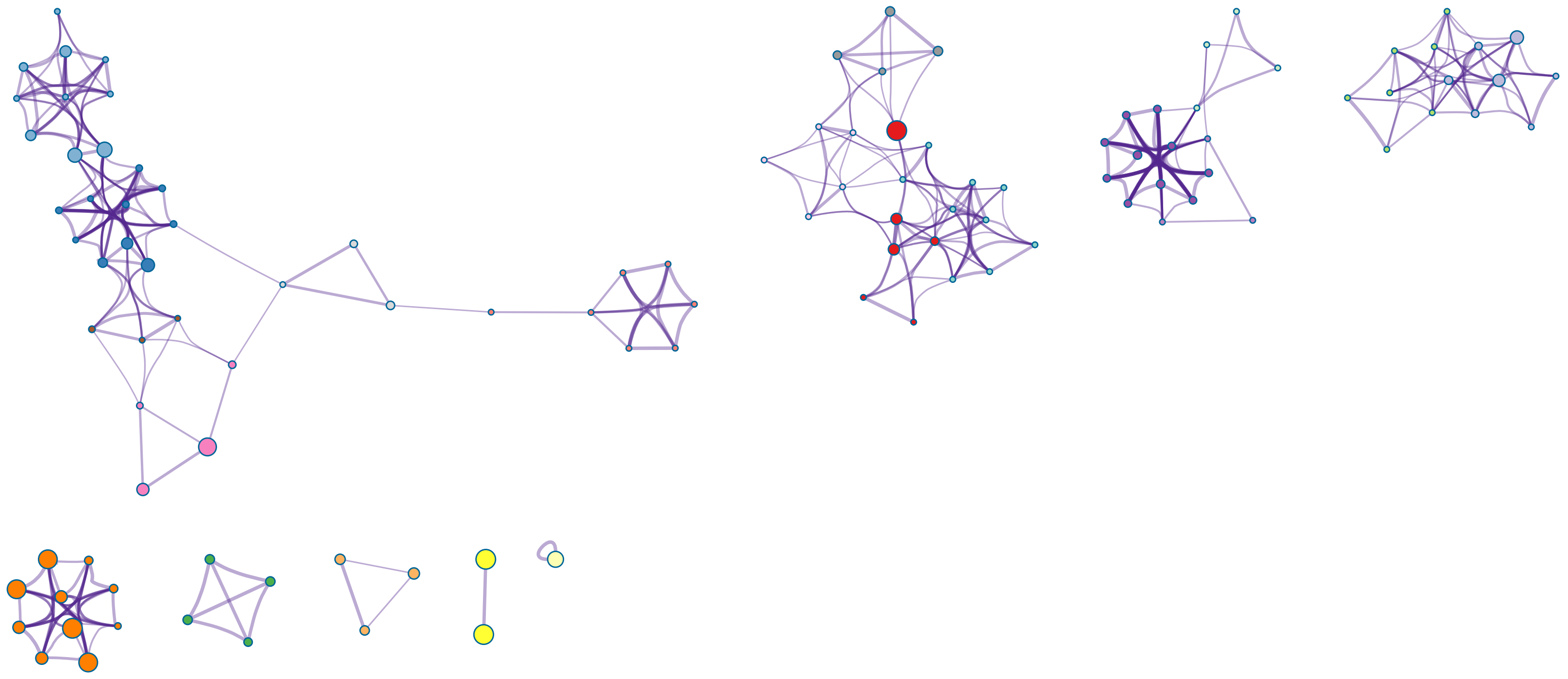

- oxidoreductase activity
- acetylcholine receptor activity
- carbonate dehydratase activity
- histone H3K14 deacetylase activity, hydrolytic mecha
- histone modifying activity
- kinase binding
- G protein-coupled acetylcholine receptor activity
- hydrolase activity, acting on ester bonds
- heme binding
- aldose reductase (NADPH) activity
- protein homodimerization activity
- endopeptidase activity
- aliphatic amine dehydrogenase activity
- monoatomic cation transmembrane transporter activit
- ubiquitin-like protein ligase binding
- laminin binding
- steroid dehydrogenase activity
- amide binding
- tau protein binding
- core promoter sequence-specific DNA binding

created by
http://metascape.org
