# Supplementary material for: Investigation of the Mechanism of Cinnamaldehyde in Irritable Bowel Syndrome Based via Network Pharmacology, Molecular Docking, and Animal Experiments
Source: Pediatr Discov. 2025 Oct 5:e70017. Online ahead of print. doi: 10.1002/pdi3.70017 (PMC13398650; doi:10.1002/pdi3.70017)
Supplement: Supplementary file 1 — Supporting Information S1 [file PDI3-9999-0-s001.zip › Supplementary Materials/go kegg/mf/Enrichment_heatmap/HeatmapSelectedGO.pdf]

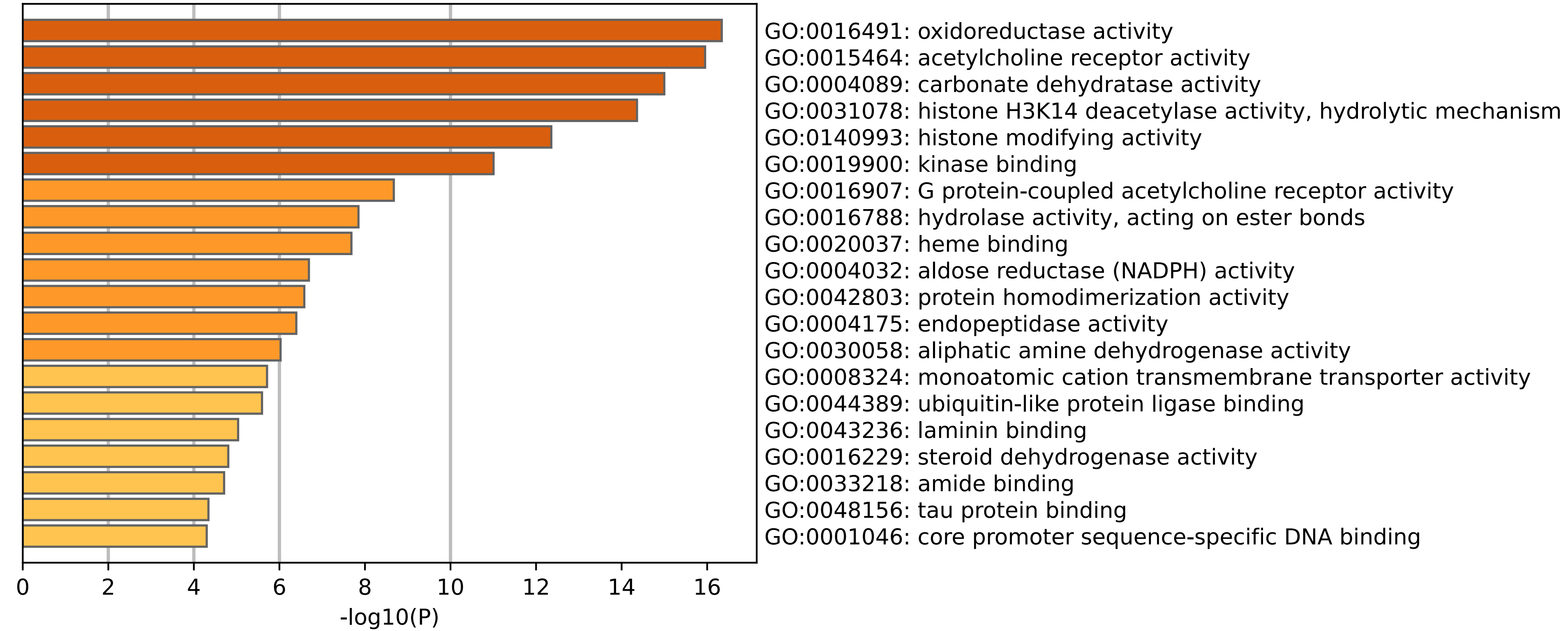
GO:0016491: oxidoreductase activity
GO:0015464: acetylcholine receptor activity
GO:0004089: carbonate dehydratase activity
GO:0031078: histone H3K14 deacetylase activity, hydrolytic mechanism
GO:0140993: histone modifying activity
GO:0019900: kinase binding
GO:0016907: G protein-coupled acetylcholine receptor activity
GO:0016788: hydrolase activity, acting on ester bonds
GO:0020037: heme binding
GO:0004032: aldose reductase (NADPH) activity
GO:0042803: protein homodimerization activity
GO:0004175: endopeptidase activity
GO:0030058: aliphatic amine dehydrogenase activity
GO:0008324: monoatomic cation transmembrane transporter activity
GO:0044389: ubiquitin-like protein ligase binding
GO:0043236: laminin binding
GO:0016229: steroid dehydrogenase activity
GO:0033218: amide binding
GO:0048156: tau protein binding
GO:0001046: core promoter sequence-specific DNA binding
0
2
4
6
8
10
12
14
16
-log10(P)
